# Genetic diversity and historical demography of underutilised goat breeds in North-Western Europe

**DOI:** 10.1038/s41598-023-48005-8

**Published:** 2023-11-25

**Authors:** Arianna Manunza, Johanna Ramirez-Diaz, Paolo Cozzi, Barbara Lazzari, Gwenola Tosser-Klopp, Bertrand Servin, Anna M. Johansson, Lise Grøva, Peer Berg, Dag Inge Våge, Alessandra Stella

**Affiliations:** 1 Institute of Agricultural Biology and Biotechnology, National Research Council, via Edoardo Bassini, 15, 20133 Milan, Italy; 2 GenPhySE, Université de Toulouse, INRAE, ENVT, 31326 Castanet-Tolosan, France; 3 Department of Animal Breeding and Genetics, Swedish University of Agricultural Sciences, Box 7023, 75007 Uppsala, Sweden; 4 Norwegian Institute of Bioeconomy Research, Gunnars vei 6, NO-6630 Tingvoll, Norway; 5 Faculty of Biosciences, NMBU, Norwegian University of Life Sciences, P.O. Box 5003, N-1432 ÅS, Norway

**Keywords:** Genetics, Population genetics

## Abstract

In the last decade, several studies aimed at dissecting the genetic architecture of local small ruminant breeds to discover which variations are involved in the process of adaptation to environmental conditions, a topic that has acquired priority due to climate change. Considering that traditional breeds are a reservoir of such important genetic variation, improving the current knowledge about their genetic diversity and origin is the first step forward in designing sound conservation guidelines. The genetic composition of North-Western European archetypical goat breeds is still poorly exploited. In this study we aimed to fill this gap investigating goat breeds across Ireland and Scandinavia, including also some other potential continental sources of introgression. The PCA and Admixture analyses suggest a well-defined cluster that includes Norwegian and Swedish breeds, while the crossbred Danish landrace is far apart, and there appears to be a close relationship between the Irish and Saanen goats. In addition, both graph representation of historical relationships among populations and f4-ratio statistics suggest a certain degree of gene flow between the Norse and Atlantic landraces. Furthermore, we identify signs of ancient admixture events of Scandinavian origin in the Irish and in the Icelandic goats. The time when these migrations, and consequently the introgression, of Scandinavian-like alleles occurred, can be traced back to the Viking colonisation of these two isles during the Viking Age (793-1066 CE). The demographic analysis indicates a complicated history of these traditional breeds with signatures of bottleneck, inbreeding and crossbreeding with the improved breeds. Despite these recent demographic changes and the historical genetic background shaped by centuries of human-mediated gene flow, most of them maintained their genetic identity, becoming an irreplaceable genetic resource as well as a cultural heritage.

## Introduction

Since their domestication beginning ca. 11,500 years ago in Southwest Asia, goats (*Capra hircus*) have been subjected to long-distance transportation from different geographic areas over thousands of years^1^. In particular, the islands were often used as strategic hubs of economic activity along the maritime routes and lots of domestic animals were translocated to islands to assure food availability^2^. Their small size and their remarkable versatility, adaptability and a greater resilience with respect to other ruminants^3^ made goats an ideal commodity, easy to be carried along land, maritime and riverine routes, following human displacements. The wool was used for warriors’ clothing and sailcloth^4^, making goats suitable not only for feeding but also for supplying the complex trading networks that were expanding. Over the past two centuries there has been an extensive transportation and intensive selection pressure on some very productive breeds at the expense of some traditional local breeds, which became neglected. These phenomena led to a dramatic reduction in their levels of genetic diversity, and in some cases pushed them to the brink of extinction^5^. The value of traditional breeds is recognised as providing a basis for future studies on diversity, domestication and the evolution of genes and traits of interest because they retain adaptive, specific alleles to thrive in their own environment^6^. Therefore, preserving the genetic variability and the uniqueness of indigenous goat breeds must be a priority in managing conservation and sustainable breeding programs.

The accessibility of cutting-edge genomic techniques, the large amount of genetic data available in public databases and the feasibility of fine-grained bioinformatic tools allowed researchers to move forward in the study of demography and evolution of several organisms and to dissect the complex process of domestication^3^. Several studies were performed on the evolutionary history of domesticated animals^7^, starting from their wild ancestors. In addition, the study of past introgression and gene flow among different breeds can help to describe their demographic history, defining admixture with foreign genetic contributions, crossing and inbreeding. Previous studies of worldwide goat breeds described a certain degree of genetic diversity among local breeds that belong to different and distant geographic areas^8–10^. A deeper examination of these results also suggests an unexpected level of similarity among the Northern-Western European breeds. Knowledge of the genetic diversity and the origin of Scandinavian goat breeds is still limited in comparison to sheep. Signatures of convergent domestication processes between the two species were described^11^. Thus, it is plausible that inference about the origin of Scandinavian sheep can inform about the origin of Scandinavian goats^5^. Cassidy and co-authors, combining ancient and modern mtDNA, indicated a close relationship between Irish and British Isles goats with Scandinavian breeds, suggesting a common origin occurred during the Viking colonisation. In our study, we used genome-wide SNP arrays to analyse the population data. The first objective entailed a genetic diversity study of local breeds belonging to our target area (Northern and Western European countries). Our second objective was to understand how gene flow and admixture events have shaped the genetic makeup of Scandinavian and Irish breeds, tracing back their origin. Nevertheless, our results can help to establish the “health status” of these local landrace goat breeds, identifying those which can be considered as the last modern archetypal ‘ancient’ type of goat. Indeed, these insights may be a starting point to discover the potential value of some breeds as a reservoir of historical genetic heritage. Finally, although with a certain caution, the goat genome could be considered a type of “bioproxy” – an organism that, through its close association with humans, shares a common colonisation history—such as the mouse genome, as proposed in previous studies^12, 13^. The use of the goat genome as a tool to indirectly infer the human phylogeography can become a complementary approach for discovering new clues about historical human land and maritime routes that are still uncertain.

## Results

### Descriptive statistics

In this study, the analyses were performed on two datasets. The first one included 10 breeds belonging to Northern and Western Europe (our target area and hereafter indicated by the acronym NW-EU): Icelandic landrace (ICL), Norwegian landrace (Norwegian—NRW, Norwegian coastal—SEL, Norwegian coastal—SKO), Swedish landrace (SWE), Danish landrace (DNK), Finnish landrace (FIN), Irish landrace (Old Irish Goat—OIG, Bilberry—BLB, Traditional Aran—ARR). In the second one, a wider dataset (indicated by the acronym WHOLE), we added breeds from several countries—The Netherlands (NLD), Normandy (Fosseé—FSS), Northern and Southern Spain (Bermeya -BEY, MLG – Malagueña) and the South of Italy (Girgentana—GGT, Ciociara Grigia – CCG, Jonica – ION), as well as Alpine (ALP), Saanen (SAA) and Toggenburg (TOG), with a total of 24 breeds (see “Material and methods” for details).

Pairwise F_ST_ and the Reynolds’ distance matrices for the WHOLE dataset are shown in Supplementary Table S1, whereas the heterozygosity levels (Obs-H/ Exp-H) and the genomic inbreeding coefficient (FROH) calculated for the target dataset are summarised in Supplementary Table S2. The genetic distance among all breeds ranged from 0.01 (Alpine breeds) to 0.48 (ICL vs SKO), and generally the values for the pairwise comparisons directly correlated with geographical distances. In the reduced dataset (NW-EU), the Swedish landrace displays less differentiation against all the Scandinavian breeds in comparison to the other breeds. The lowest is between SWE and SEL (0.069). A general overview of the heterozygosity (H) estimates indicates that all the NW breeds have a heterozygosity slightly below 0.4, suggesting a good level of genetic diversity. ICL had the lowest value (0.3), while the NRW, OIG and BLB show Obs-H values higher than expected (Exp-H), indicating less variability probably due to a demographic history characterised by founder effect, isolation and inbreeding as underlined by previous studies^10^. For NRW and the Irish breeds we might suspect an isolate-breaking effect (the mixing of two previously isolated populations). The highest levels of inbreeding calculated at the whole genome level (FROH) characterise the populations from the islands (ARR, SKO and ICL), indicating a limited gene flow with the populations from the mainland, while the Swedish landrace shows very low levels of inbreeding. The FROH estimates at individual level indicate that in DNK breed, some animals stand out for their greater values (see Supplementary Table S3), increasing the mean value of the estimate and highlighting a greater heterogeneity. The rest of the breeds show low or medium inbreeding levels.

### Population structure and genome-wide inference of individual ancestries

**Figure 1:**
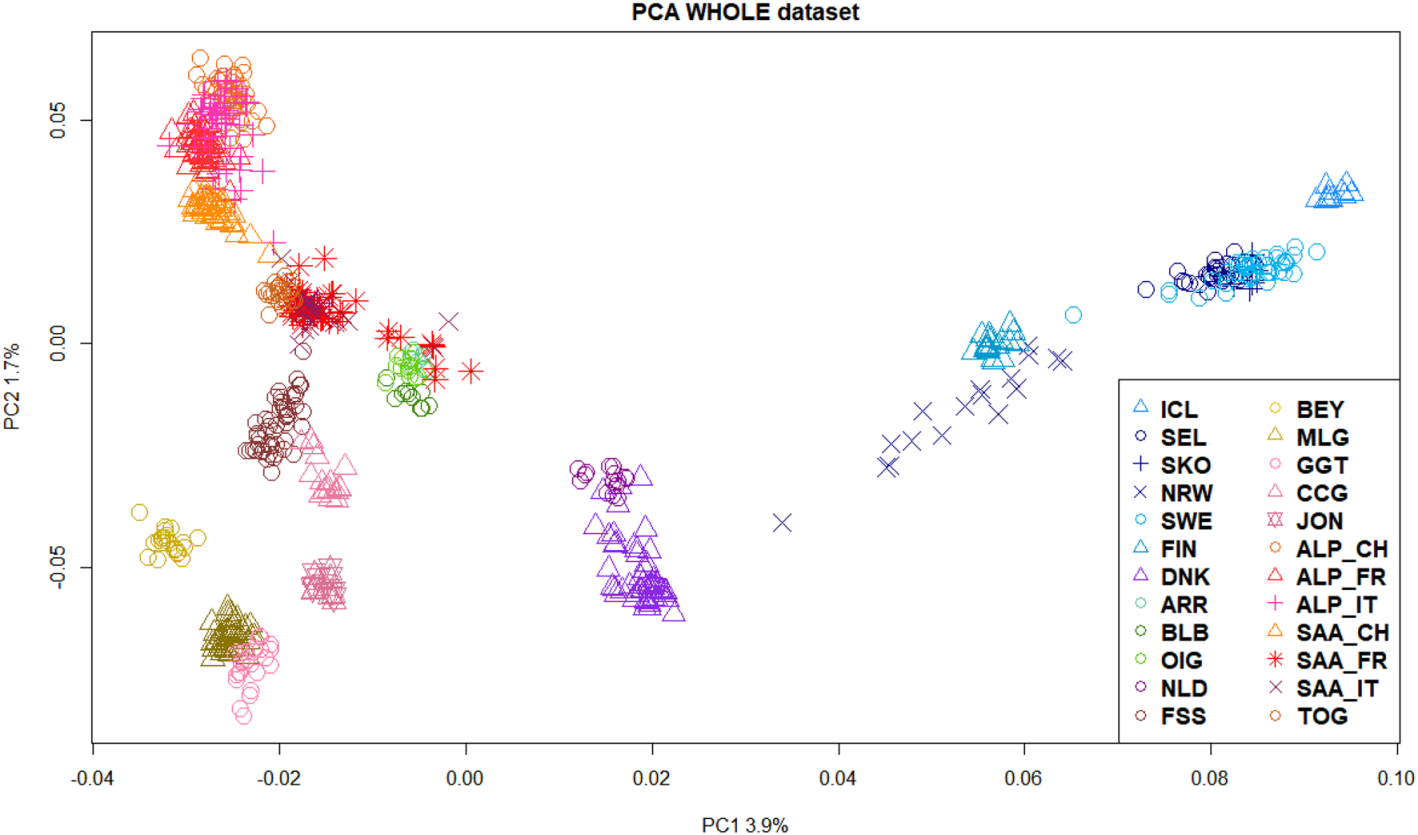
Principal component analysis (PCA) of allele frequencies that shows the population structure and the relationship between the twenty-four goat breeds analysed in the WHOLE dataset. Breeds acronyms according to Supplementary Table S8: Norwegian landraces (SKO, SEL, NRW); Irish landraces (ARR, BLB, OIG); Icelandic Landrace, ICL; Finnish Landrace, FIN; Swedish Landrace, SWE; Danish Landrace, DNK; Dutch Landrace, NLD; French breeds (Fosseé, FSS; Alpine, ALP_FR; Saanen, SAA_FR), Swiss breeds (Alpine, ALP_CH; Saanen, SAA_CH; Toggenburg, TOG); Spanish breeds (Bermeya, BEY; Malaguena, MLG); Italian breeds (Girgentana, GGT; Ciociara Grigia, CCG; Jonica, JON; Alpine, ALP_IT; Saanen, SAA_IT).

The principal component analysis (Fig. 1) demonstrates a clear separation among some breeds: the Scandinavian landraces are distributed in four groups: NRW very close to the well-distinct FIN breed, the two coastal Norwegian populations together with the Swedish landrace and the isolated ICL. NLD is clustering with some Danish goats, between the Scandinavian group and the Southern European and Irish breeds.

**Figure 2:**
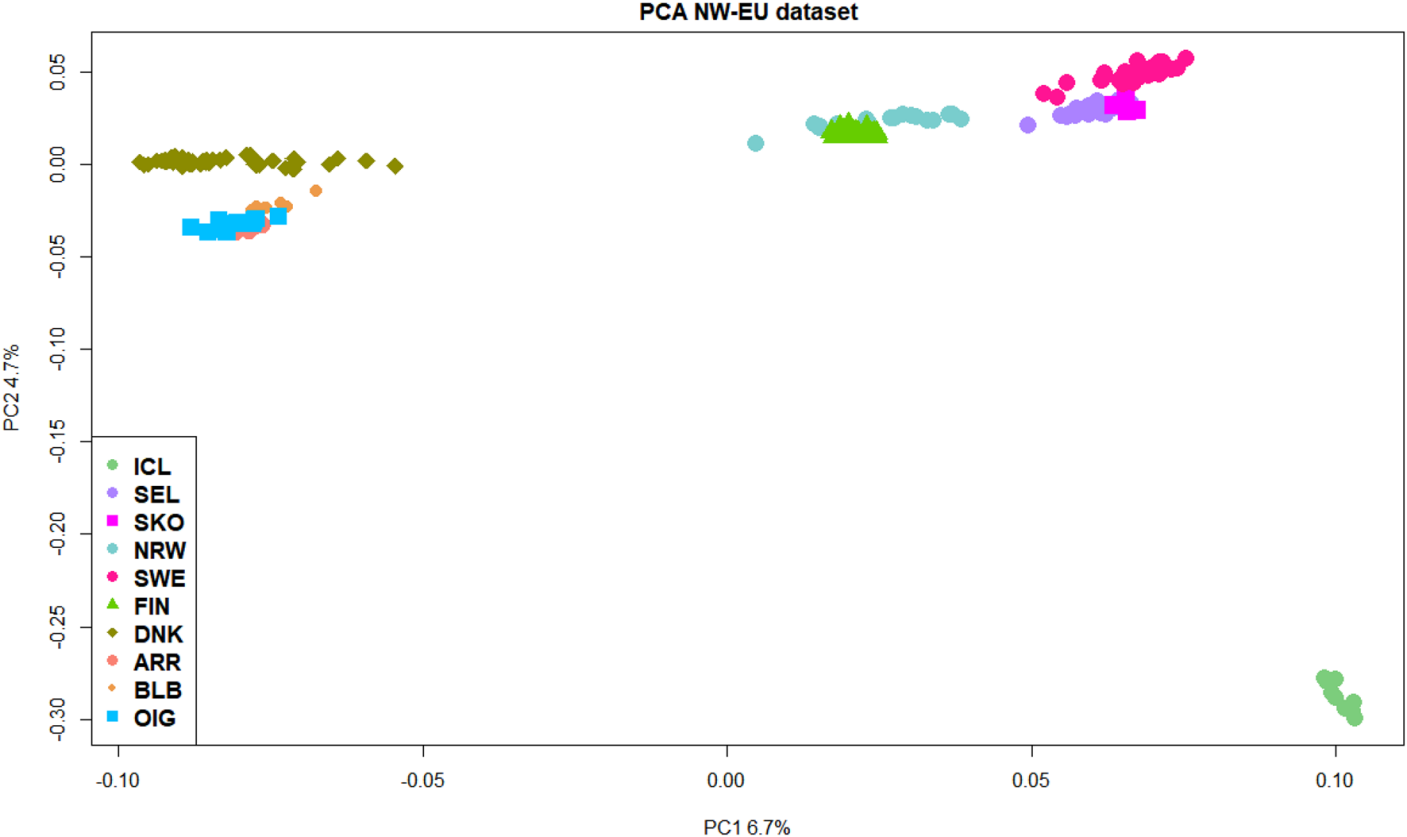
Population structure and relationship. Principal component analysis (PCA) that focuses on the relationship between the NW-EU populations dataset. Breeds acronyms according to Supplementary Table S8: Norwegian landraces (SKO, SEL, NRW); Irish landraces (ARR, BLB, OIG); Icelandic Landrace, ICL; Finnish Landrace, FIN; Swedish Landrace, SWE; Danish Landrace, DNK.

These last ones are close to some Saanen individuals, the CCG and FSS. Quite surprisingly, the Italian GGT goats make almost a unique group with the Spanish MLG. Figure 2 allows to focus only on the genetic relationship within our target NW-EU breeds, accounting for a greater genetic variance (8.6 and 18.1, relative to the first four PCs, Supplementary Figs. 1 and 2). This second PCA highlights four groups where the SWE is always clustering with the Norwegian coastal populations. The genetic affinity between FIN and NRW and among DNK and the Irish breeds (BLB, OIG, ARR) is here more evident, as well as the distance of ICL from the rest of Scandinavian breeds.

**Figure 3:**
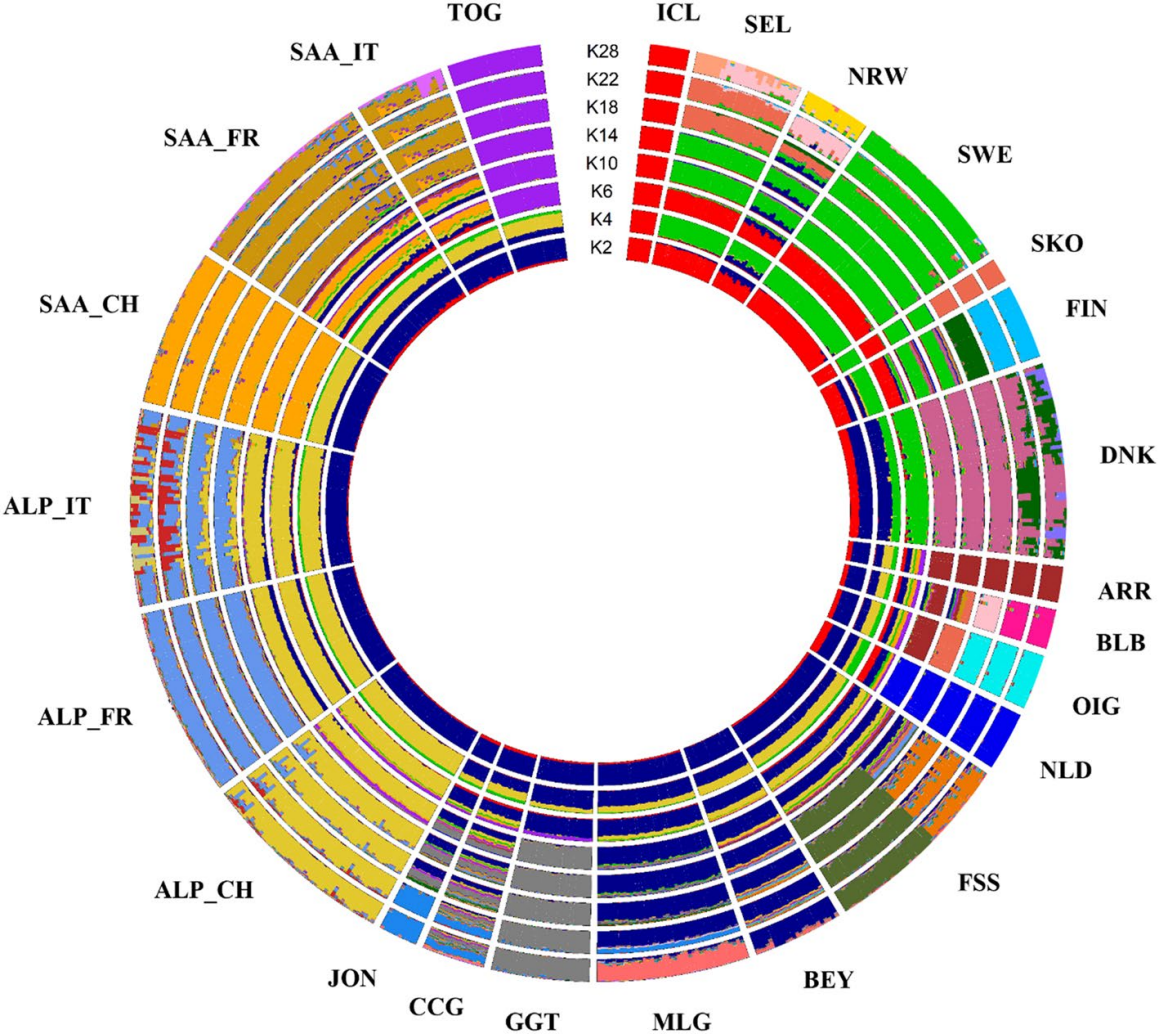
Model-based clustering. Circular representation of Admixture software results for K = 2, 4, 6, 10, 14, 18, 22 and 28, where K22 indicates the best estimate of number of genetic components based on the lowest cross-validation error value. Breeds acronyms according to Supplementary Table S8: Norwegian landraces (SKO, SEL, NRW); Irish landraces (ARR, BLB, OIG); Icelandic Landrace, ICL; Finnish Landrace, FIN; Swedish Landrace, SWE; Danish Landrace, DNK; Dutch Landrace, NLD; French breeds (Fosseé, FSS; Alpine, ALP_FR; Saanen, SAA_FR), Swiss breeds (Alpine, ALP_CH; Saanen, SAA_CH; Toggenburg, TOG); Spanish breeds (Bermeya, BEY; Malaguena, MLG); Italian breeds (Girgentana, GGT; Ciociara Grigia, CCG; Jonica, JON; Alpine, ALP_IT; Saanen, SAA_IT).

The estimates of global ancestry performed with ADMIXTURE are in Fig. 3 starting from the lower Ks, a clear subdivision according to two main genetic components: a well distinct Scandinavian-like component (red) and a South-western-European component (dark blue) that gradually separates into a more complex structure as the K values increase. The Irish and Dutch breeds present both components until k = 6, then showing a more specific “North-Atlantic-like” ancestry. The Bilberry goats (BLB) still present a small level of admixture with Saanen at K = 14 but also a “Norwegian- and Spanish-like ancestry” (K = 14–18). From K = 22 (that is the most probable number of ancestries by cross validation method, Supplementary Fig. 3A,B) almost all breeds are well characterised with a few exceptions still retaining some level of admixture (i.e. DNK, CCG). The Swedish goats share a common genetic composition with the Norwegian coastal goats from the mainland of West Norway.

### Effective population size and demography

**Figure 4:**
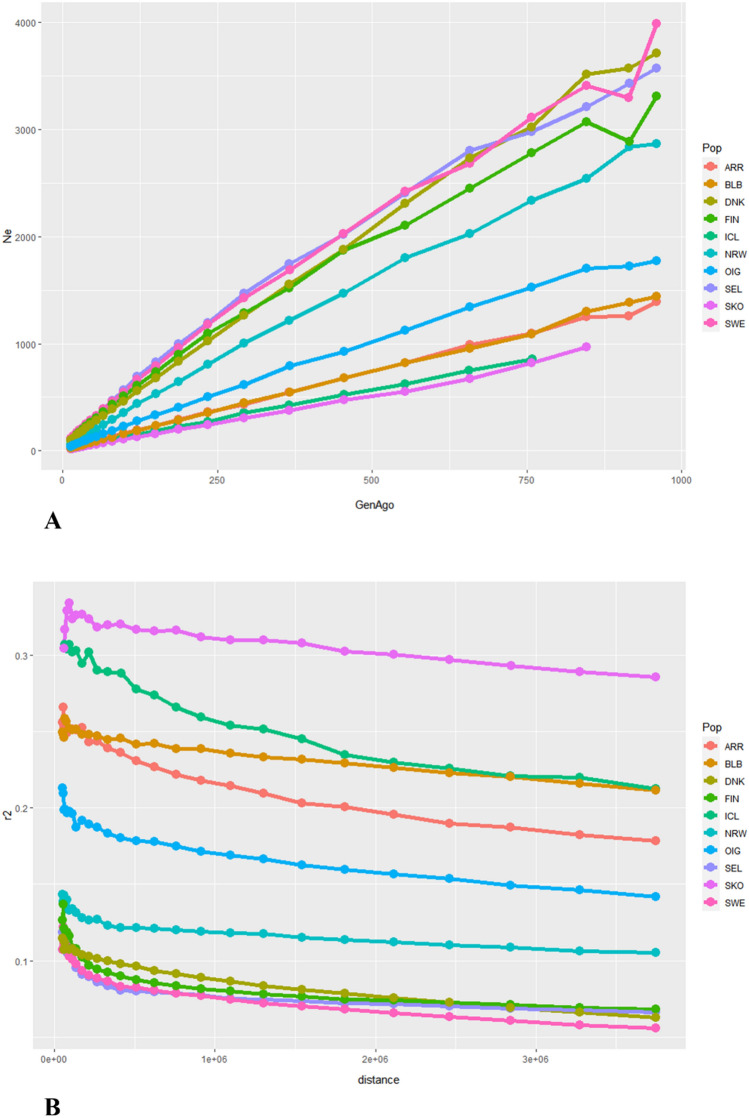
Effective population size and past demography. (**A**) Historical trends of Ne across generation in the past using the NW-EU target dataset. The x-axis indicates the generations, and the Ne estimates are indicated in the y-axis. (**B**) LD decay curves generated plotting the r2 (Linkage disequilibrium value) values against loci distance. Breeds acronyms according to Supplementary Table S8: Norwegian landraces (SKO, SEL, NRW); Irish landraces (ARR, BLB, OIG); Icelandic Landrace, ICL; Finnish Landrace, FIN; Swedish Landrace, SWE; Danish Landrace, DNK.

Focusing on the target breeds, the estimates of Ne over past generations and the LD Decay curves for each breed are shown in Fig. 4A and B.

Over the last 1000 generations, the Ne displays a decreasing pattern across the NW European breeds, halving their estimates since about 500 generations ago and with a steeper slope since the last 200. Lower Ne estimates were found in the Irish breeds together with SKO and ICL. In particular, the program fails to estimate the Ne in the Icelandic landrace since about 750 generations back in time. Among the Scandinavian breeds, SWE and FIN seem to have experienced a reduction, decreasing their Ne about 900 generations ago. In Fig. 4B we can observe the highest value of r2 for the Norwegian coastal population SKO and ICL followed by the two Irish breeds BLB and ARR. The mean r2 decreases less rapidly with the increasing genetic distance between markers but shows a pronounced decline in the ICL goats. Overall, the rest of populations have similar trends with the lowest decay for SWE.

**Figure 5:**
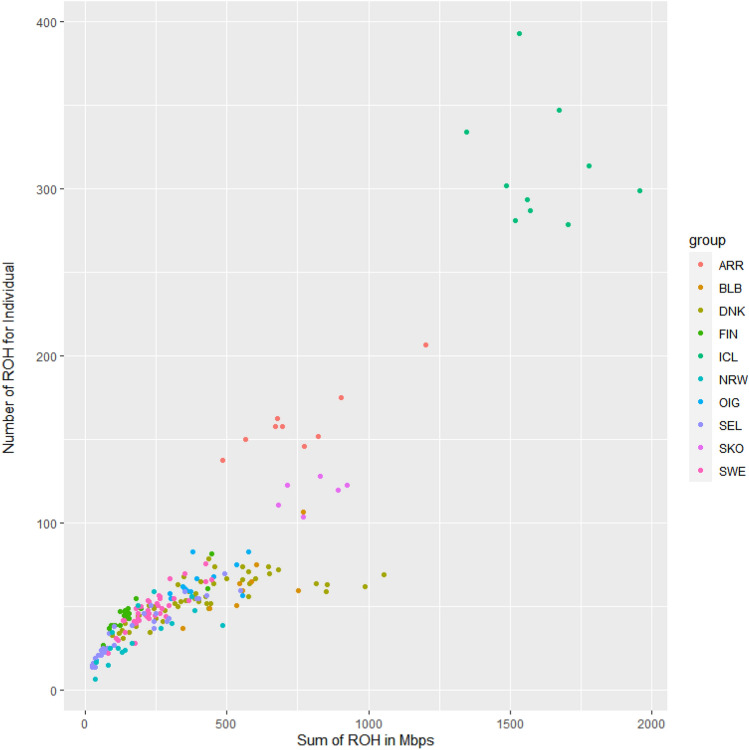
Runs Of Homozygosity analysis (ROH). Total proportion of the genome covered by homozygous segments. Each dot represents one individual and each breed coloured according to the colour code illustrated in the legend. Breeds acronyms according to Supplementary Table S8: Norwegian landraces (SKO, SEL, NRW); Irish landraces (ARR, BLB, OIG); Icelandic Landrace, ICL; Finnish Landrace, FIN; Swedish Landrace, SWE; Danish Landrace, DNK.

Figure 5 and Supplementary Fig. 4A–C illustrate the analysis of autozygosity levels and distribution of Runs of Homozygosity.

The smaller populations (or number of individuals) such as ICL, ARR and SKO, display more homozygous stretches and a bigger proportion of the genome covered by ROH, in comparison with breeds of larger size. In addition, these breeds have very long stretches (> 16 Mb) and also a higher number of ROH falling in the 2–4 class of length. Moreover, the Irish breeds carry a greater number of ROH included in the medium-length class (> 4–8). FIN, SWE and SEL have a smaller proportion of the genome covered by homozygote stretches, in agreement with the FROH values (Supplementary Table S2 and Supplementary Fig. 4C) and a frequency of ROH per classes and chromosomes (Supplementary Fig. 4A,B) with similar patterns among them.

### Phylogeographic and TreeMix analysis

**Figure 6:**
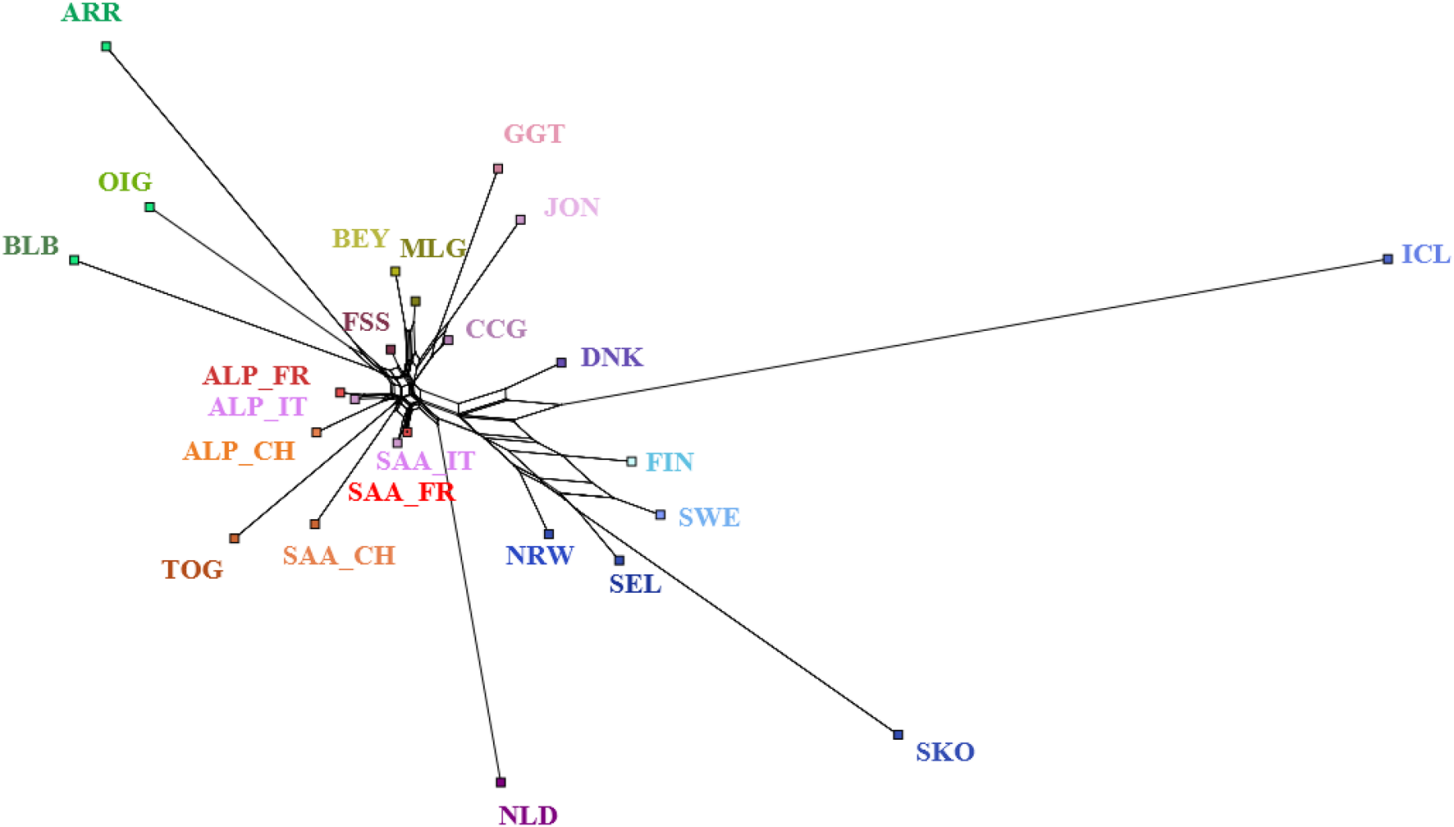
Inferred unrooted tree from a neighbour-joining analysis of Reynolds’ distance data. The Neighbor-Net graph displays the reticulate relationship among the analysed populations. Each terminal branch represents one breed. Breeds are labelled according to breed grouping in Fig. 1. Breeds acronyms according with Supplementary Table S8: Norwegian landraces (SKO, SEL, NRW); Irish landraces (ARR, BLB, OIG); Icelandic Landrace, ICL; Finnish Landrace, FIN; Swedish Landrace, SWE; Danish Landrace, DNK; Dutch Landrace, NLD; French breeds (Fosseé, FSS; Alpine, ALP_FR; Saanen, SAA_FR), Swiss breeds (Alpine, ALP_CH; Saanen, SAA_CH; Toggenburg, TOG); Spanish breeds (Bermeya, BEY; Malaguena, MLG); Italian breeds (Girgentana, GGT; Ciociara Grigia, CCG; Jonica, JON; Alpine, ALP_IT; Saanen, SAA_IT).

The distance-based NeighborNet algorithm implemented in SplitsTree allows for collections of clusters that overlap and do not form a hierarchy as in a general phylogenetic tree. In Fig. 6, the connections among the Scandinavian breeds with all neighbour-net edges relating to this group are provided.

**Figure 7:**
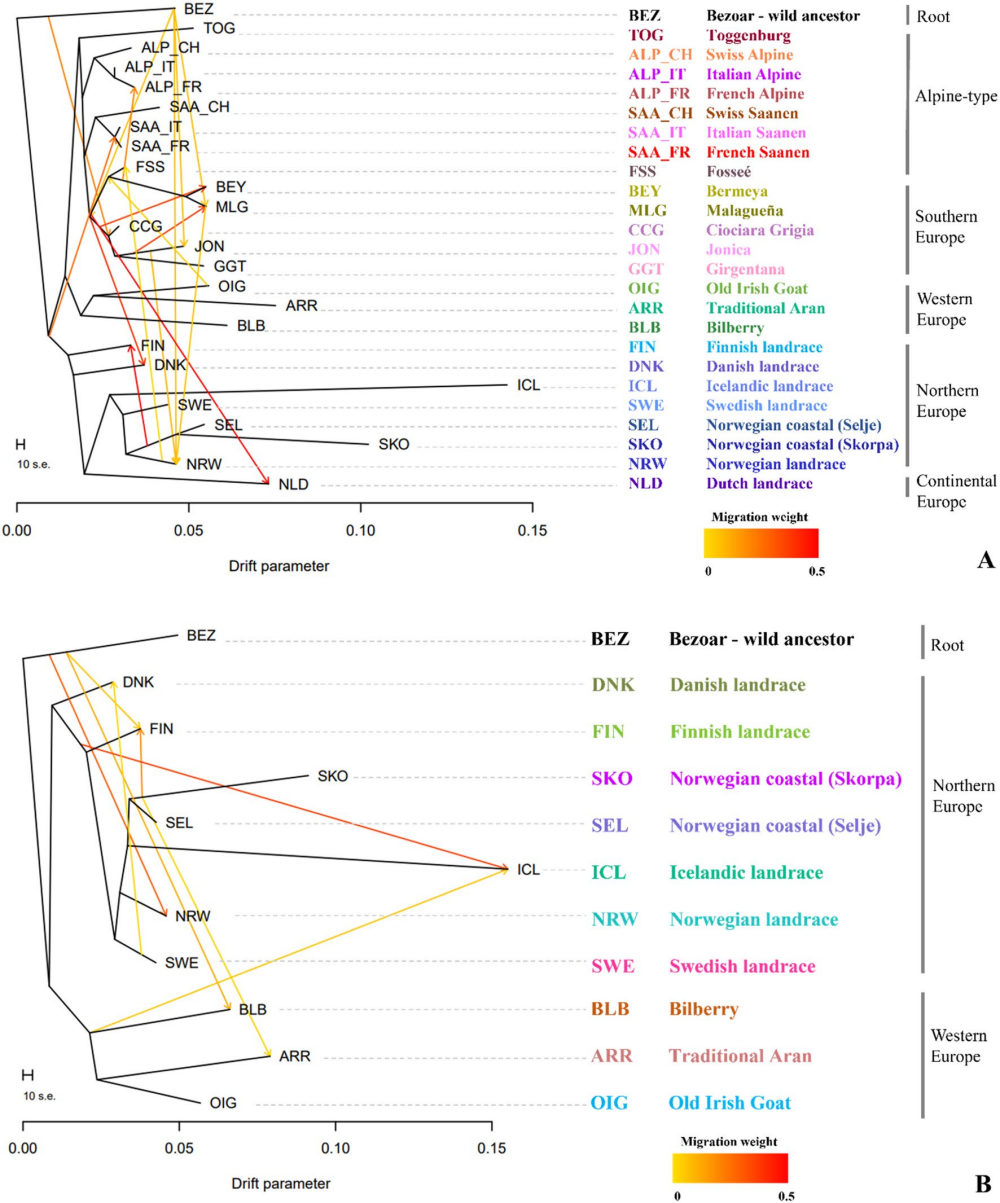
(**A**,**B**) Comparison between the two analyses carried out with the TreeMix program on the two datasets. To the left of the figure, (**A**) refers to the WHOLE dataset for m = 15 and (**B**) to the NW-EU target dataset for m = 8. The migration edges indicate the direction of the gene flow between two breeds and the colour of the arrow is according to the weight of the migration event. Breeds acronyms according to Supplementary Table S8, and their colours correspond to those in Figs. 1 and 2; Norwegian landraces (SKO, SEL, NRW); Irish landraces (ARR, BLB, OIG); Icelandic Landrace, ICL; Finnish Landrace, FIN; Swedish Landrace, SWE; Danish Landrace, DNK; Dutch Landrace, NLD; French breeds (Fosseé, FSS; Alpine, ALP_FR; Saanen, SAA_FR), Swiss breeds (Alpine, ALP_CH; Saanen, SAA_CH; Toggenburg, TOG); Spanish breeds (Bermeya, BEY; Malaguena, MLG); Italian breeds (Girgentana, GGT; Ciociara Grigia, CCG; Jonica, JON; Alpine, ALP_IT; Saanen, SAA_IT).

Although quite complex, the relationship between them is clearly represented as a well-distinct group. There is less network-like splitting in the Southern-European side of the Network: breeds are close to each other even if remaining separated according to their geographic provenance. Also, the Irish breeds cluster in a unique group with long edges separating one another. The TreeMix analysis (Fig. 7A and B) allows to both build a phylogenetic tree that visualises the genetic distances among breeds while testing if past gene flows are detectable.

We checked for a number of migration events ranging from 1 to 15 (Supplementary Fig. S5A, for the complete dataset) and from 1 to 10 (Supplementary Fig. S5B, for NW-EU dataset). In the case of the complete dataset (Fig. 7A, Supplementary Fig. S5A), the residuals’ curve (R2) against the number of migrations does not reach the minimum value recommended by the authors of the program (in our analysis is 0.991 instead of 0.998, Supplementary Table S4A)^14^. Some events of gene flow are still worth closely checking (Supplementary Table S4B). For example, the vector with the highest weight connects BEZ (bezoar, the ancestor) with GGT (~ 0.55 edge weight) followed by the Spanish-Italian branch to DNK (edge weight 0.45). From the branch of the Irish breeds to the French FSS we observe an edge with a weight of 0.38, from the Norwegian SKO to the Finnish FIN an edge weight of 0.26 and from the Spanish BEY towards the Irish OIG another migration event with a weaker contribution (~ 0.07). Another interesting event comes from the Norwegian branch to FSS and from the Southern breeds to the Norwegian branch.

The case of the NW-EU dataset is different (Fig. 7B, Supplementary Fig. 4B), where starting from the second migration edge, the residuals’ curve reaches the critical value of 0.99 and from the sixth onwards it reaches 0.998 (Supplementary Table S5A). The maximum value is accounted for m = 8, and vectors 9–10 bring only a small increase in R^2^ value. A closer inspection of the results (Fig. 7B) together with the model-fit analysis carried out with BITE (Supplementary Table S5B) highlights several interesting links among populations: there are two gene flow events both towards the Icelandic landrace ICL from the Irish BLB and from the branch that connects DNK with the rest of the Scandinavian breeds (edge weight of 0.06 and 0.37, with a significant p-value on the edge weight of 0.02 and 7.40785e^–10^, respectively). From the ancestor BEZ—or a breed very close to it—there are three migrations events, towards NRW, FIN and BLB; the last significant vector indicates gene flow from the Norwegian coastal SKO towards FIN (edge weight 0.19, p-value 1.7953e^–06^).

### Test for admixture and past gene flow

To evaluate the presence of admixture and gene flow, we further computed the f3 and f4 statistics. We did not observe significant results for the f3 test in either of the two datasets. Interestingly, we obtained some significant results using the f4 statistic. Following the explanations of the authors^15^, if all three permutations of f4-statistic for a certain set of four populations are (significantly) non-zero, then at least one of the populations must be admixed; this is one of the most common signals of admixture used in the literature. Thus, out of 37,950 possible tree topologies tested, we selected the most valuable results (following the criteria detailed in methods), summarising them in Supplementary Table S6A and B. Among the most significant results (38 triplets with − 20 < Z-score >  + 20, in Supplementary Table S6A) we can observe that some of them support our previous analysis (ADMIXTURE, NeighborNet, TreeMix). In particular, by comparing the TreeMix analysis (Fig. 7A), the results suggest the populations of GGT|SWE, GGT|NRW, only GGT or populations very close to them, as sources of admixture for DNK. In addition, results would indicate the Scandinavian breeds SWE, FIN, NRW, SEL and the Spanish BEY as possible candidates that contribute to the Irish OIG (like the migration event highlighted by TreeMix).

In the second step of the screening process, we focused only on the NW-EU breeds, excluding all possible “Alpine-Saanen influx” that could mask or confuse the signal of past admixture that we are looking for. Thus, we selected only the triplets with highly significant (28 out of 125, p-value < 1.00E-04) non-zero values, summarising them in Supplementary Table S6B. Although f4 statistics cannot, in general, explicitly identify which of the four populations is admixed without additional information, some points can be taken into account. For example, several f4 scores indicate a genetic contribution that can originate from any Scandinavian breed towards all the Irish and the Icelandic landrace. The migration event detected with TreeMix that originates from the root (the ancestor BEZ) towards BLB is also suggested by the significant f4 scores, but it could also be that BLB is admixed with most of its ancestry with the Norse breeds and a small amount related to BEZ. Furthermore, we can point out several interesting admixture events that involve all the Irish breeds and the Icelandic goats with a similar introgression pattern and high Z-scores (Supplementary Table S6B), even if we did not find any clear evidence of admixture in the Traditional Aran goat (ARR).

The admixture proportions estimated via f4-ratio support the past gene flow among the study target populations. As we can observe in Supplementary Table S7, using the two Norwegian coastal breeds as the closest proxy of the original source of introgression in ICL, we obtain highly significant estimates either including FSS, BEY or the two Irish BLB and OIG (22%, 24%, 26% and 29% respectively, Z-score > 9). The estimate is still significant when ARR is used as a proxy for the non-admixed population (29%, Z-score > 9). In addition, when including the Swedish landrace as an alternative source in the estimate of f4-ratio between SEL|SWE|ICL, the proportion of admixture is lower but still significant (13–15%, Z-score > 4.5), confirming the Norwegian-like ancestry component into the genome of ICL and indicating the weak or null Swedish contribution. For the Irish breeds we found three main ancestry proportions: a Scandinavian-like, a Spanish-like and signs of introgression from the Saanen breed. The f4-ratio quantified the Spanish-BEY proportion in the BLB goats between 42 and 48%, the Scandinavian proportion is 47–65%, while a 20–22% of Saanen. The Spanish-BEY, Scandinavian and Saanen ancestry contribution to the OIG genome is 18%, 21%, and 12%, respectively.

### Weighted LD decay and time of admixture events

The weighted linkage disequilibrium decay curves method implemented in the program ALDER was used to test some scenarios suggested by f4 and f4-ratio and to infer the admixture time that led to the genesis of Icelandic goats, and the possible contribution of Norse breeds in shaping the genetic make-up of Irish goat breeds. The weighted LD decay curves depicted in Supplementary Fig. 5 show an exponential decrease that indicates admixture among the population target, and two possible sources from which this introgression derived. We describe only the significant estimates corresponding to the successful tests of admixture and we indicate them in years. For Icelandic landrace, the three curves computed matching NRW|SEL, NRW|ICL, SEL|ICL weights exhibited a statistically significant signal of exponential decay (p-value < 0.2) concurring in their decay rates (~ 1100 years ago, Supplementary Fig. 6A). No other comparisons were significant for the ICL goats. For the Irish breeds, we obtained successful tests when considering both Scandinavian (NRW|SWE) and Southern European breeds (FSS|BEY) as donors. This last test is intriguing because it likely corresponds to distinct time periods when the gene flow took place. The most significant decay rates involved NRW and SWE (supporting the above f4-ratio results), with a midpoint of admixture of about 1300–960 years ago (~ 700 CE- 1040 CE) for BLB|NRW|OIG (p-value < 0.021, Supplementary Fig. 6B) and for OIG|NRW|SWE goats (p-value < 0.01, Supplementary Fig. 6D–E). The most ancient event appears when we consider FSS and BEY as representative reference populations for BLB (~ 2,300 years ago/ ~ 300 BCE; p-value = 0.04, Supplementary Fig. 6C).

## Discussion

We analysed patterns of genetic diversity between Scandinavian and Irish goat breeds using publicly available genotypes and new data generated for a Swedish landrace population. We aimed to dissect the genetic status of the Swedish landrace in comparison with other Northern-Western European breeds. We found signs of introgression of Norwegian alleles in the genome of Icelandic and Irish breeds and further investigated these patterns by inferring the gene flow and admixture events that occurred in the past. These putative events were tested by f4 statistics^15, 16^ and subsequently, the admixture proportions and the time of admixture were inferred with the f4-ratio and the exponential LD decays. The first two components of Principal Component Analysis clearly separate the “Scandinavian” group from the “Continental” and the “South-Western European” ones. The Swedish breed is clustering with the Norwegian coastal populations with a FST value of 0.069 and a common ancestry is supported by Neighbor-Net and ADMIXTURE results. The genetic background at the most significant value of inferred ancestries (K = 22) underlines again a well-defined and shared genetic component in SWE and SEL almost without any foreign contribution. In agreement with that, the demographic history of the Swedish population appears not to be characterised by bottleneck or recent inbreeding, and it confirms several aspects already detected by analysing the genetic distances, the heterozygosity and homozygosity level. Moreover, the SWE population looks like a possible reference population for the Icelandic goats when we explore the population history with overlaid migration events using TreeMix and the f4 statistic. The proportion of this introgression is about 13% (f4-ratio between SEL|SWE|ICL). We retrieved a signal of admixture from the donor SWE to the Irish OIG and another potential donor NRW towards both OIG and BLB, but not towards ARR. The reason why we did not find any sign of past introgression in ARR probably lies in the high level of isolation in comparison with the other two Irish populations^10^ that can erase signatures of other old demographic events. A small population size combined with a fragmented distribution probably also favoured the maintenance of high levels of inbreeding in the Icelandic, Skorpa and Aran populations, a point that emerges from our results. This is mirrored also by the very long Runs of Homozygosity (ROHs) that characterise these isolated breeds. However, all the Irish breeds also carry a greater number of ROH included in the medium-length class (> 4–8), that is related to populations that have undergone a population bottleneck^17^, as confirmed by previous data^18^. Focusing on the second PCA analysis that includes only the Northern-Western European group, the small genetic distance between the Danish and the Irish goats could be associated with both a common recent introgression with the Saanen breed (for improvement purposes) but also with an older relationship related to the Norse conquest of the Irish and British Isles^5^. This “Scandinavian-like” – mostly Norwegian – ancestry component was already detected using the ancient and modern mitochondrial DNA of feral and improved Irish goats^5^. These patterns of introgression are observed also in the Icelandic goats: in particular there are two migration edges in the Maximum Likelihood tree that link this breed to (i) the Irish Bilberry and (ii) the branch that includes all breeds of Nordic origin. TreeMix analysis also detects another migration event, congruent with f4 estimates, that indicates not only the Scandinavian breed but also the Spanish Bermeya as candidate reference populations for this gene flow event (BEY towards BLB). Bermeya is a small, endangered breed from Asturias, Northern Spain (census of ~ 2000^19^), a region that has been conquered by the Vikings during the Viking Age. However, also two human genetic studies about the origin of Celtic people also argued on the Spanish introgression in different timeframes into the Irish genome together with a Norwegian-like genetic component^20, 21^. The possible contribution of BEY as donor to the Irish breed is confirmed in the TreeMix analysis, f4-statistic, the f4-ratio and the LD-decay curve. In particular, the LD-decay rate (that corresponds to the time of admixture) is consistent with the Iron Age and the Celtic expansion during the Roman Empire (~ 366 BCE)^22^. The f4-ratio quantified the Spanish-BEY proportion between 42 and 48% (BEY to BLB) and 18% and 21% (BEY to OIG), which is also evident in the ADMIXTURE analysis. The SAA_FR introgression detected in a small proportion is probably recent and related to the introduction of the Saanen breed in improvement schemes^5^. The TreeMix reconstructions based on ancient and modern Icelandic horses^22^ revealed that the modern individuals were most closely related to a group of North European horses including pre-Viking Pictish horses from Britain (c.a. sixth-seventh century), Viking horses (c.a. ninth-tenth century) and with samples of the Gallo- Roman period. This is in line with the diaspora of Scandinavian seafaring warriors and traders, as well as with the expansion of the Roman Empire^22^. In addition, and in agreement with another human genetic study that investigates the genomic history of the Iberian Peninsula, the authors documented a consistent trend of increased ancestry related to Northern and Central European populations from the Iron Age onward. This genetic cline increases from 10–19% in individuals along the Mediterranean coast to 28–43% in the North of Spain where Indo-European Celtiberian languages were likely spoken^23^.

Those discoveries again confirm a complex network between the target study area during the history and corroborate the two gene flow events from Irish and Norwegian breeds towards Icelandic goats. The migration events of TreeMix, involving the Norwegian breeds and the Southern European populations (including the FSS from the Normandy) in both directions are once again probably linked to the trading routes during the Viking Age. A significant exponential LD decay is also detected in the Icelandic goats when we use the two Norwegian NRW|SEL as proxy to reference populations, providing further evidence for admixture. In this case, the admixture midpoint was estimated by ALDER program to have occurred ~ 1100 years ago, according to Refs.^24^ and^25^ that assign to Icelandic goats a North-European (most likely Norwegian) origin after being imported during the colonisation of Iceland. The first settlement in Iceland also included individuals with North-Atlantic-like ancestry as described in Ref.^26^, who analysed the whole genome sequences of 27 ancient Icelanders. They further showed that these individuals were markedly more similar to their source populations in Scandinavia and in the British-Irish Isles than to modern Icelanders, whose genomes have been shaped by 1100 years of extensive genetic drift^26^.

Previous studies performed on Icelandic sheep data identified patterns of admixture between other Scandinavian countries, assuming a Norwegian origin (thus descendant from Old Norwegian Short Tail Landrace) that fits well with historical records^27, 28^.

The last significant LD decay rates point to the Irish breeds BLB|OIG as receiving populations and the NRW and SWE as representative donors, and the admixture times range from ~ 1300 to 960 years ago. These dates indicate a slightly greater timeframe back to the past in agreement with the earlier invasions of Ireland in comparison to Iceland, covering a period of more of 300 years (ninth-twelfth century) from the first Viking raids to the Norman invasion^29^. The first two centuries of this period are characterised by Viking raids and the subsequent Norse settlements along the coast, with five ports established at Dublin, Wexford, Waterford, Cork and Limerick. A strong signature of ancestral Scandinavian influence in the Irish genomes is also recorded in Connacht^29^. This western province of Ireland (Connacht) includes the county of Mayo, where the Old Irish goats analysed in this study have been collected. The County of Waterford (in the southern province of Munster) is the county where Bilberry originates from. In addition, the Scandinavian ancestral component was discovered also in cattle, analysing mediaeval cattle remains from Dublin^30^.

A recent human genetic study aimed to assess the impact of historical migrations on the Scandinavian gene pool^31^ indicates a surge of gene flow from the British-Irish Isles into Scandinavia during the Viking period. This is perhaps not surprising, given the extent of Norse activities in the British-Irish Isles as said above. Indeed, raid and trade were often overlapping activities: the travellers were not only warriors but also farmers. The main trading commodities were jewels, slaves, metals, and farm animals. Studies on the wool used for sails in Viking ships demonstrated that at least 80% of analysed wool fragments were imported from outside of Denmark^32^. In addition to the above references, in a very recent study^33^ isotopic analyses of dogs and horses’ remains from different burial sites in the United Kingdom shed light on the Norse origin of the two species.

Altogether, our insights suggest that the genome of these modern landrace still retain some clues of past human migrations though this ancient footprint has been eroded by the introgression of improved breeds and demographic declines, as argued by^5^ and like it happened in humans^34^. For example, the Old Irish goats have been subjected to casual hunting, reduction of their grazing area for urban development and indiscriminate culling of feral herds, which has led this population to the brink of extinction^5^. Although these goat breeds already have been now genetically well characterised, many questions regarding their origin and breed development remain to be discovered. For this reason, it is worth further investigate them using a fine-grained approach, combining ancient DNA and modern samples, using a whole genome sequence approach and more specific bioinformatic tools, which will give a long-term view of the formation and evolution of these breeds.

## Conclusions

In light of these findings, we can argue that the Swedish goat gene pool is well characterised, with good genetic variability levels, almost purebred with a Nordic genetic background similar to the neighbouring Norwegian coastal goats. Our ancestry analyses are supported by patterns documented in human phylogeography studies, previous studies in sheep and horse and historical records, identifying traces of Scandinavian-related ancestries in the genome of Icelandic and Irish goat breeds compatible with maritime Viking migration routes. This journey in the footsteps of the Vikings brought us back to the past, following hidden clues in the genome of indigenous goats. Most of them are in a critical status of conservation, surviving in small herds threatened by the demands of intensive production or the loss of habitats. Irish and Scandinavian breeds constitute ideal candidates that deserve efforts for a specific conservation program. We highlight their importance as a historical genetic resource because they retain in their genome a weak but still detectable signal of an ancient gene flow related to the formation of large-scale trading and cultural networks that spread people, animals and commodities, and revealing the untold potential of some endangered, unutilised, traditional goat breeds.

## Methods

The scope of this study was to assess the genetic diversity and the demographic history of Northern-Western European goat breeds. In addition, we aimed to detect past migration events explaining the breed formation of modern autochthonous goats in this target region of Europe.

### Datasets construction and quality control

**Figure 8:**
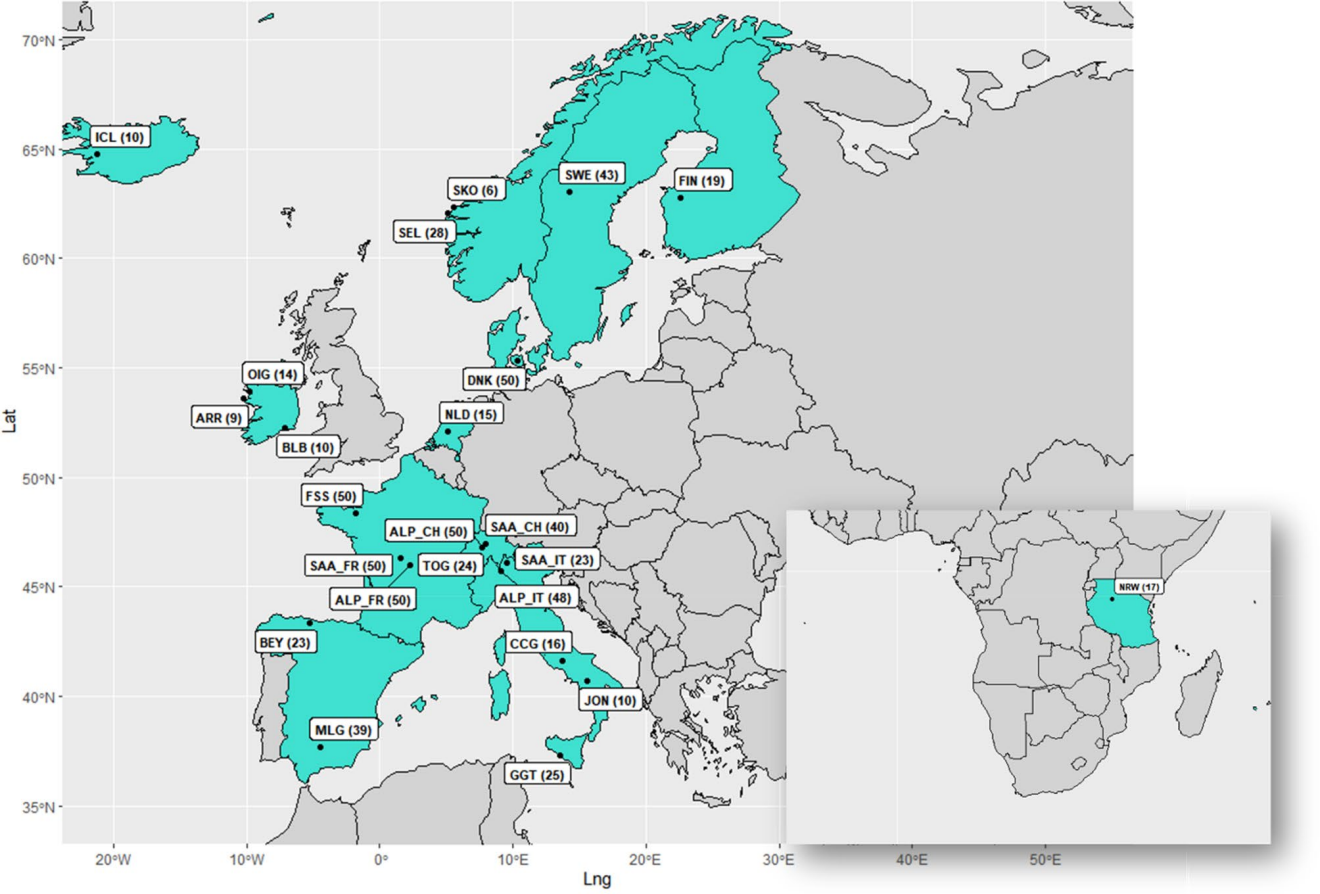
Sampling location for the 24 breeds analysed in the WHOLE dataset. Highlighted in turquoise are the countries the breeds are sampled from. The number of individuals is indicated in brackets. Other details in Supplementary Table S8.

All the details about the origin of data, breed, breed code, sampling country and geographical coordinates are in Supplementary Table S8^35–37^. In this study, two datasets were built: the first one constituted by 10 breeds and 206 goats belonging to Northern and Western Europe (hereafter indicated by the acronym NW-EU) which are our target populations. For the second dataset, indicated by the acronym WHOLE, we added breeds from The Netherlands, Normandy (Northern France), Northern and Southern Spain and the South of Italy, as well as Alpine, Saanen and Toggenburg, with a total of 24 breeds and 669 goats. Sampling location is detailed in the map (Fig. 8).

We included the transboundary breeds since in the last decades they have contributed in a small fraction to the genome of Irish and Danish landraces, as suggested by previous results^5, 8^ and from the Domestic Animal Diversity Information System (DAD-IS, https://www.fao.org/dad-is/browse-by-country-and-species/en/). Another technical reason for including cosmopolitan breeds is to deal with the ascertainment bias of the SNP chip. We also took into consideration the results from Ref.^38^, in which the Old Irish Goat (OIG) breed is closely related to the Southern Italian breeds as highlighted by the Neighbour-Joining tree and historical data about the migration routes during the Viking Age (750–1050 CE)^31^. Thus, the wider dataset (WHOLE) includes goat breeds that belong to countries of the ancient Vikings’ routes, from the continental Europe (the Netherlands) and from Southern-Western Europe along the Atlantic coast and part of the Mediterranean (Normandy, Northern and Southern Spain, Southern Italy).

All goats have been genotyped using Illumina GoatSNP50 BeadChip^39^. In particular for the Swedish Landrace, the sample collection, DNA extraction and the genotyping methods have been previous described in Ref.^40^. After merging, the SNP data were remapped against ARS1.2 assembly and the quality control was performed using PLINK v1.9^41^, following the FAO guidelines for the genomic characterisation of animal genetic resources^42^. We excluded the variants that were unmapped, mapped on the sex chromosomes and on the basis of missing call rate per SNP (–geno < 0.05), of the minor allele frequency (–maf > 0.01) and of the individual missingness (–mind < 0.05). In addition, we performed a LD-pruning (Linkage Disequilibrium) using –indep-paiwise 50 5 0.2, (i) to reduce the bias due to the ascertainment^43^ and (ii) to remove individuals with extreme observed heterozygosity. The command –king cat-off in PLINK v2.0 was used to remove closely related (and possible duplicate) animals. This procedure left 35,375 SNPs for the subsequent analyses. Finally, we used BITE^44^ for reducing the unbalance among populations and retaining individuals from 5 to 50 on the basis of population structure. To convert these data into a format suitable for different input files, we used PGDSpider v2.0.4.0^45^ for PLINK to Arlequin^46^ and CONVERTF (included in EIGENSOFT v3.0^47, 48^, for PLINK to EIGENSTRAT format.

### Population structure and demography

We used Arlequin v.3.5^46^ to calculate the pairwise F_ST_ values and the Reynolds’ genetic distance (Supplementary Table S1A and B). To perform Principal Component Analysis (PCA) we used SNPrelate package v 4.1.2^49^ and individual ancestries proportion were inferred using the unsupervised clustering method in ADMIXTURE v.1.3^50^ with a number of clusters K ranging from 2 to 28 for the WHOLE dataset. The most probable number of K was inferred by cross-validation method and all the results were plotted using the “membercoeff.cv” and “membercoeff.circos” functions in BITE. Historical trends in effective population size (Ne) and the LD decay curves were estimated with the SNeP software^51^ with default settings and a correction to adjust the LD for small sample sizes. The genomic inbreeding coefficient FROH, the Runs of Homozygosity distribution per chromosome and classes of length as well as the total amount of the genome covered by these segments, were used to assess the level of inbreeding of the NW-EU populations and to infer past demographic events. All the calculations were performed with the R package DetectRUNS 0.9.5^52^ (R core v4.1) applying the consecutive function and with the following settings: minSNP = 20, maxGap = 10^6, minLengthBps = 1 Mb, maxOppRun = 1, maxMissRun = 1. The five classes of length are the following: 0–2, 2–4, 4–8, 8–16 and > 16 Mb.

### Phylogeographic inference and admixture events

To assess reticulate relationships between the study breeds, the matrix of Reynolds’ unweighted distances previously calculated was used to infer and visualise the Neighbor-Net via the software SplitsTree^53^ v4.14.2. The program TreeMix v1.13^14^ was applied to infer the gene flow and admixture events that occurred in the past. By using allele frequencies of SNP, we calculated a Maximum Likelihood tree that represents the evolutionary relationships across the analysed populations. We ran the program until the residuals plot R^2^ reached at least 0.998, that is from 0 (no migration) to 11 for the NW-EU dataset and from 0 to 15 for the WHOLE dataset, using the bezoar outgroup. Finally, we plotted the results by means of the plotting_funcs.R script provided within the same program. We also applied the function “treemix.fit” in BITE^44^ to evaluate TreeMix results, reporting the first population encountered in the donor subgraph, the first population encountered in the receiving subgraph, the migration weight (i.e. the estimated fraction of ancestry) in the receiving subgraph derived from the donor portion, jackknife and standard error estimate, and the p-value associated to the weight. As the authors of the program specified^14^, a “significant” p-value simply indicates that the hypothesised migration event significantly improves the fit to the data; this does not exclude the possibility of errors or indicate that the particular migration event tested is the correct one. For this reason, we used additional methods like three- and four-population (f3 and f4) statistics^14^ to test the robustness of the inference. These statistics were inferred with ADMIXTOOLS v5.1^54^ and standard errors (SE) were computed with the block-Jackknife procedure using default options and Z-scores calculated on the SE. We tested for all the possible combinations of trees from the WHOLE dataset, producing a total of 6,900 triplets for thef3 test and 37,950 possible tree topologies for the f4 test. Afterwards, we checked all the results and retained only the most significant ones (− 20 < Z-score >  + 20). Thus, we focused only on the NW-EU target breeds, screening the results that had a minimum of − 4 < Z-score >  + 4 (p-value < 1 × 10 − 6).

### Proportion of introgression and past gene flow estimates

To confirm the signal of gene flow found with the above mentioned methods, we applied f4-ratio estimation α, in ADMIXTOOLS v5.1^54^, which allows the inference of the admixture proportions without access to accurate surrogates for the ancestral populations. The proportion of ancestry was computed as:

1 $$\alpha =\frac{f4(A, O;X, C)}{f4(A, O;B,C)},$$

in which O is the outgroup (BEZ), B a first reference population, C the other possible reference population, A a population related to B, and X the target population. We tested only the breeds that could corroborate the TreeMix migration events and f4 statistics.

The time of admixture was estimated with ALDER v.1.05^55^ using the default parameters with a minimum genetic distance pre-calculated by the program and the 'maxchrom' parameter (set to 22 by default) that enables to use more than 22 chromosomes (maxchrom = 29). ALDER uses genotype data from a test population C and two reference populations A and B and computes unbiased weighted linkage disequilibrium (LD) statistic for making inference about admixture events in the target population C. If population C is derived from an admixture between two source populations close to the reference populations A and B, the pairwise LD of the three curves computed with A-B, A-C, and B-C weights should all exhibit a statistically significant signal of exponential decay as a function of the genetic distance. Thus, ALDER compares the three weighted LD curves to test for admixture and infers the decay rates that correspond to age of admixture. The p-value is also provided in the output from standard errors estimated by jackknifing over chromosomes. We performed the analysis on the basis of the results of the statistics described above (TreeMix, f4-ratio) and joining the historical information with the most recent human genetic studies about Scandinavian and Celtic people^29, 31^.

### Supplementary Information

Supplementary Figures.

Supplementary Figure 3.

Supplementary Figure 4.

Supplementary Figure 5.

Supplementary Figure 5.

Supplementary Figure 6.

Supplementary Table S1.

Supplementary Table S2.

Supplementary Table S3.

Supplementary Table S4.

Supplementary Table S5.

Supplementary Table S6.

Supplementary Table S6.

Supplementary Table S7.

Supplementary Table S8.

## Data Availability

The datasets analysed during the current study is available in the Dryad Repository (https://doi.org/10.5061/dryad.v8g21pt), on Mendeley Data (https://doi.org/10.17632/hnd59x6gmg.1; URL: https://data.mendeley.com/datasets/hnd59x6gmg/1), from NIBIO The Norwegian Genetic Resource Centre under request and from Smarter project which database will be available at the end of the project and once the manuscript is published.

## References

[1] Amills M, Capote J, Tosser-Klopp G (2017). Goat domestication and breeding: A jigsaw of historical, biological and molecular data with missing pieces. Anim. Genet..

[2] Ferrando A (2015). A mitochondrial analysis reveals distinct founder effect signatures in Canarian and Balearic goats. Anim. Genet..

[3] Zheng Z (2020). The origin of domestication genes in goats. Sci. Adv..

[4] Macheridis S (2022). Animal husbandry in Iron Age Scania, with a catalogue. Acta Archaeol. Lundensia Ser. Altera.

[5] Cassidy LM (2017). Capturing goats: Documenting two hundred years of mitochondrial DNA diversity among goat populations from Britain and Ireland. Biol. Lett..

[6] Pogorevc N (2021). Post-genotyping optimization of dataset formation could affect genetic diversity parameters: An example of analyses with alpine goat breeds. BMC Genom..

[7] Larson G, Fuller DQ (2014). The evolution of animal domestication. Annu. Rev. Ecol. Evol. Syst..

[8] Colli L (2018). Genome-wide SNP profiling of worldwide goat populations reveals strong partitioning of diversity and highlights post-domestication migration routes. Genet. Sel. Evol..

[9] Bertolini F (2018). Genome-wide patterns of homozygosity provide clues about the population history and adaptation of goats. Genet. Sel. Evol..

[10] Cardoso TF (2018). Patterns of homozygosity in insular and continental goat breeds. Genet. Sel. Evol..

[11] Alberto FJ (2018). Convergent genomic signatures of domestication in sheep and goats. Nat. Commun..

[12] Gabriel SI, Mathias ML, Searle JB (2015). Of mice and the ‘Age of Discovery’: The complex history of colonization of the Azorean archipelago by the house mouse (Mus musculus) as revealed by mitochondrial DNA variation. J. Evol. Biol..

[13] Rando JC, Pieper H, Alcover JA (2014). Radiocarbon evidence for the presence of mice on Madeira Island (North Atlantic) one millennium ago. Proc. R. Soc. B Biol. Sci..

[14] Pickrell JK, Pritchard JK (2012). Inference of population splits and mixtures from genome-wide allele frequency data. PLoS Genet..

[15] Lipson M (2020). Applying f4-statistics and admixture graphs: Theory and examples. Mol. Ecol. Resour..

[16] Peter BM (2016). Admixture, population structure, and f-statistics. Genetics.

[17] Ceballos FC, Joshi PK, Clark DW, Ramsay M, Wilson JF (2018). Runs of homozygosity: Windows into population history and trait architecture. Nat. Rev. Genet..

[18] Price O. The Bilberry goats. *Irish Wildl. Trust. Fles. Nat.* https://bilberrygoats.files.wordpress.com/2006/12/bilberry-fact-filefina2.pdf. Accessed 03 May 2018 (2006).

[19] Manunza A (2016). A genome-wide perspective about the diversity and demographic history of seven Spanish goat breeds. Genet. Sel. Evol..

[20] Cassidy LM (2016). Neolithic and bronze age migration to Ireland and establishment of the insular atlantic genome. Proc. Natl. Acad. Sci. U.S.A..

[21] Cassidy LM (2020). A dynastic elite in monumental Neolithic society. Nature.

[22] Fages A (2019). Tracking five millennia of horse management with extensive ancient genome time series. Cell.

[23] Olalde I (2019). The genomic history of the Iberian Peninsula over the past 8000 years. Science.

[24] Sveinsdóttir HE, Dýrmundsson ÓR (1994). The Icelandic goat breed. Icel. Agric. Sci..

[25] Baldursdottir BK, Kristjansson T, Hallsson JH (2012). Diversity of the Icelandic goat breed assessed using population data. Acta Agric. Scand. A Anim. Sci..

[26] Sunna Ebenesersdóttir S (2018). Ancient genomes from Iceland reveal the making of a human population. Science.

[27] Tapio M (2005). Native breeds demonstrate high contributions to the molecular variation in northern European sheep. Mol. Ecol..

[28] Porter V, Alderson L, Hall SJG, Sponenberg DP (2016). Masons World Encyclopedia of Livestock Breeds and Breeding.

[29] Byrne RP (2018). Insular celtic population structure and genomic footprints of migration. PLoS Genet..

[30] MacHugh DE (1999). Early medieval cattle remains from a Scandinavian settlement in Dublin: Genetic analysis and comparison with extant breeds. Philos. Trans. R. Soc. B Biol. Sci..

[31] Rodríguez-Varela R (2023). The genetic history of Scandinavia from the Roman Iron Age to the present. Cell.

[32] Frei KM, Mannering U, Vanden Berghe I, Kristiansen K (2017). Bronze Age wool: Provenance and dye investigations of Danish textiles. Antiquity.

[33] Löffelmann T (2023). Sr analyses from only known Scandinavian cremation cemetery in Britain illuminate early Viking journey with horse and dog across the North Sea. PLoS One.

[34] Bowden GR (2008). Excavating past population structures by surname-based sampling: The genetic legacy of the vikings in Northwest England. Mol. Biol. Evol..

[35] Stella A (2018). AdaptMap: Exploring goat diversity and adaptation. Genet. Sel. Evol..

[36] Burren OS (2020). Genetic feature engineering enables characterisation of shared risk factors in immune-mediated diseases. Genome Med..

[37] Berg P (2020). Genetic characterization of a small closed island population of Norwegian coastal goat. Acta Agric. Scand. Sect. A Anim. Sci..

[38] Denoyelle L, Talouarn E, Bardou P (2021). VarGoats project: A dataset of 1159 whole-genome sequences to dissect Capra hircus global diversity. Genet. Sel. Evol..

[39] Tosser-Klopp G (2014). Design and characterization of a 52K SNP chip for goats. PLoS One.

[40] Andersson E (2019). Böldsjuka och Kaprin Artrit Encefalit Hos Svenska Mjölkproducerande Getter: En Prevalensstudie Och Jämförelse Av Serum Och Mjölk Som Provtagningsmaterial.

[41] Chang CC (2015). Second-generation PLINK: Rising to the challenge of larger and richer datasets. Gigascience.

[42] Ajmone-Marsan P, Boettcher PJ, Ginja C, Kantanen J, Lenstra JA (2023). Genomic Characterization of Animal Genetic Resources Practical guide.

[43] Lachance J, Tishkoff SA (2013). SNP ascertainment bias in population genetic analyses: Why it is important, and how to correct it. Bioessays.

[44] Milanesi M, Capomaccio S, Vajana E, Bomba L, Garcia JF, Ajmone Marsan P, Colli L (2017). BITE: An R package for biodiversity analyses. bioRxiv.

[45] Lischer HEL, Excoffier L (2012). PGDSpider: An automated data conversion tool for connecting population genetics and genomics programs. Bioinformatics.

[46] Excoffier L, Lischer HEL (2010). Arlequin suite ver 3.5: A new series of programs to perform population genetics analyses under Linux and Windows. Mol. Ecol. Resour..

[47] Price AL (2006). Principal components analysis corrects for stratification in genome-wide association studies. Nat. Genet..

[48] Patterson N, Price AL, Reich D (2006). Population structure and eigenanalysis. PLoS Genet..

[49] Zheng X (2012). A high-performance computing toolset for relatedness and principal component analysis of SNP data. Bioinformatics.

[50] Alexander DH, Novembre J, Lange K (2009). Fast model-based estimation of ancestry in unrelated individuals. Genome Res..

[51] Barbato M, Orozco-terWengel P, Tapio M, Bruford MW (2015). SNeP: A tool to estimate trends in recent effective population size trajectories using genome-wide SNP data. Front. Genet..

[52] Biscarini, F., Cozzi, P., Gaspa, G. & Marras, G. DetectRUNS: detect runs of homozygosity and runs of heterozygosity in diploid genomes. R package Version 0.9.5 (2018).

[53] Huson DH (1998). SplitsTree: Analyzing and visualizing evolutionary data. Bioinformatics.

[54] Patterson N (2012). Ancient admixture in human history. Genetics.

[55] Loh PR (2013). Inferring admixture histories of human populations using linkage disequilibrium. Genetics.

