# Supplementary material for: Genetic diversity and historical demography of underutilised goat breeds in North-Western Europe
Source: Sci Rep. 2023 Nov 25;13:20728. doi: 10.1038/s41598-023-48005-8 (PMC10676416; doi:10.1038/s41598-023-48005-8)
Supplement: Supplementary file 1 — Supplementary Figures. [file 41598_2023_48005_MOESM1_ESM.docx]

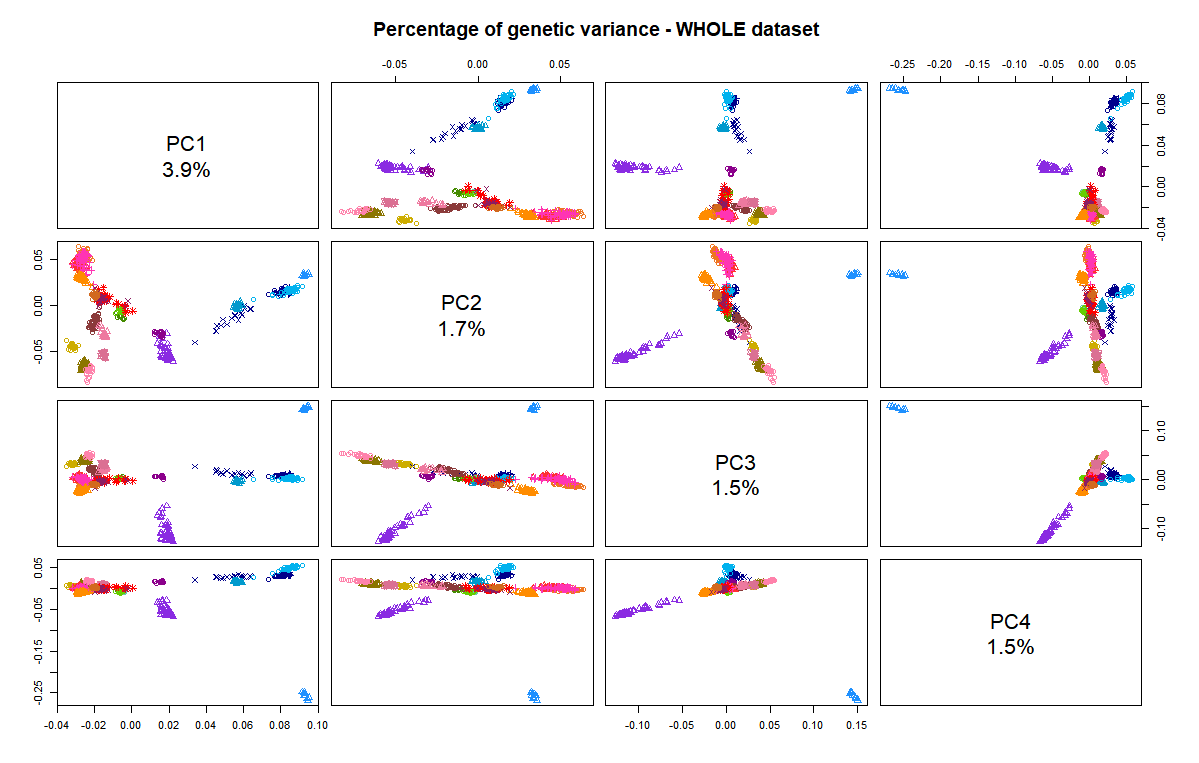


**A**


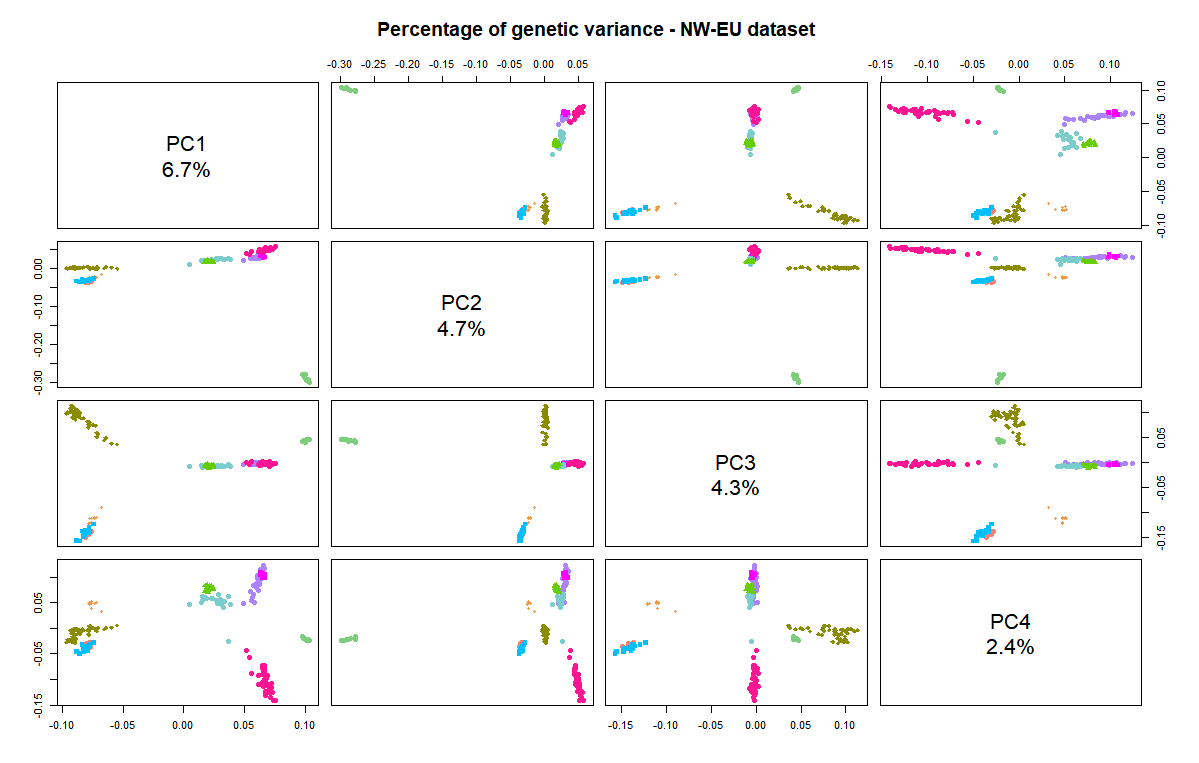


**B**

Supplementary Figures 1 A-B. Total amount of the genetic variance in percentage accounted for the first four PCs. A) PCA and variance for the WHOLE dataset; B) PCA and variance for the target NW-EU dataset.
