# Supplementary material for: Genetic diversity and historical demography of underutilised goat breeds in North-Western Europe
Source: Sci Rep. 2023 Nov 25;13:20728. doi: 10.1038/s41598-023-48005-8 (PMC10676416; doi:10.1038/s41598-023-48005-8)
Supplement: Supplementary file 2 — Supplementary Figure 3. [file 41598_2023_48005_MOESM2_ESM.docx]

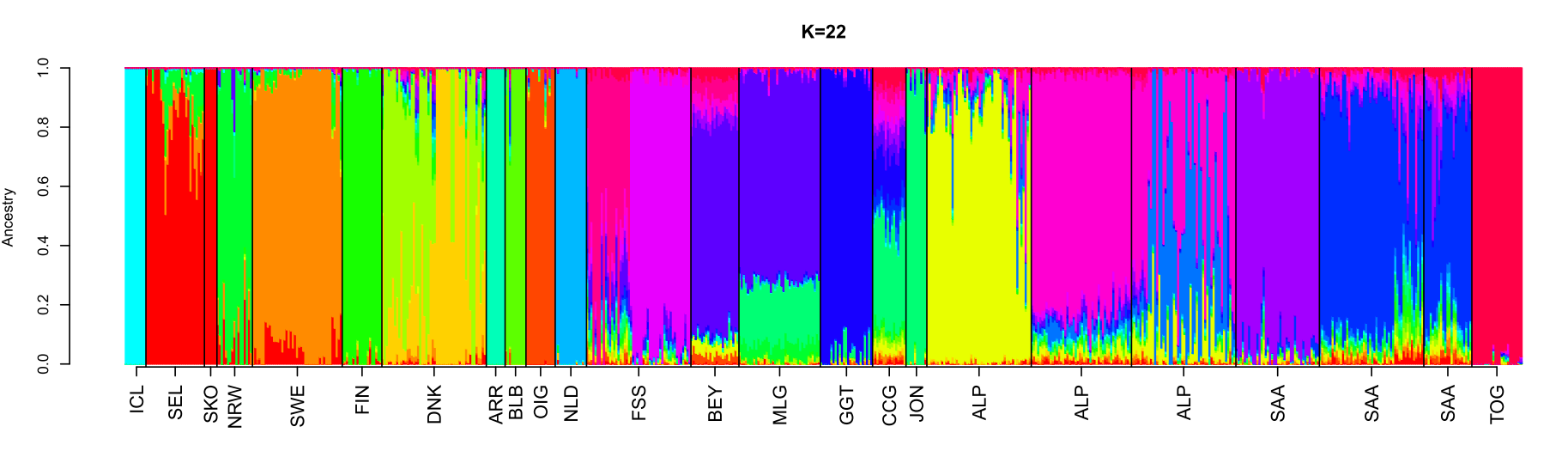


**A**


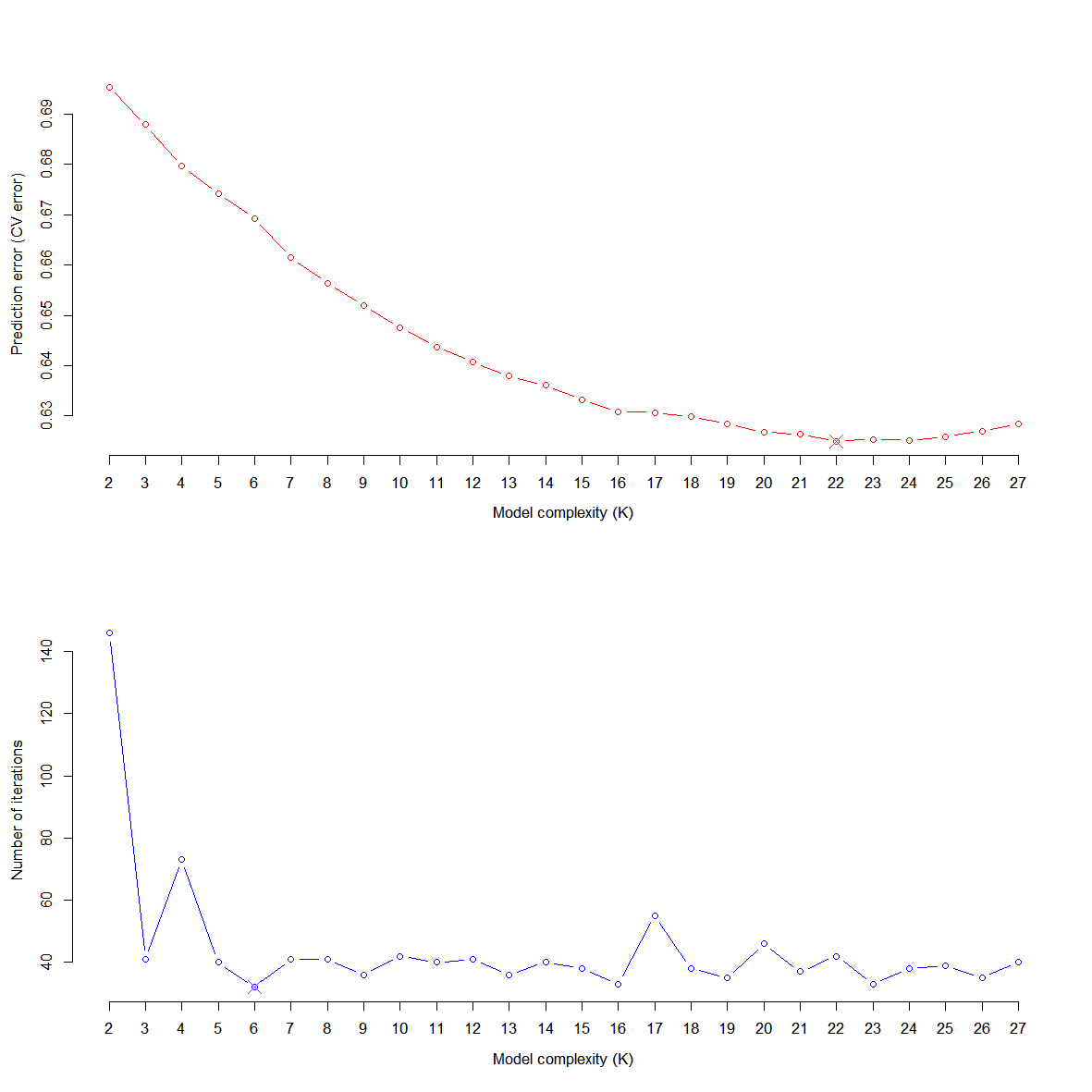


**B**

Supplementary Figures 3 A-B. A) The most probable number of K (genetic components) is 22. The Alpine populations are ALP_CH, ALP_IT and ALP_FR, and Saanen are SAA_CH, SAA_FR and SAA_IT, from left to right. B) Cross-validation plot for the unsupervised clustering method of ADMIXTURE.
