# Supplementary material for: Genetic diversity and historical demography of underutilised goat breeds in North-Western Europe
Source: Sci Rep. 2023 Nov 25;13:20728. doi: 10.1038/s41598-023-48005-8 (PMC10676416; doi:10.1038/s41598-023-48005-8)
Supplement: Supplementary file 3 — Supplementary Figure 4. [file 41598_2023_48005_MOESM3_ESM.docx]

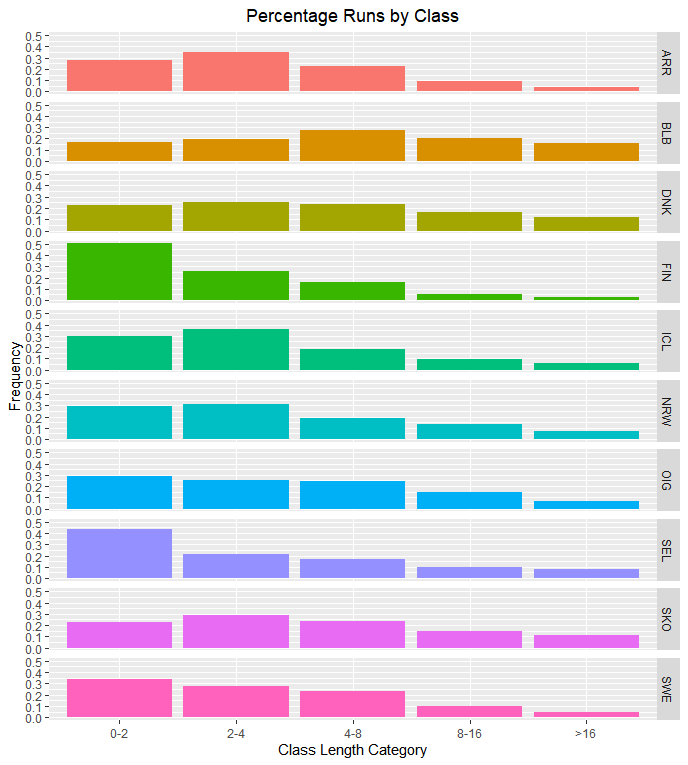


**A**


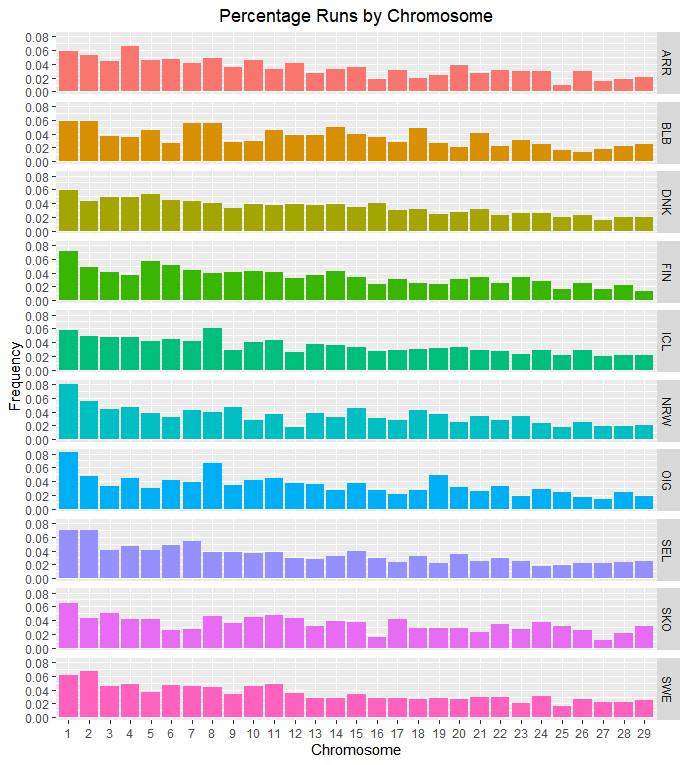


**B**


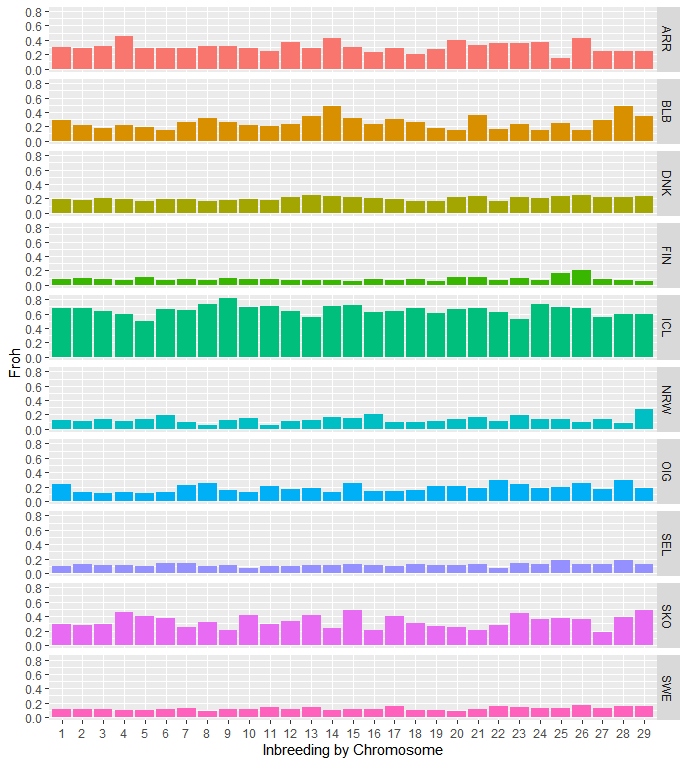


**C**

Supplementary Figures 4 A-C. Genomic patterns of homozygosity in the target breeds. A) Distribution of ROH per classes of length (the five ROH classes under consideration); B) Percentage of ROH per chromosome and C) genomic inbreeding coefficient (FROH) per chromosome.
