# Supplementary material for: Genetic diversity and historical demography of underutilised goat breeds in North-Western Europe
Source: Sci Rep. 2023 Nov 25;13:20728. doi: 10.1038/s41598-023-48005-8 (PMC10676416; doi:10.1038/s41598-023-48005-8)
Supplement: Supplementary file 4 — Supplementary Figure 5. [file 41598_2023_48005_MOESM4_ESM.pdf]

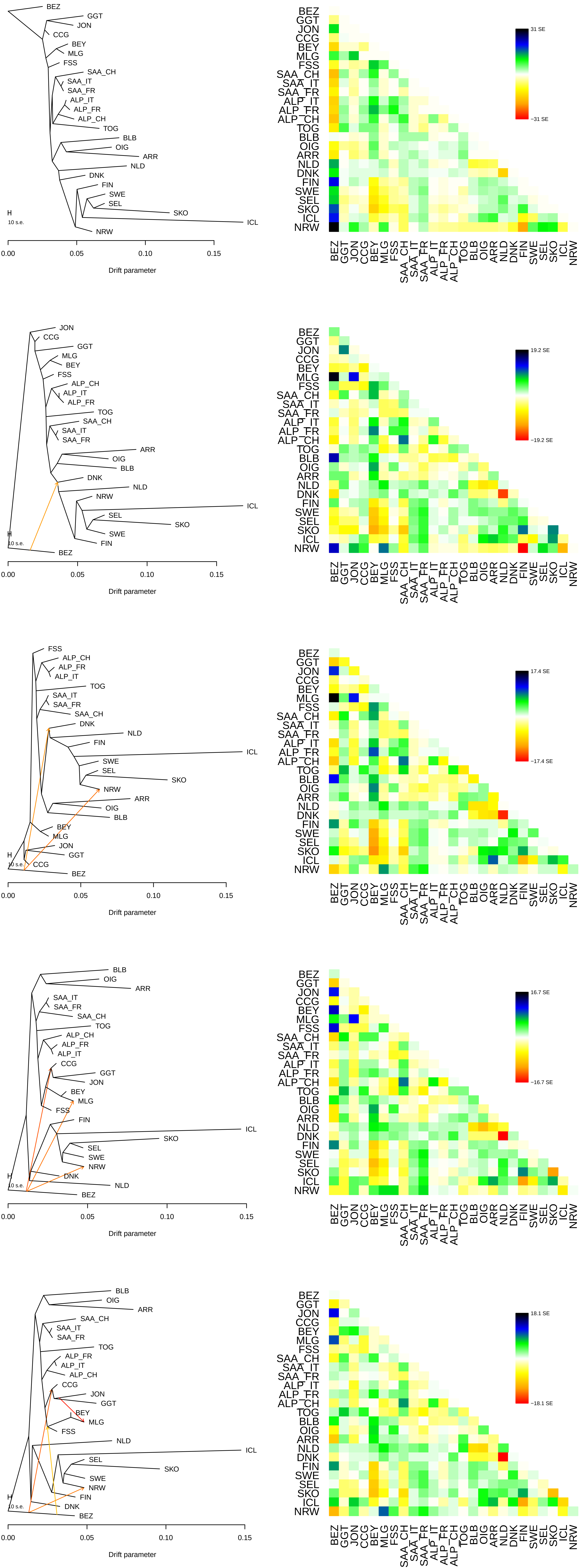

BEZ
GGT
JON
CCG
BEY
MLG
FSS
SAA_CH
SAA_IT
SAA_FR
ALP_IT
ALP_FR
ALP_CH
TOG
BLB
OIG
ARR
NLD
DNK
FIN
SWE
SEL
SKO
ICL
NRW
10 s.e.
0.00
0.05
0.10
0.15
Drift parameter
31 SE
−31 SE
19.2 SE
−19.2 SE
17.4 SE
−17.4 SE
16.7 SE
−16.7 SE
18.1 SE
−18.1 SE

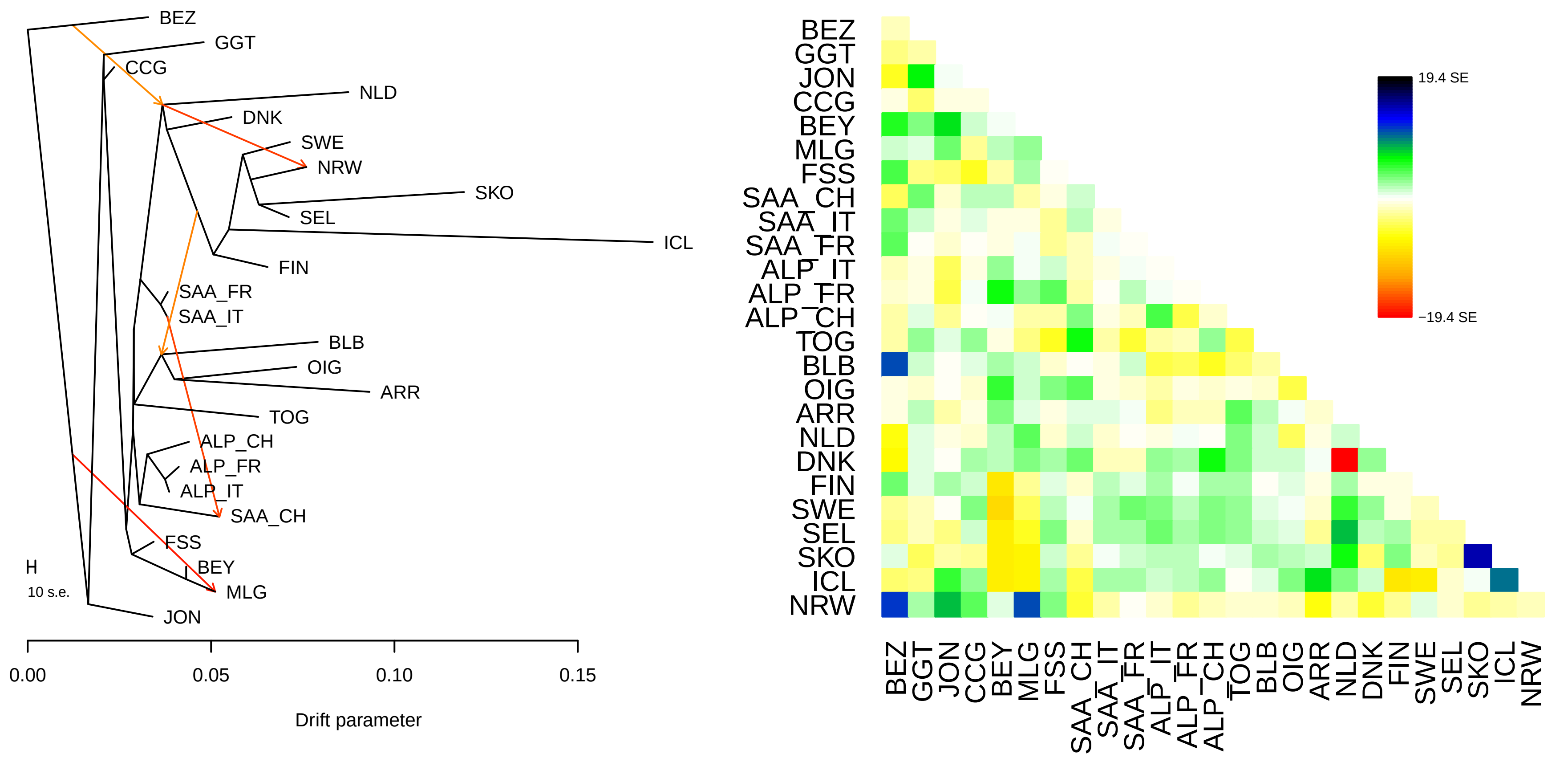
BEZ GGT CCG NLD DNK SWE NRW SKO SEL ICL FIN SAA_FR SAA_IT BLB OIG ARR TOG ALP_CH ALP_FR ALP_IT SAA_CH FSS BEY MLG JON
10 s.e.
0.00 0.05 0.10 0.15
Drift parameter
19.4 SE
−19.4 SE
BEZ GGT JON CCG BEY MLG FSS SAA_CH SAA_IT SAA_FR ALP_IT ALP_FR ALP_CH TOG BLB OIG ARR NLD DNK FIN SWE SEL SKO ICL NRW

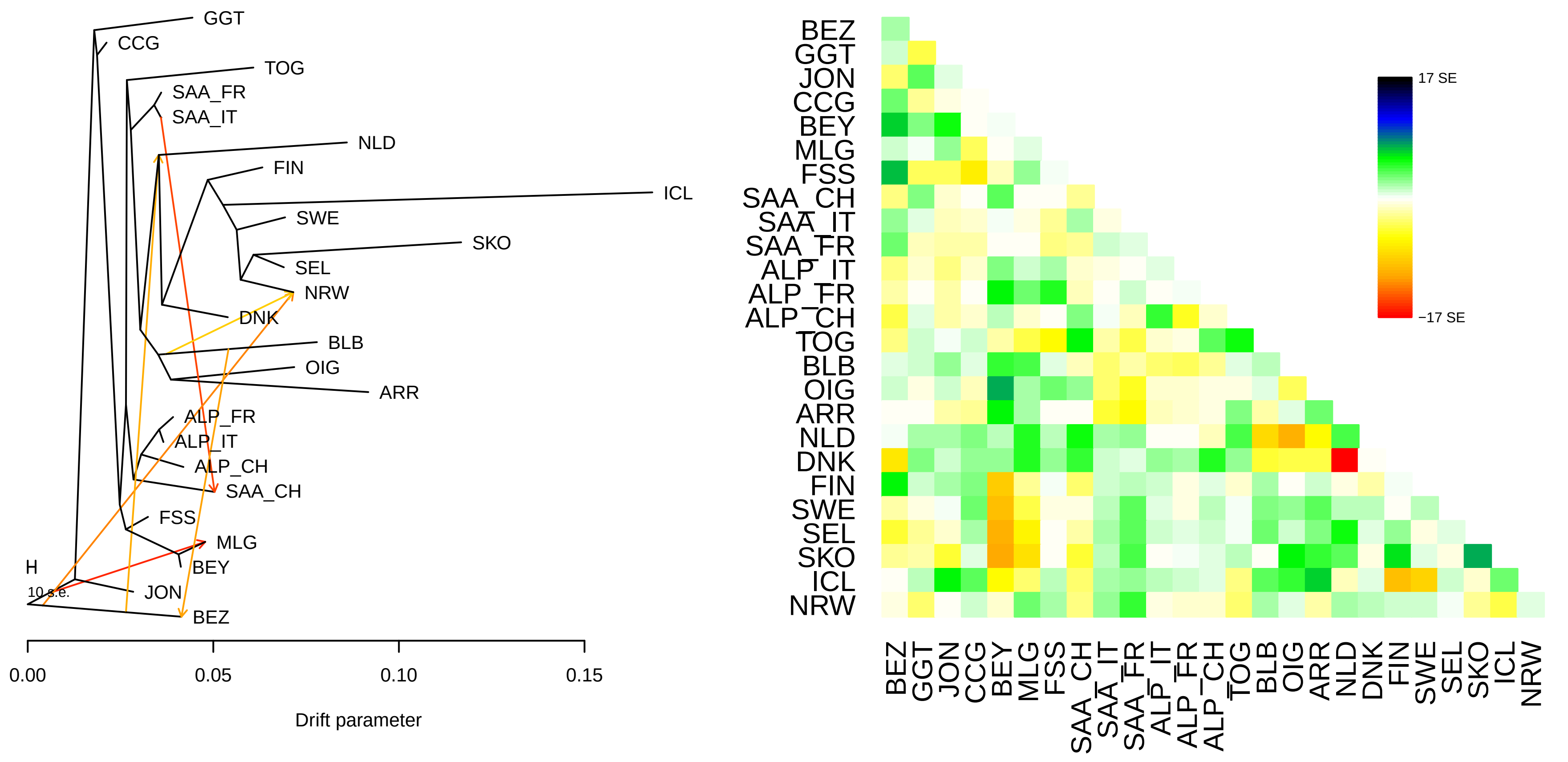
GGT CCG TOG SAA_FR SAA_IT NLD FIN ICL SWE SKO SEL NRW DNK BLB OIG ARR ALP_FR ALP_IT ALP_CH SAA_CH FSS MLG BEY JON BEZ
10 s.e.
0.00 0.05 0.10 0.15
Drift parameter
17 SE
−17 SE
BEZ GGT JON CCG BEY MLG FSS SAA_CH SAA_IT SAA_FR ALP_IT ALP_FR ALP_CH TOG BLB OIG ARR NLD DNK FIN SWE SEL SKO ICL NRW

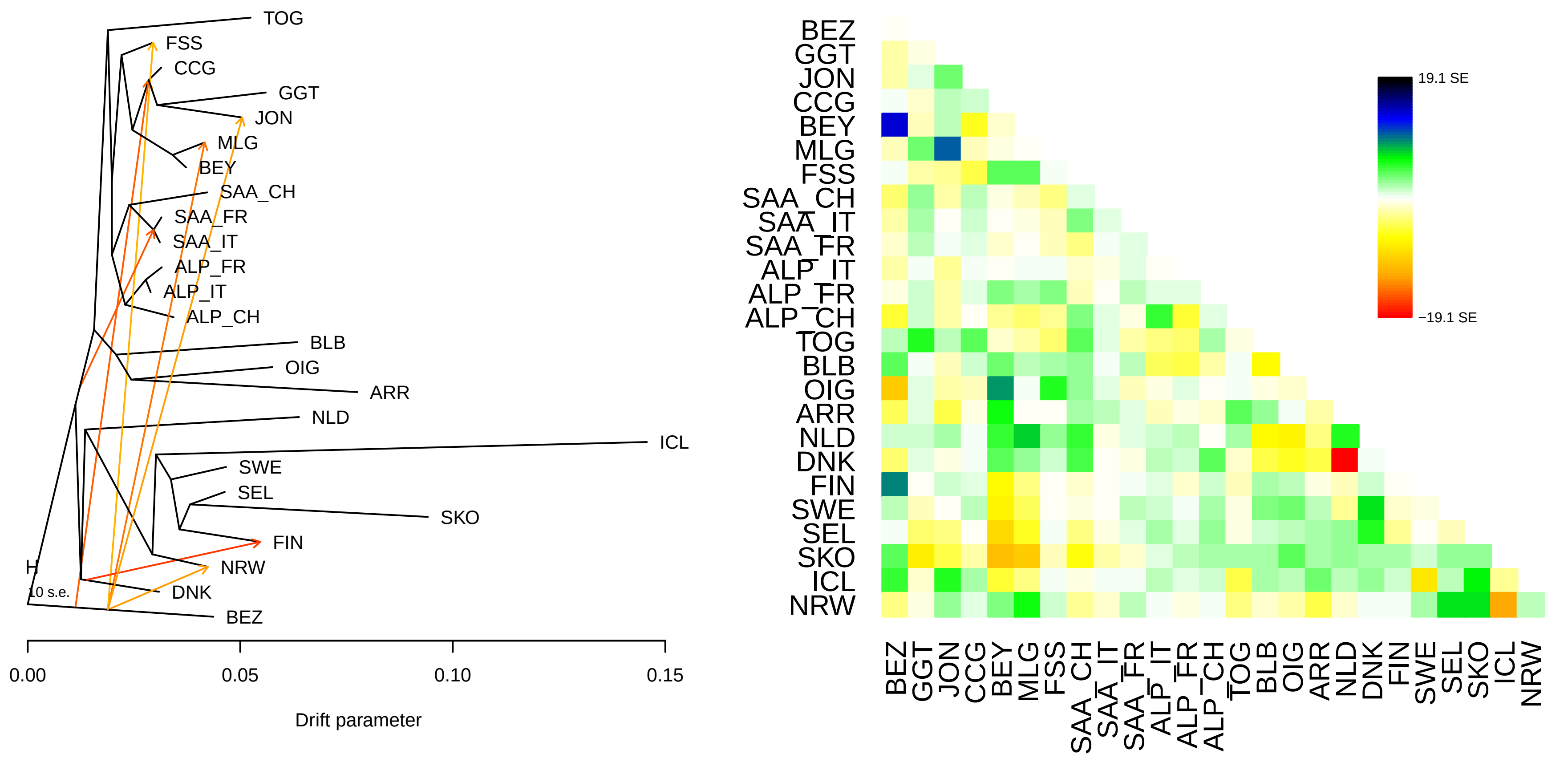
TOG FSS CCG GGT JON MLG BEY SAA_CH SAA_FR SAA_IT ALP_FR ALP_IT ALP_CH BLB OIG ARR NLD ICL SWE SEL SKO FIN NRW DNK BEZ
10 s.e.
0.00 0.05 0.10 0.15
Drift parameter
19.1 SE
−19.1 SE
BEZ GGT JON CCG BEY MLG FSS SAA_CH SAA_IT SAA_FR ALP_IT ALP_FR ALP_CH TOG BLB OIG ARR NLD DNK FIN SWE SEL SKO ICL NRW

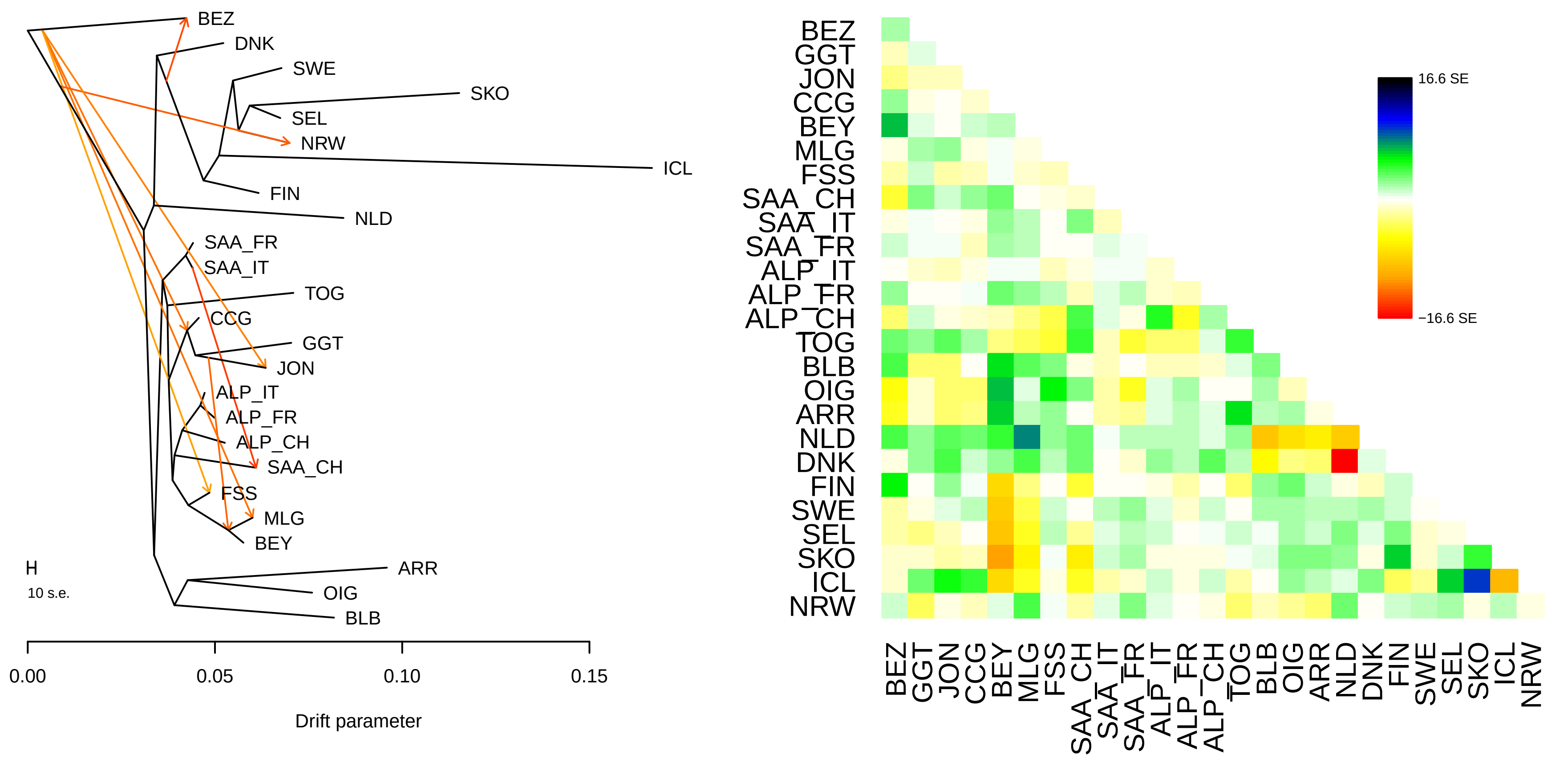
BEZ DNK SWE SKO SEL NRW ICL FIN NLD SAA_FR SAA_IT TOG CCG GGT JON ALP_IT ALP_FR ALP_CH SAA_CH FSS MLG BEY ARR OIG BLB
10 s.e.
0.00 0.05 0.10 0.15
Drift parameter
16.6 SE
−16.6 SE
BEZ GGT JON CCG BEY MLG FSS SAA_CH SAA_IT SAA_FR ALP_IT ALP_FR ALP_CH TOG BLB OIG ARR NLD DNK FIN SWE SEL SKO ICL NRW

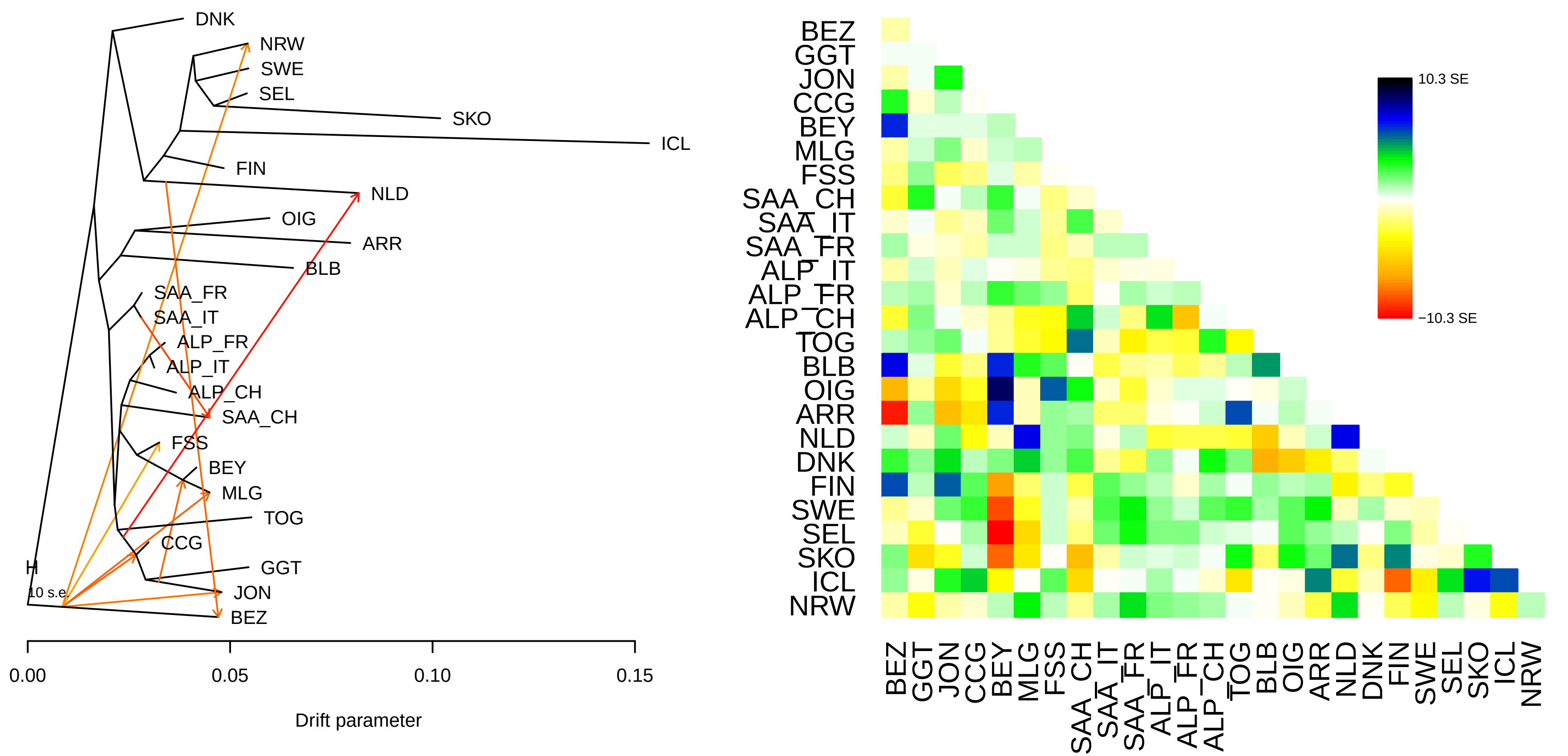
DNK NRW SWE SEL SKO ICL FIN NLD OIG ARR BLB SAA_FR SAA_IT ALP_FR ALP_IT ALP_CH SAA_CH FSS BEY MLG TOG CCG GGT JON BEZ
10 s.e.
0.00 0.05 0.10 0.15
Drift parameter
10.3 SE
−10.3 SE
BEZ GGT JON CCG BEY MLG FSS SAA_CH SAA_IT SAA_FR ALP_IT ALP_FR ALP_CH TOG BLB OIG ARR NLD DNK FIN SWE SEL SKO ICL NRW

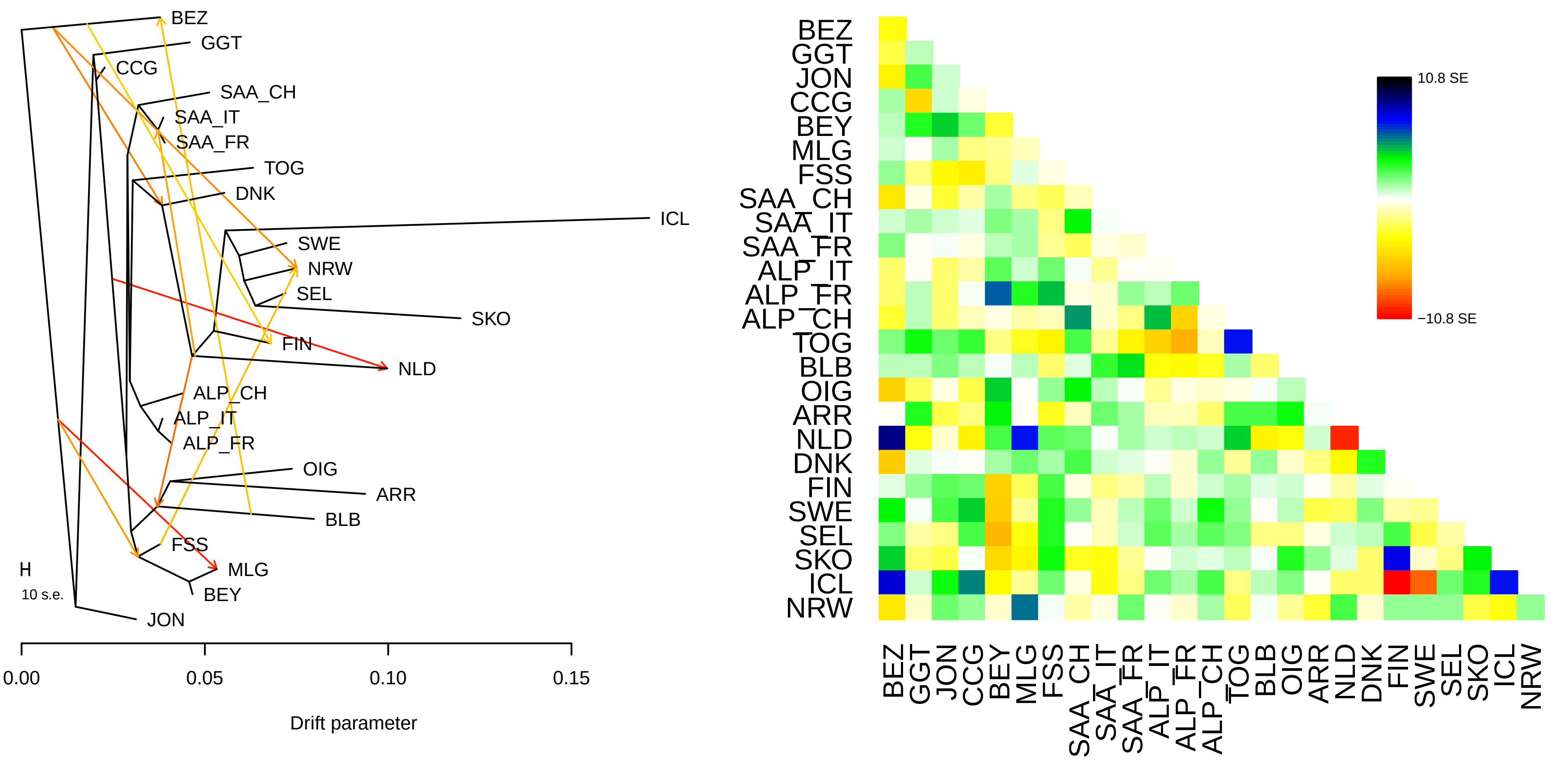
BEZ
GGT
CCG
SAA_CH
SAA_IT
SAA_FR
TOG
DNK
ICL
SWE
NRW
SEL
SKO
FIN
NLD
ALP_CH
ALP_IT
ALP_FR
OIG
ARR
BLB
FSS
MLG
BEY
JON
H
10 s.e.
0.00
0.05
0.10
0.15
Drift parameter
10.8 SE
−10.8 SE

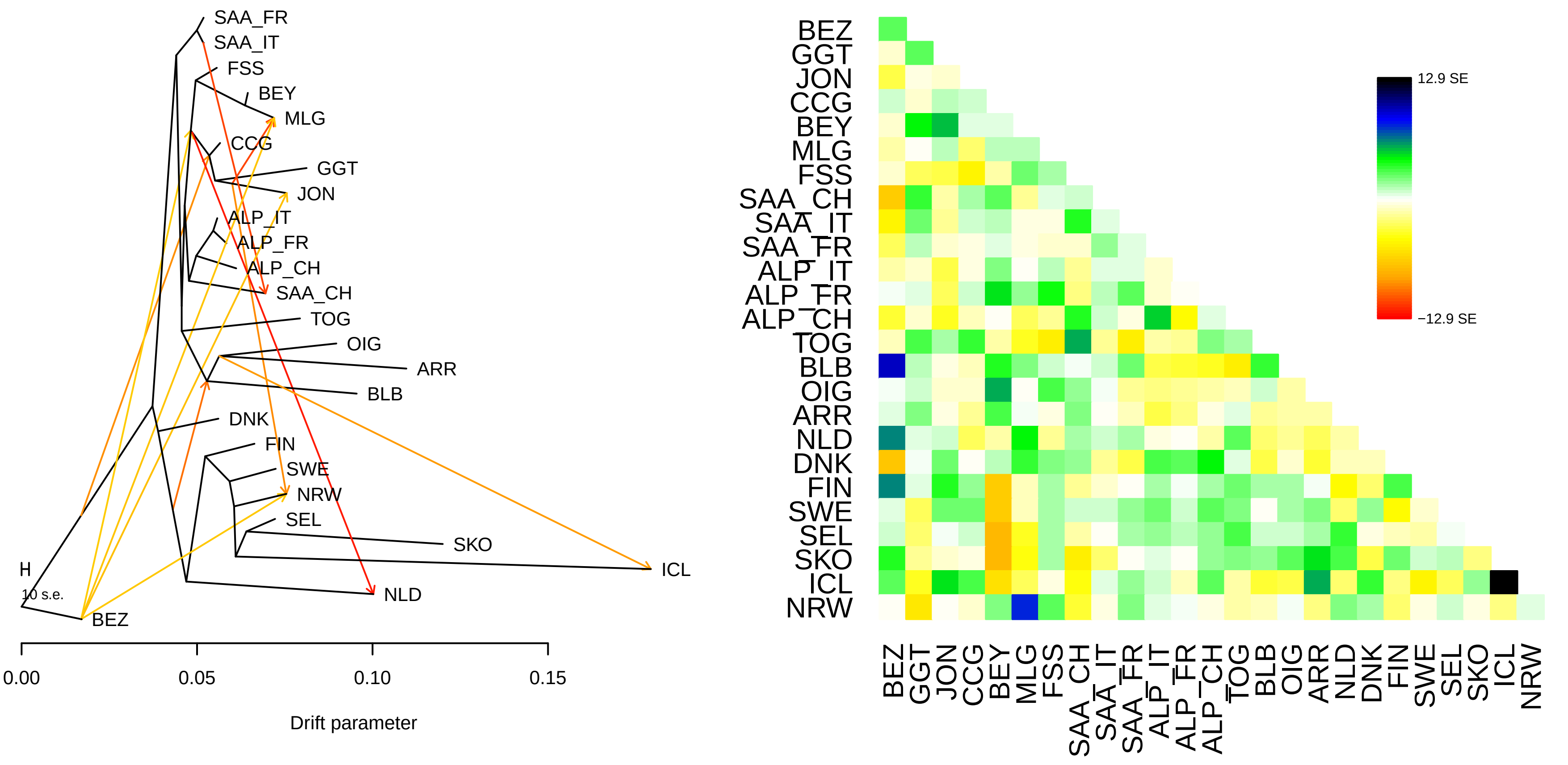
SAA_FR
SAA_IT
FSS
BEY
MLG
CCG
GGT
JON
ALP_IT
ALP_FR
ALP_CH
SAA_CH
TOG
OIG
ARR
BLB
DNK
FIN
SWE
NRW
SEL
SKO
ICL
NLD
BEZ
H
10 s.e.
0.00
0.05
0.10
0.15
Drift parameter
12.9 SE
−12.9 SE

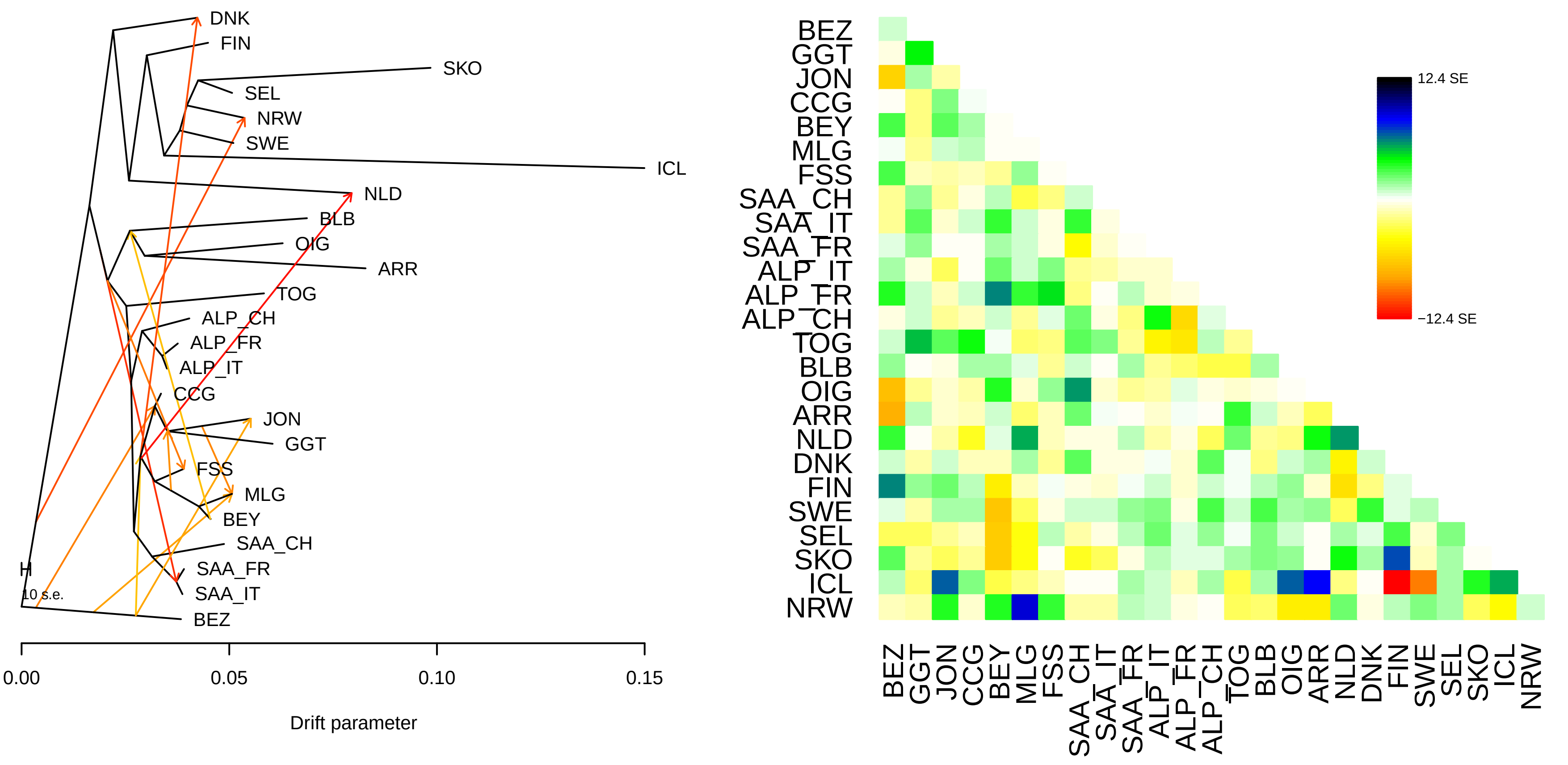
DNK
FIN
SKO
SEL
NRW
SWE
ICL
NLD
BLB
OIG
ARR
TOG
ALP_CH
ALP_FR
ALP_IT
CCG
JON
GGT
FSS
MLG
BEY
SAA_CH
SAA_FR
SAA_IT
BEZ
H
10 s.e.
0.00
0.05
0.10
0.15
Drift parameter
12.4 SE
−12.4 SE

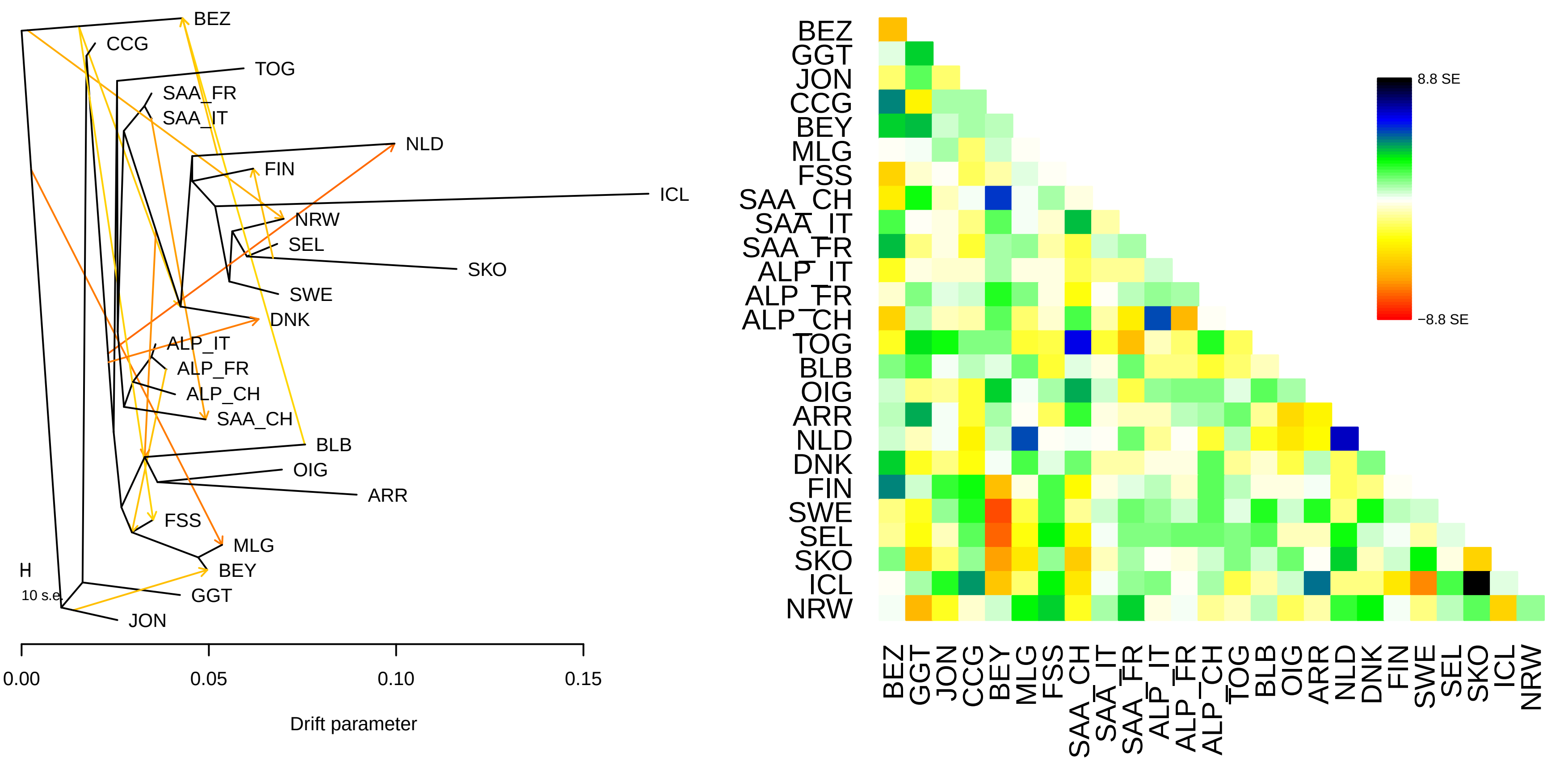
BEZ
CCG
TOG
SAA_FR
SAA_IT
NLD
FIN
ICL
NRW
SEL
SKO
SWE
DNK
ALP_IT
ALP_FR
ALP_CH
SAA_CH
BLB
OIG
ARR
FSS
MLG
BEY
GGT
JON
H
10 s.e.
0.00
0.05
0.10
0.15
Drift parameter
8.8 SE
−8.8 SE

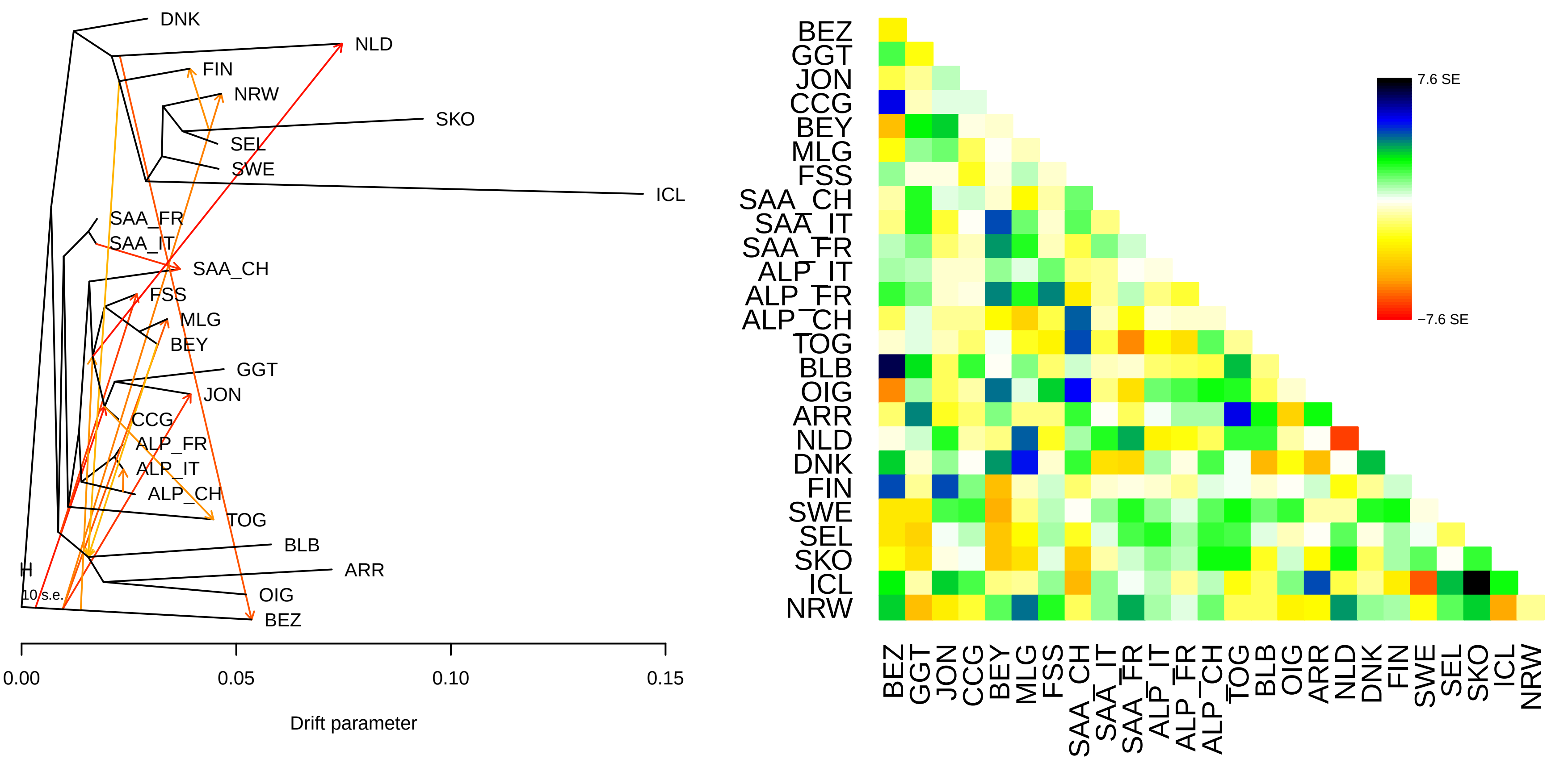
DNK
NLD
FIN
NRW
SKO
SEL
SWE
ICL
SAA_FR
SAA_IT
SAA_CH
FSS
MLG
BEY
GGT
JON
CCG
ALP_FR
ALP_IT
ALP_CH
TOG
BLB
ARR
OIG
BEZ
H
10 s.e.
0.00
0.05
0.10
0.15
Drift parameter
7.6 SE
−7.6 SE

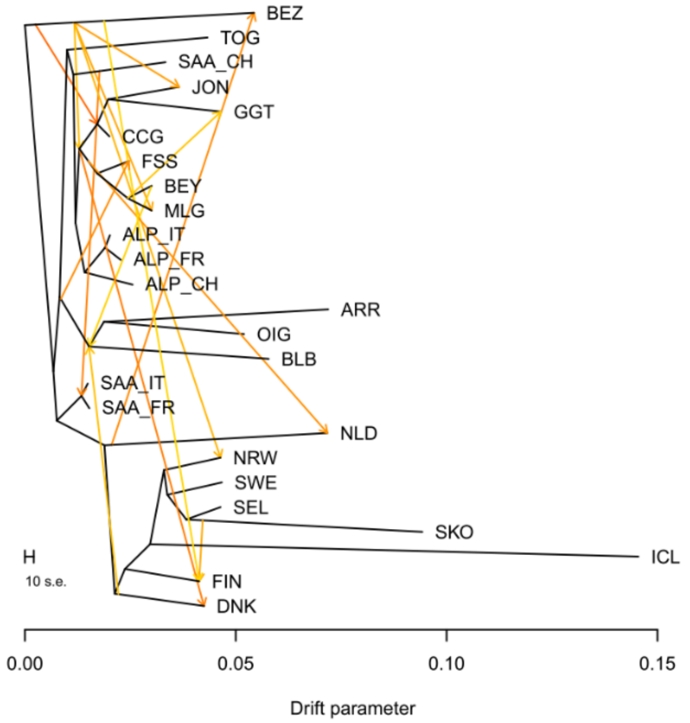
BEZ
TOG
SAA_CH
JON
GGT
CCG
FSS
BEY
MLG
ALP_IT
ALP_FR
ALP_CH
ARR
OIG
BLB
SAA_IT
SAA_FR
NLD
NRW
SWE
SEL
SKO
ICL
FIN
DNK
H
10 s.e.
0.00
0.05
0.10
0.15
Drift parameter

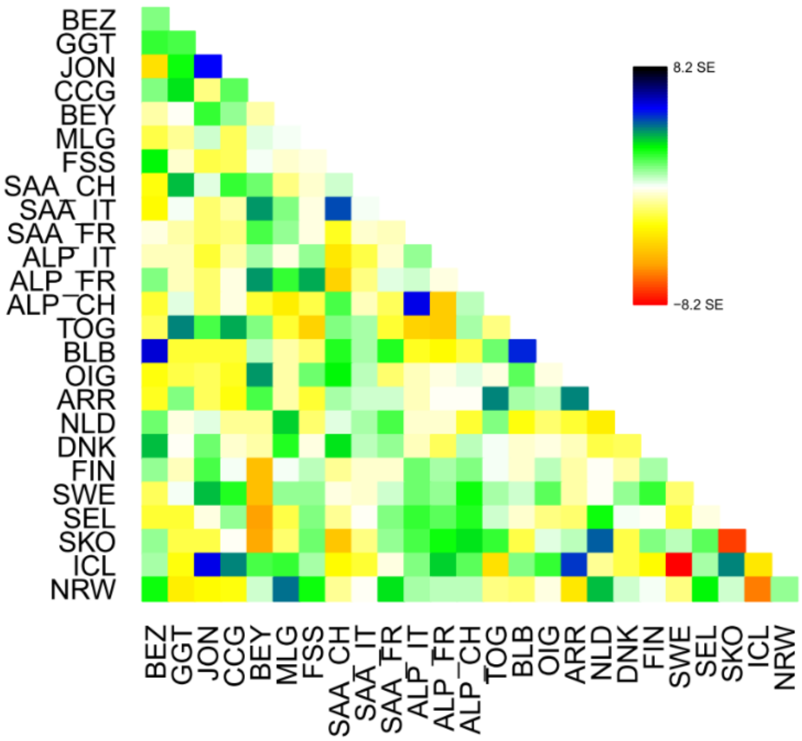
BEZ
GGT
JON
CCG
BEY
MLG
FSS
SAA_CH
SAA_IT
SAA_FR
ALP_IT
ALP_FR
ALP_CH
TOG
BLB
OIG
ARR
NLD
DNK
FIN
SWE
SEL
SKO
ICL
NRW
8.2 SE
−8.2 SE

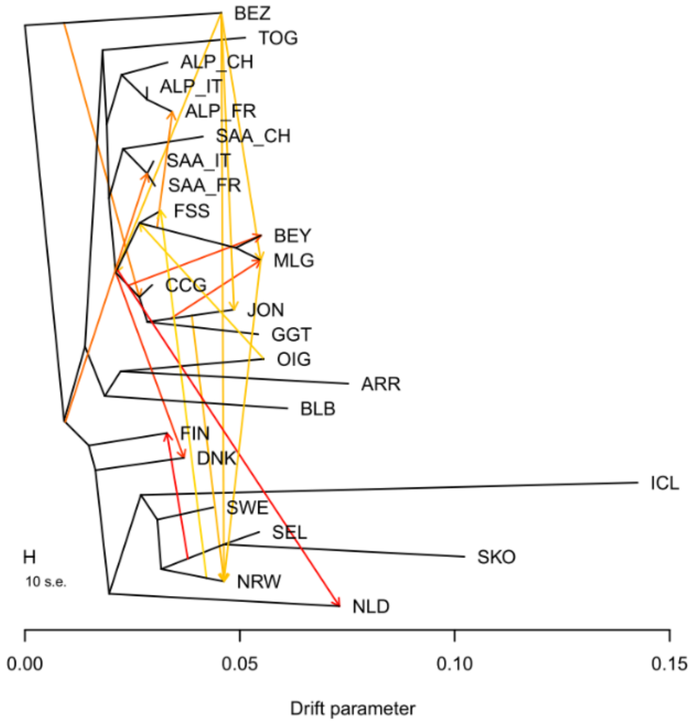
BEZ
TOG
ALP_CH
ALP_IT
ALP_FR
SAA_CH
SAA_IT
SAA_FR
FSS
BEY
MLG
CCG
JON
GGT
OIG
ARR
BLB
FIN
DNK
ICL
SWE
SEL
SKO
NRW
NLD
H
10 s.e.
0.00
0.05
0.10
0.15
Drift parameter

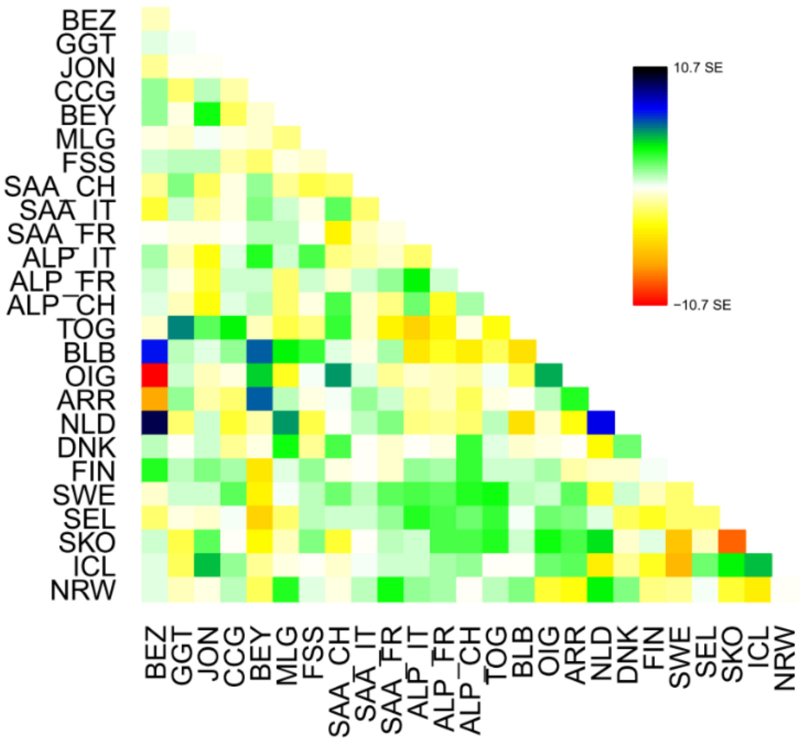
BEZ
GGT
JON
CCG
BEY
MLG
FSS
SAA_CH
SAA_IT
SAA_FR
ALP_IT
ALP_FR
ALP_CH
TOG
BLB
OIG
ARR
NLD
DNK
FIN
SWE
SEL
SKO
ICL
NRW
10.7 SE
−10.7 SE

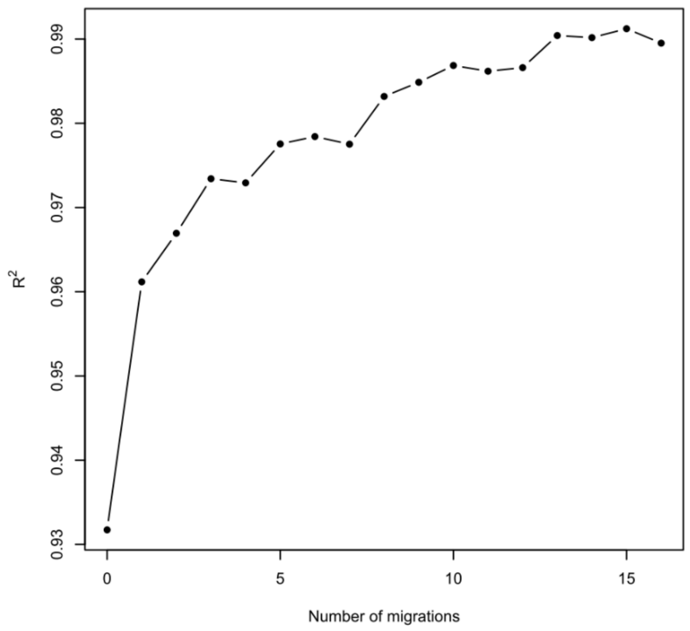

Supplementary Figure 5 A. Population graphs inferred by TREEMIX program using both datasets. The Maximum Likelihood trees inferred from the allele frequencies show the evolutionary relationships across breeds (nodes), where the branches' length s proportional to the evolutionary changes. Breeds are labelled with the same IDs code. The inferred admixture events range from 1 to 15 and are showed with arrows whose the colour is and indicator of the weight of the migration event that is the ancestry received from the source population (from yellow - 0 - to red - 0.5). Scaled residuals from the fit of the model to the data are displayed at the right side of each tree. The scale bar represents ten times the average standard error (SE) of the values in the sample covariance matrix.
