# Supplementary material for: Genetic diversity and historical demography of underutilised goat breeds in North-Western Europe
Source: Sci Rep. 2023 Nov 25;13:20728. doi: 10.1038/s41598-023-48005-8 (PMC10676416; doi:10.1038/s41598-023-48005-8)
Supplement: Supplementary file 5 — Supplementary Figure 5. [file 41598_2023_48005_MOESM5_ESM.pdf]

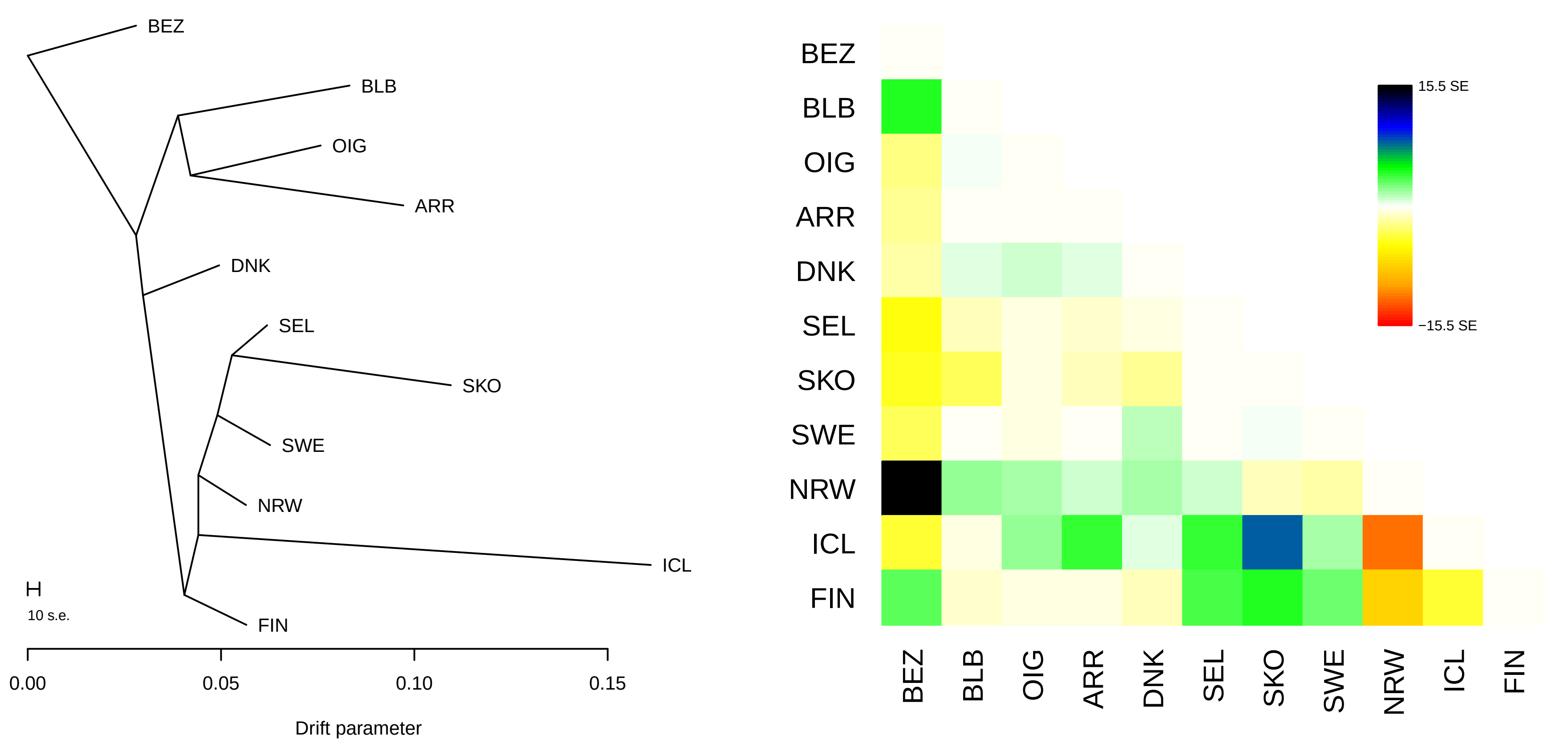
BEZ
BLB
OIG
ARR
DNK
SEL
SKO
SWE
NRW
ICL
FIN
10 s.e.
0.00 0.05 0.10 0.15
Drift parameter
15.5 SE
−15.5 SE

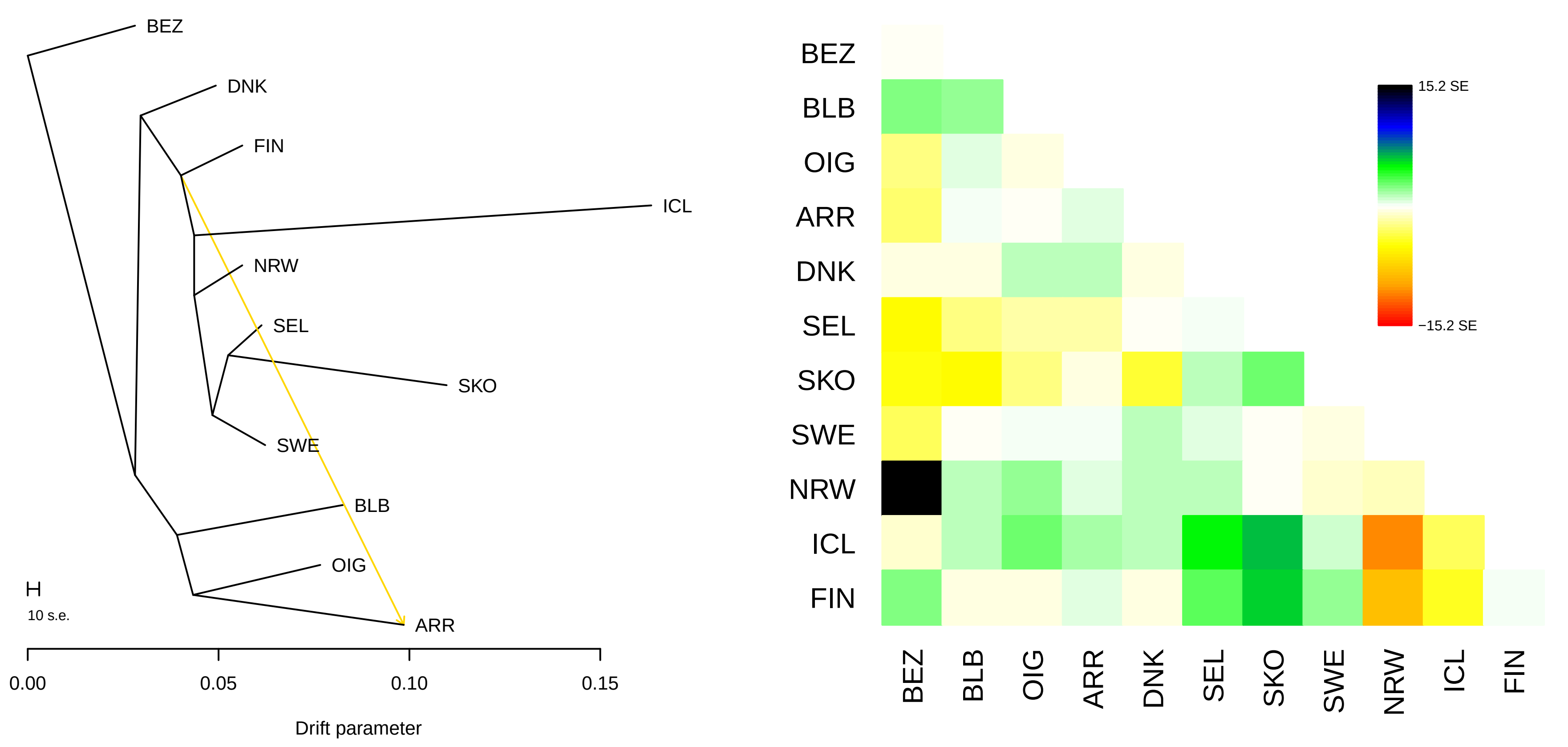
BEZ
DNK
FIN
ICL
NRW
SEL
SKO
SWE
BLB
OIG
ARR
10 s.e.
0.00 0.05 0.10 0.15
Drift parameter
15.2 SE
−15.2 SE

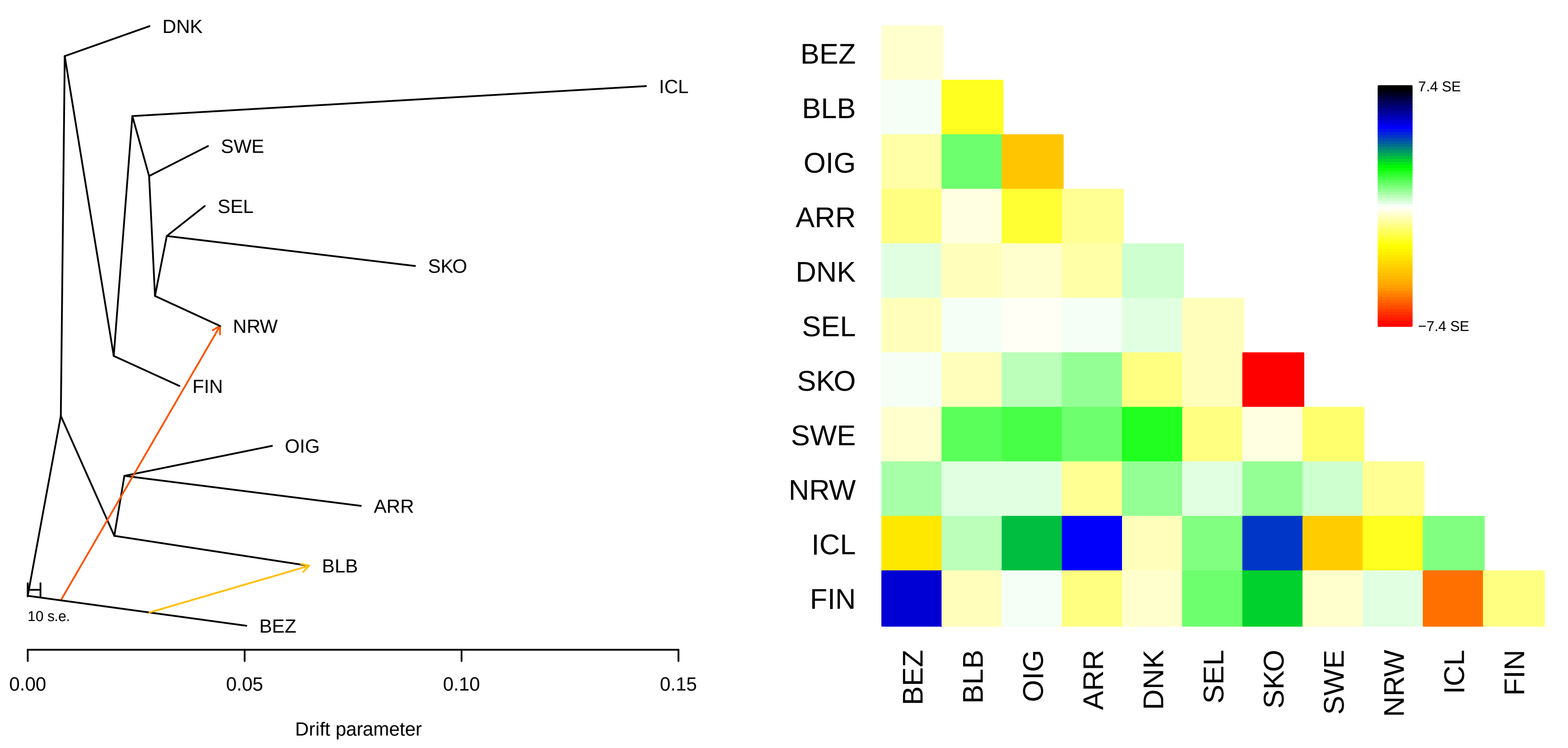
DNK
ICL
SWE
SEL
SKO
NRW
FIN
OIG
ARR
BLB
BEZ
10 s.e.
0.00 0.05 0.10 0.15
Drift parameter
7.4 SE
−7.4 SE

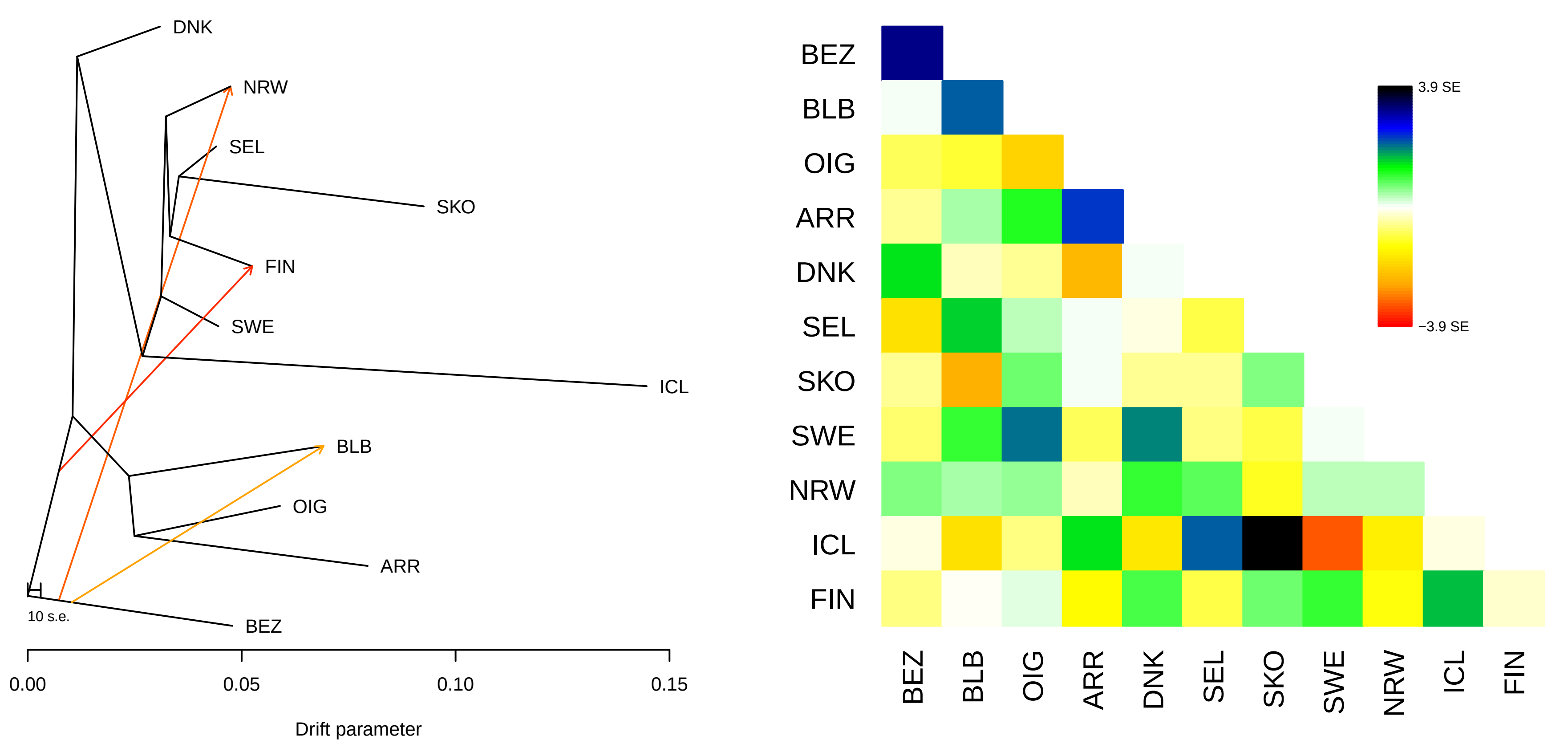
DNK
NRW
SEL
SKO
FIN
SWE
ICL
BLB
OIG
ARR
BEZ
10 s.e.
0.00 0.05 0.10 0.15
Drift parameter
3.9 SE
−3.9 SE

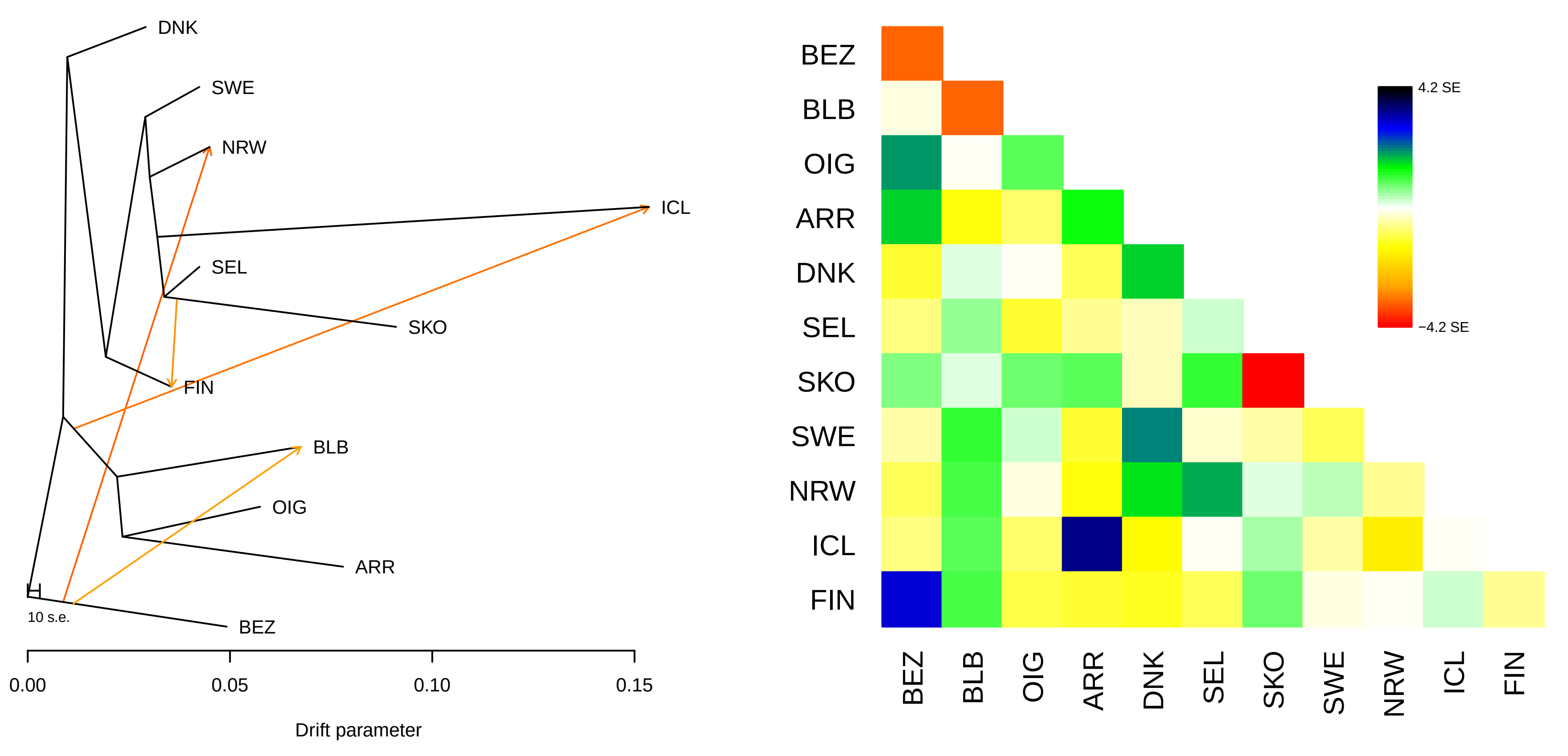
DNK
SWE
NRW
ICL
SEL
SKO
FIN
BLB
OIG
ARR
BEZ
10 s.e.
0.00 0.05 0.10 0.15
Drift parameter
4.2 SE
−4.2 SE

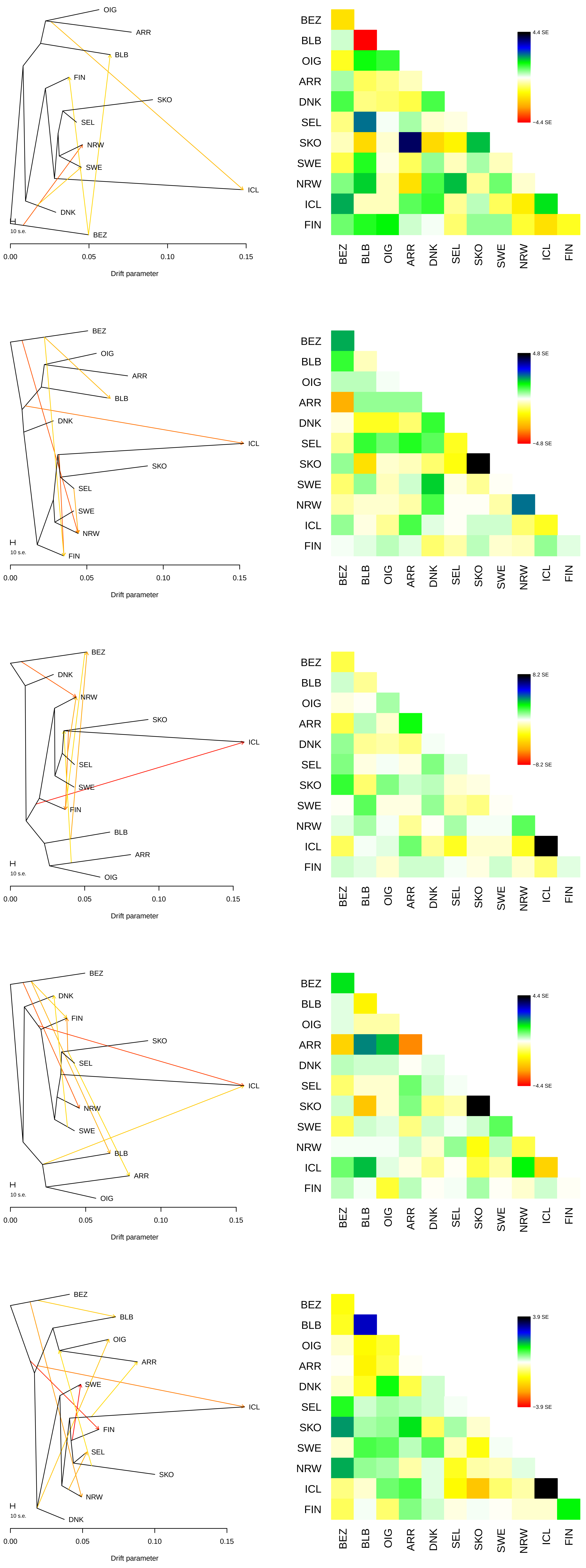

OIG
ARR
BLB
FIN
SKO
SEL
NRW
SWE
ICL
DNK
BEZ
10 s.e.
0.00
0.05
0.10
0.15
Drift parameter
BEZ BLB OIG ARR DNK SEL SKO SWE NRW ICL FIN
4.4 SE
−4.4 SE
BEZ
OIG
ARR
BLB
DNK
ICL
SKO
SEL
SWE
NRW
FIN
10 s.e.
0.00
0.05
0.10
0.15
Drift parameter
BEZ BLB OIG ARR DNK SEL SKO SWE NRW ICL FIN
4.8 SE
−4.8 SE
BEZ
DNK
NRW
SKO
ICL
SEL
SWE
FIN
BLB
ARR
OIG
10 s.e.
0.00
0.05
0.10
0.15
Drift parameter
BEZ BLB OIG ARR DNK SEL SKO SWE NRW ICL FIN
8.2 SE
−8.2 SE
BEZ
DNK
FIN
SKO
SEL
ICL
NRW
SWE
BLB
ARR
OIG
10 s.e.
0.00
0.05
0.10
0.15
Drift parameter
BEZ BLB OIG ARR DNK SEL SKO SWE NRW ICL FIN
4.4 SE
−4.4 SE
BEZ
BLB
OIG
ARR
SWE
ICL
FIN
SEL
SKO
NRW
DNK
10 s.e.
0.00
0.05
0.10
0.15
Drift parameter
BEZ BLB OIG ARR DNK SEL SKO SWE NRW ICL FIN
3.9 SE
−3.9 SE

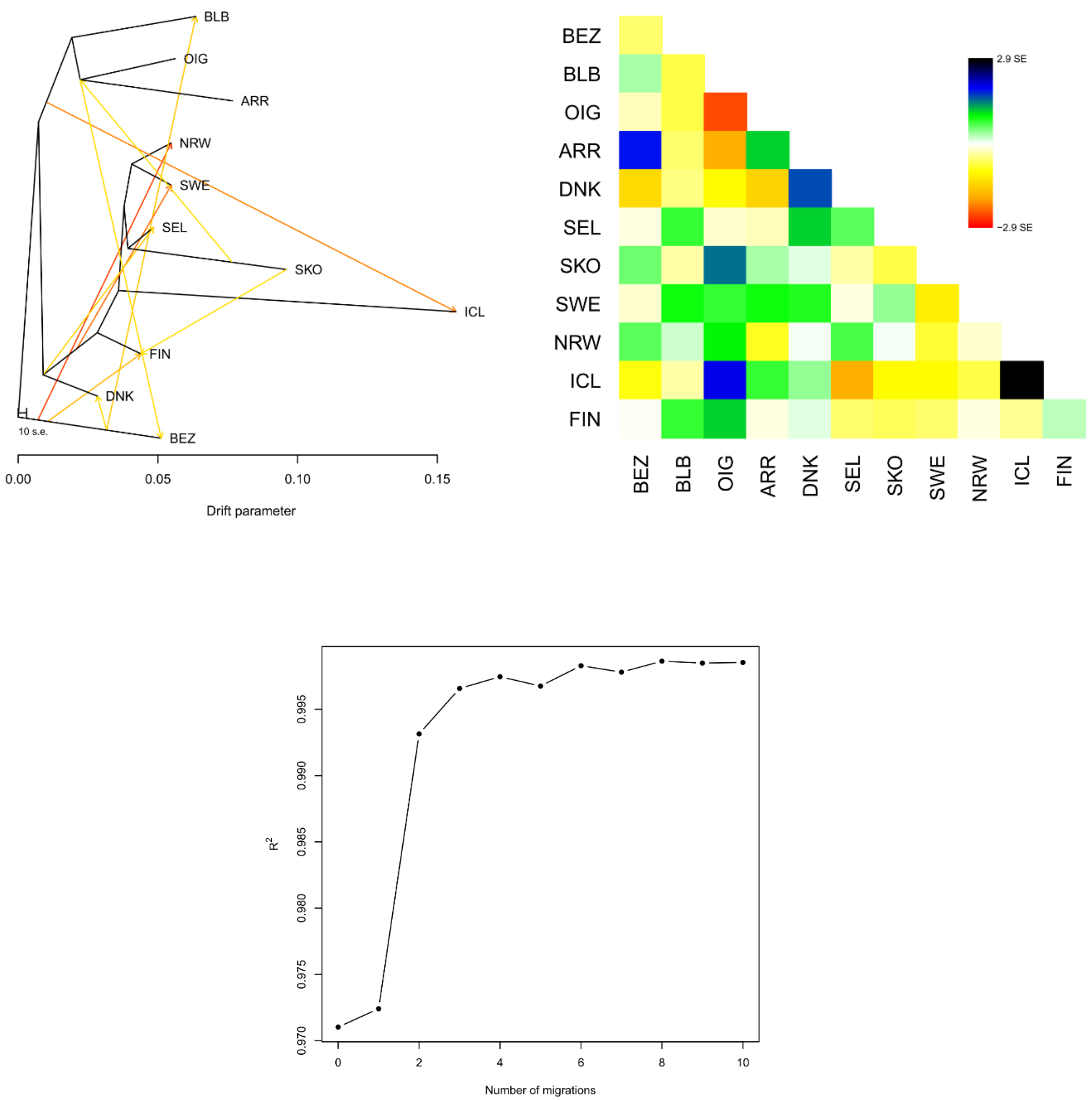

Supplementary Figure 5 B. Population graphs inferred by TREEMIX program using both datasets. The Maximum Likelihood trees inferred from the allele frequencies show the evolutionary

relationships across breeds (nodes), where the branches' length s proportional to the evolutionary changes. Breeds are labelled with the same IDs code. The inferred admixture events range from 1 to 10 and are showed with arrows whose the colour is and indicator of the weight of the migration event that is the ancestry received from the source population (from yellow - 0 - to red - 0.5). Scaled residuals from the fit of the model to the data are displayed at the right side of each tree. The scale bar represents ten times the average standard error (SE) of the values in the sample covariance matrix.
