# Supplementary material for: Genetic diversity and historical demography of underutilised goat breeds in North-Western Europe
Source: Sci Rep. 2023 Nov 25;13:20728. doi: 10.1038/s41598-023-48005-8 (PMC10676416; doi:10.1038/s41598-023-48005-8)
Supplement: Supplementary file 6 — Supplementary Figure 6. [file 41598_2023_48005_MOESM6_ESM.docx]

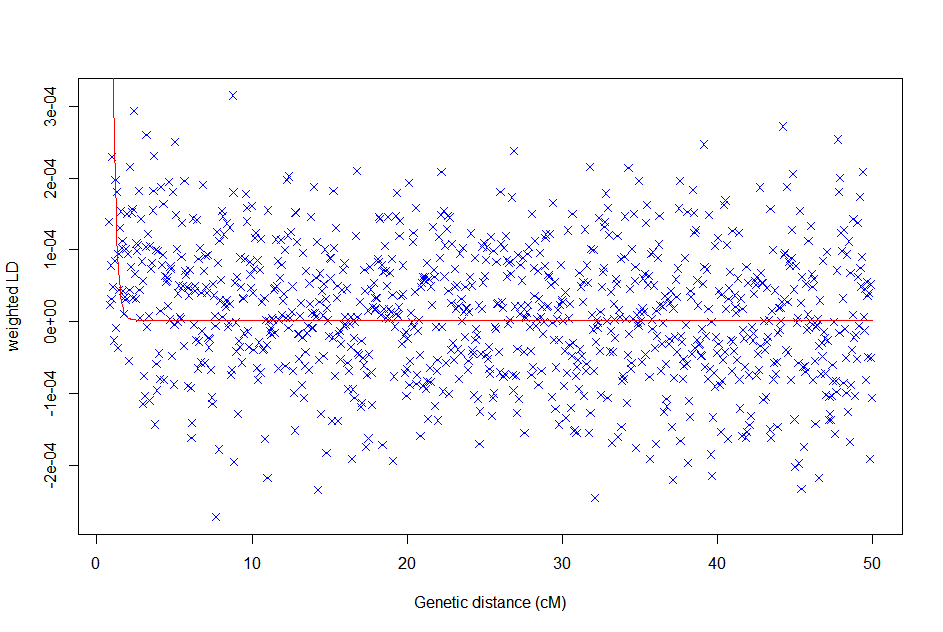

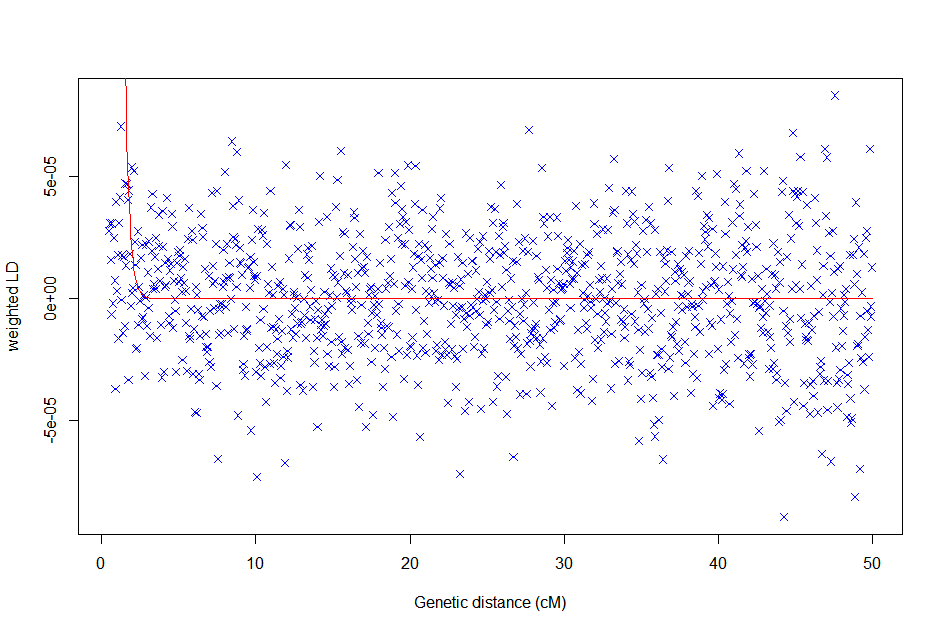


A) ICL|NRW|SEL B) BLB|NRW|OIG


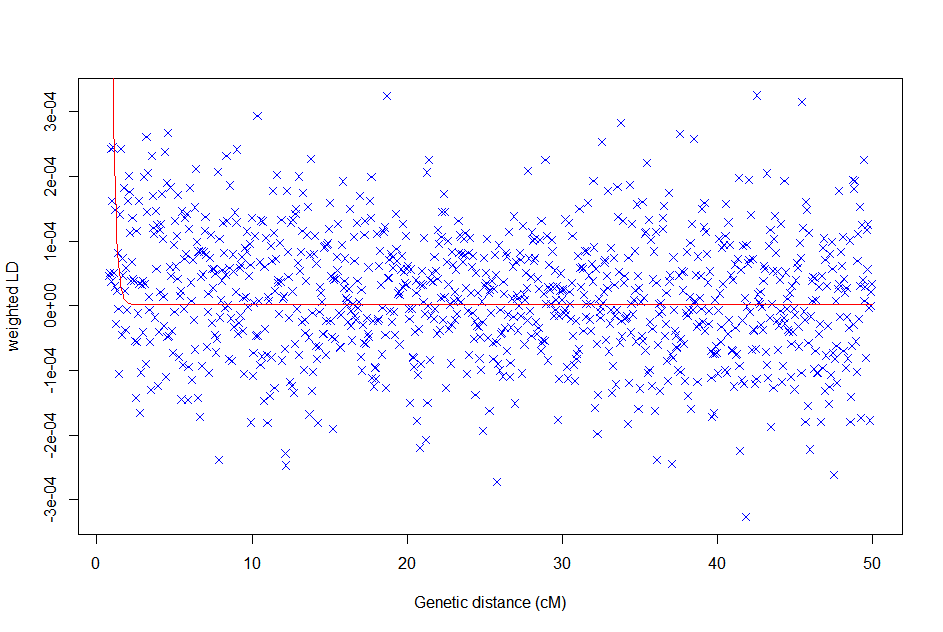

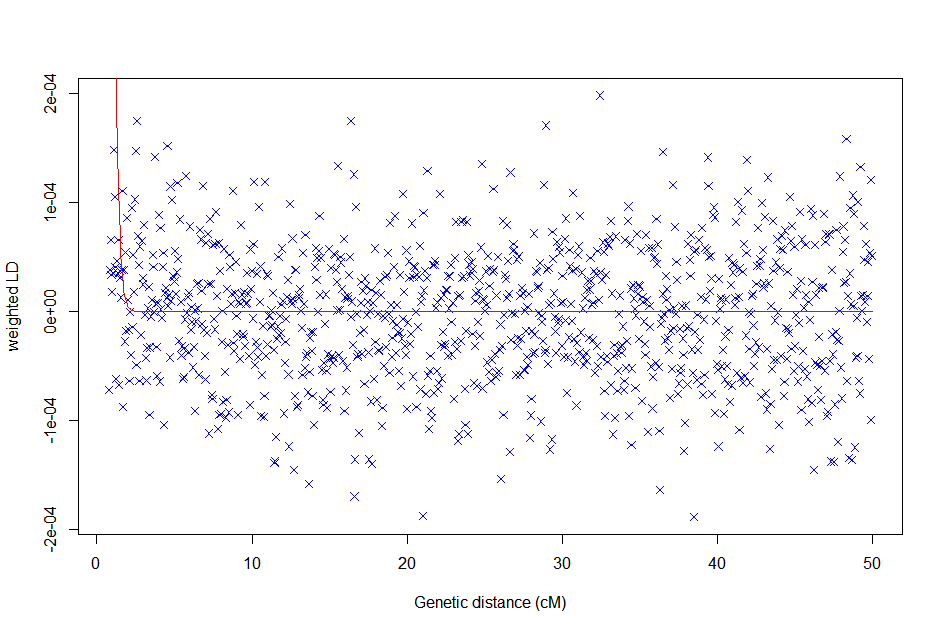


C) BLB|BEY|FSS D) OIG|SWE|BLB


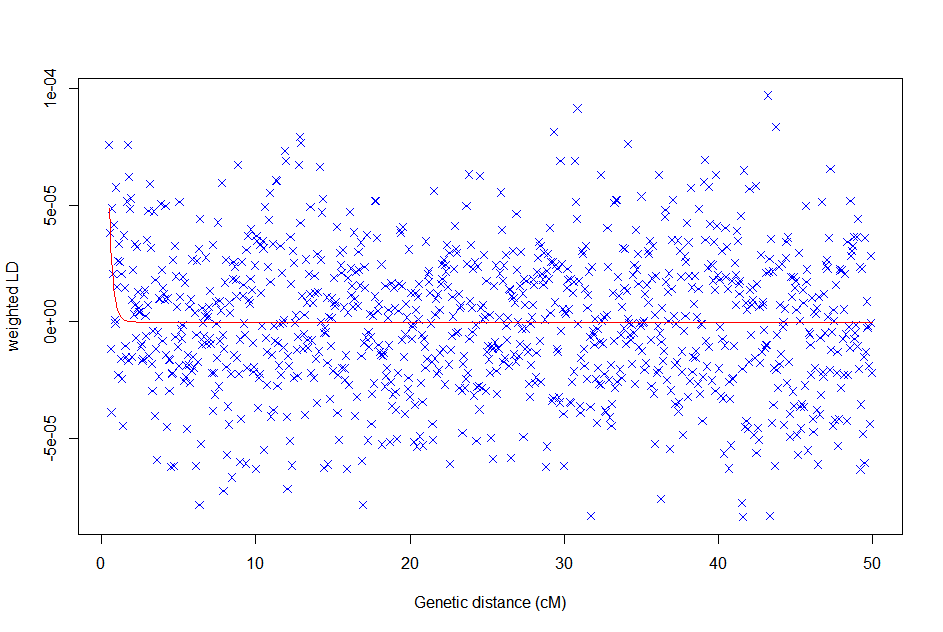


E) OIG|NRW|BLB

Supplementary Figure 6. Weighted LD decay curves with different pairs of source populations. Weighted LD curves for Icelandic landrace (ICL) using Norwegian landrace–Norwegian coastal Selje populations (A) and for the Irish breed Bilberry (BLB, B and C) and The Old Irish Goat (OIG, D-E), using both Scandinavian and Southern European breeds (Norwegian Landrace, NRW; Swedish Landrace, SWE; Fossée, FSS; Bermeya, BEY) as references. Data for each curve are plotted and fit starting from the corresponding ALDER-computed LD correlation thresholds. The best-fit exponential decay curve is labelled in red.
