# Supplementary material for: Genetic diversity and historical demography of underutilised goat breeds in North-Western Europe
Source: Sci Rep. 2023 Nov 25;13:20728. doi: 10.1038/s41598-023-48005-8 (PMC10676416; doi:10.1038/s41598-023-48005-8)
Supplement: Supplementary file 7 — Supplementary Table S1. [file 41598_2023_48005_MOESM7_ESM.docx]

Supplementary Table S1. Pairwise distance matrices. A) F_ST_ pairwise distance matrix. The analysis was performed with 100.000 permutations in Arlequin. B) Reynolds’ distance matrix (below) used in SplitTrees for the NeighbourNet calculation. Breeds’ codes are below as well as in Supplementary Table S8.

|  | ICL | SEL | SKO | NRW | SWE | FIN | DNK | ARR | BLB | OIG | NLD | FSS | BEY | MLG | GGT | CCG | JON | ALP_CH | ALP_FR | ALP_IT | SAA_CH | SAA_FR | SAA_IT | TOG |
| --- | --- | --- | --- | --- | --- | --- | --- | --- | --- | --- | --- | --- | --- | --- | --- | --- | --- | --- | --- | --- | --- | --- | --- | --- |
| ICL |  |  |  |  |  |  |  |  |  |  |  |  |  |  |  |  |  |  |  |  |  |  |  |  |
| SEL | 0.299 |  |  |  |  |  |  |  |  |  |  |  |  |  |  |  |  |  |  |  |  |  |  |  |
| SKO | 0.486 | 0.154 |  |  |  |  |  |  |  |  |  |  |  |  |  |  |  |  |  |  |  |  |  |  |
| NRW | 0.312 | 0.074 | 0.175 |  |  |  |  |  |  |  |  |  |  |  |  |  |  |  |  |  |  |  |  |  |
| SWE | 0.295 | 0.069 | 0.171 | 0.08 |  |  |  |  |  |  |  |  |  |  |  |  |  |  |  |  |  |  |  |  |
| FIN | 0.316 | 0.088 | 0.184 | 0.088 | 0.092 |  |  |  |  |  |  |  |  |  |  |  |  |  |  |  |  |  |  |  |
| DNK | 0.297 | 0.12 | 0.203 | 0.104 | 0.12 | 0.108 |  |  |  |  |  |  |  |  |  |  |  |  |  |  |  |  |  |  |
| ARR | 0.475 | 0.227 | 0.346 | 0.21 | 0.226 | 0.215 | 0.189 |  |  |  |  |  |  |  |  |  |  |  |  |  |  |  |  |  |
| BLB | 0.437 | 0.2 | 0.309 | 0.18 | 0.2 | 0.186 | 0.164 | 0.243 |  |  |  |  |  |  |  |  |  |  |  |  |  |  |  |  |
| OIG | 0.406 | 0.189 | 0.288 | 0.171 | 0.188 | 0.175 | 0.153 | 0.213 | 0.194 |  |  |  |  |  |  |  |  |  |  |  |  |  |  |  |
| NLD | 0.417 | 0.194 | 0.297 | 0.179 | 0.196 | 0.186 | 0.171 | 0.28 | 0.25 | 0.238 |  |  |  |  |  |  |  |  |  |  |  |  |  |  |
| FSS | 0.282 | 0.116 | 0.192 | 0.092 | 0.119 | 0.102 | 0.082 | 0.154 | 0.129 | 0.117 | 0.146 |  |  |  |  |  |  |  |  |  |  |  |  |  |
| BEY | 0.328 | 0.148 | 0.226 | 0.116 | 0.151 | 0.132 | 0.103 | 0.178 | 0.151 | 0.14 | 0.171 | 0.05 |  |  |  |  |  |  |  |  |  |  |  |  |
| MLG | 0.298 | 0.134 | 0.207 | 0.098 | 0.136 | 0.118 | 0.094 | 0.169 | 0.142 | 0.136 | 0.155 | 0.048 | 0.033 |  |  |  |  |  |  |  |  |  |  |  |
| GGT | 0.361 | 0.177 | 0.26 | 0.15 | 0.178 | 0.161 | 0.136 | 0.221 | 0.195 | 0.185 | 0.208 | 0.097 | 0.113 | 0.1 |  |  |  |  |  |  |  |  |  |  |
| CCG | 0.317 | 0.121 | 0.202 | 0.091 | 0.122 | 0.103 | 0.083 | 0.168 | 0.137 | 0.13 | 0.155 | 0.044 | 0.06 | 0.048 | 0.076 |  |  |  |  |  |  |  |  |  |
| JON | 0.387 | 0.162 | 0.258 | 0.126 | 0.162 | 0.143 | 0.12 | 0.22 | 0.186 | 0.176 | 0.198 | 0.08 | 0.095 | 0.073 | 0.111 | 0.056 |  |  |  |  |  |  |  |  |
| ALP_CH | 0.303 | 0.133 | 0.212 | 0.114 | 0.135 | 0.12 | 0.097 | 0.174 | 0.15 | 0.138 | 0.167 | 0.059 | 0.078 | 0.079 | 0.117 | 0.064 | 0.105 |  |  |  |  |  |  |  |
| ALP_FR | 0.29 | 0.124 | 0.199 | 0.104 | 0.128 | 0.111 | 0.091 | 0.161 | 0.139 | 0.127 | 0.155 | 0.047 | 0.065 | 0.066 | 0.108 | 0.054 | 0.094 | 0.053 |  |  |  |  |  |  |
| ALP_IT | 0.286 | 0.119 | 0.195 | 0.099 | 0.122 | 0.106 | 0.085 | 0.158 | 0.134 | 0.123 | 0.151 | 0.043 | 0.062 | 0.063 | 0.104 | 0.049 | 0.09 | 0.037 | 0.012 |  |  |  |  |  |
| SAA_CH | 0.329 | 0.155 | 0.237 | 0.135 | 0.156 | 0.141 | 0.115 | 0.194 | 0.169 | 0.156 | 0.184 | 0.078 | 0.096 | 0.096 | 0.135 | 0.082 | 0.125 | 0.086 | 0.082 | 0.076 |  |  |  |  |
| SAA_FR | 0.276 | 0.11 | 0.185 | 0.089 | 0.112 | 0.097 | 0.08 | 0.151 | 0.125 | 0.118 | 0.142 | 0.046 | 0.063 | 0.061 | 0.1 | 0.047 | 0.085 | 0.06 | 0.05 | 0.045 | 0.067 |  |  |  |
| SAA_IT | 0.301 | 0.115 | 0.195 | 0.092 | 0.117 | 0.1 | 0.081 | 0.158 | 0.131 | 0.122 | 0.149 | 0.045 | 0.063 | 0.061 | 0.102 | 0.046 | 0.087 | 0.059 | 0.051 | 0.045 | 0.062 | 0.008 |  |  |
| TOG | 0.37 | 0.17 | 0.263 | 0.151 | 0.17 | 0.157 | 0.13 | 0.219 | 0.192 | 0.18 | 0.207 | 0.093 | 0.115 | 0.11 | 0.151 | 0.096 | 0.142 | 0.104 | 0.098 | 0.092 | 0.122 | 0.091 | 0.092 |  |

**A**

|  | ICL | SEL | SKO | NRW | SWE | FIN | DNK | ARR | BLB | OIG | NLD | FSS | BEY | MLG | GGT | CCG | JON | ALP_CH | ALP_FR | ALP_IT | SAA_CH | SAA_FR | SAA_IT | TOG |
| --- | --- | --- | --- | --- | --- | --- | --- | --- | --- | --- | --- | --- | --- | --- | --- | --- | --- | --- | --- | --- | --- | --- | --- | --- |
| ICL |  |  |  |  |  |  |  |  |  |  |  |  |  |  |  |  |  |  |  |  |  |  |  |  |
| SEL | 0.355 |  |  |  |  |  |  |  |  |  |  |  |  |  |  |  |  |  |  |  |  |  |  |  |
| SKO | 0.665 | 0.168 |  |  |  |  |  |  |  |  |  |  |  |  |  |  |  |  |  |  |  |  |  |  |
| NRW | 0.374 | 0.077 | 0.193 |  |  |  |  |  |  |  |  |  |  |  |  |  |  |  |  |  |  |  |  |  |
| SWE | 0.349 | 0.072 | 0.188 | 0.084 |  |  |  |  |  |  |  |  |  |  |  |  |  |  |  |  |  |  |  |  |
| FIN | 0.380 | 0.092 | 0.204 | 0.093 | 0.096 |  |  |  |  |  |  |  |  |  |  |  |  |  |  |  |  |  |  |  |
| DNK | 0.352 | 0.128 | 0.227 | 0.110 | 0.128 | 0.114 |  |  |  |  |  |  |  |  |  |  |  |  |  |  |  |  |  |  |
| ARR | 0.644 | 0.257 | 0.424 | 0.236 | 0.256 | 0.242 | 0.210 |  |  |  |  |  |  |  |  |  |  |  |  |  |  |  |  |  |
| BLB | 0.575 | 0.224 | 0.369 | 0.198 | 0.223 | 0.206 | 0.179 | 0.278 |  |  |  |  |  |  |  |  |  |  |  |  |  |  |  |  |
| OIG | 0.521 | 0.209 | 0.339 | 0.187 | 0.209 | 0.192 | 0.166 | 0.239 | 0.216 |  |  |  |  |  |  |  |  |  |  |  |  |  |  |  |
| NLD | 0.540 | 0.216 | 0.353 | 0.197 | 0.218 | 0.205 | 0.187 | 0.329 | 0.288 | 0.272 |  |  |  |  |  |  |  |  |  |  |  |  |  |  |
| FSS | 0.332 | 0.123 | 0.213 | 0.096 | 0.126 | 0.108 | 0.085 | 0.168 | 0.138 | 0.124 | 0.158 |  |  |  |  |  |  |  |  |  |  |  |  |  |
| BEY | 0.397 | 0.161 | 0.256 | 0.124 | 0.163 | 0.142 | 0.109 | 0.196 | 0.164 | 0.150 | 0.188 | 0.051 |  |  |  |  |  |  |  |  |  |  |  |  |
| MLG | 0.354 | 0.144 | 0.232 | 0.103 | 0.146 | 0.125 | 0.099 | 0.186 | 0.154 | 0.146 | 0.169 | 0.049 | 0.034 |  |  |  |  |  |  |  |  |  |  |  |
| GGT | 0.447 | 0.195 | 0.302 | 0.162 | 0.195 | 0.175 | 0.147 | 0.250 | 0.216 | 0.205 | 0.233 | 0.101 | 0.120 | 0.105 |  |  |  |  |  |  |  |  |  |  |
| CCG | 0.382 | 0.128 | 0.226 | 0.095 | 0.130 | 0.109 | 0.087 | 0.183 | 0.148 | 0.139 | 0.168 | 0.045 | 0.061 | 0.050 | 0.079 |  |  |  |  |  |  |  |  |  |
| JON | 0.489 | 0.177 | 0.299 | 0.135 | 0.177 | 0.154 | 0.127 | 0.249 | 0.205 | 0.193 | 0.221 | 0.084 | 0.100 | 0.076 | 0.118 | 0.057 |  |  |  |  |  |  |  |  |
| ALP_CH | 0.361 | 0.143 | 0.238 | 0.121 | 0.145 | 0.128 | 0.102 | 0.192 | 0.163 | 0.149 | 0.182 | 0.061 | 0.082 | 0.082 | 0.125 | 0.066 | 0.111 |  |  |  |  |  |  |  |
| ALP_FR | 0.342 | 0.133 | 0.221 | 0.110 | 0.136 | 0.118 | 0.095 | 0.176 | 0.149 | 0.136 | 0.168 | 0.048 | 0.067 | 0.068 | 0.114 | 0.055 | 0.099 | 0.054 |  |  |  |  |  |  |
| ALP_IT | 0.338 | 0.126 | 0.217 | 0.104 | 0.130 | 0.112 | 0.089 | 0.172 | 0.144 | 0.132 | 0.164 | 0.044 | 0.064 | 0.065 | 0.109 | 0.050 | 0.094 | 0.038 | 0.012 |  |  |  |  |  |
| SAA_CH | 0.399 | 0.168 | 0.270 | 0.145 | 0.169 | 0.153 | 0.122 | 0.216 | 0.185 | 0.170 | 0.204 | 0.081 | 0.101 | 0.101 | 0.145 | 0.086 | 0.133 | 0.090 | 0.086 | 0.079 |  |  |  |  |
| SAA_FR | 0.323 | 0.116 | 0.204 | 0.093 | 0.119 | 0.102 | 0.083 | 0.164 | 0.133 | 0.126 | 0.153 | 0.047 | 0.066 | 0.063 | 0.106 | 0.048 | 0.089 | 0.061 | 0.051 | 0.046 | 0.069 |  |  |  |
| SAA_IT | 0.358 | 0.122 | 0.217 | 0.097 | 0.125 | 0.105 | 0.085 | 0.172 | 0.140 | 0.130 | 0.162 | 0.046 | 0.065 | 0.063 | 0.108 | 0.048 | 0.091 | 0.061 | 0.052 | 0.047 | 0.064 | 0.008 |  |  |
| TOG | 0.462 | 0.186 | 0.305 | 0.164 | 0.187 | 0.171 | 0.140 | 0.247 | 0.213 | 0.199 | 0.232 | 0.098 | 0.122 | 0.117 | 0.164 | 0.101 | 0.154 | 0.109 | 0.103 | 0.097 | 0.130 | 0.095 | 0.097 |  |

**B**

Icelandic Landrace, ICL; Norwegian coastal (Skorpa), SKO; Nowegian coastal (Selje), SEL; Swedish Landrace, SWE; Danish Landrace, DNK; Dutch Landrace, NLD; Finnish Landrace, FIN; Traditional Aran, ARR; Bilberry, BLB; Old Irish Goat, OIG; Fosseé, FSS; Alpine (France), ALP_FR; Saanen (France), SAA_FR; Alpine (Switzerland), ALP_CH; Saanen (Switzerland), SAA_CH; Toggenburg, TOG; Bermeya, BEY; Malaguena, MLG; Girgentana, GGT; Ciociara Grigia, CCG; Jonica, JON; Alpine, ALP_IT; Saanen, SAA_IT; Norwegian Landrace, NRW.
