# Supplementary material for: Genetic diversity and historical demography of underutilised goat breeds in North-Western Europe
Source: Sci Rep. 2023 Nov 25;13:20728. doi: 10.1038/s41598-023-48005-8 (PMC10676416; doi:10.1038/s41598-023-48005-8)
Supplement: Supplementary file 8 — Supplementary Table S2. [file 41598_2023_48005_MOESM8_ESM.docx]

Supplementary Table S2. Observed heterozygosity (OBS-H) and expected heterozygosity (EXP-H) estimate for each breed of the WHOLE dataset. The mean of the genomic inbreeding coefficient (FROH) estimates is calculated only for our target breeds of NW dataset. Breed code is in Supplementary Table S8.

| Population | Obs-H | Exp H | FROH |
| --- | --- | --- | --- |
| ICL | 0.30255 | 0.35324 | 0.655 |
| SEL | 0.37297 | 0.37718 | 0.077 |
| SKO | 0.39500 | 0.39999 | 0.325 |
| NRW | 0.40381 | 0.39821 | 0.079 |
| SWE | 0.36993 | 0.37159 | 0.093 |
| FIN | 0.38391 | 0.38893 | 0.065 |
| DNK | 0.36619 | 0.39388 | 0.168 |
| ARR | 0.35145 | 0.36235 | 0.306 |
| BLB | 0.39086 | 0.38105 | 0.211 |
| OIG | 0.38517 | 0.37655 | 0.150 |
| NLD | 0.36845 | 0.36234 | - |
| FSS | 0.41238 | 0.42113 | - |
| BEY | 0.41205 | 0.41079 | - |
| MLG | 0.42320 | 0.42364 | - |
| GGT | 0.37476 | 0.38348 | - |
| CCG | 0.40840 | 0.42624 | - |
| JON | 0.43746 | 0.40683 | - |
| ALP_CH | 0.40291 | 0.40152 | - |
| ALP_FR | 0.42284 | 0.41881 | - |
| ALP_IT | 0.41270 | 0.41777 | - |
| SAA_CH | 0.39016 | 0.38939 | - |
| SAA_FR | 0.42832 | 0.42626 | - |
| SAA_IT | 0.42364 | 0.42332 | - |
| TOG | 0.37524 | 0.37559 | - |
