# Supplementary material for: Genetic diversity and historical demography of underutilised goat breeds in North-Western Europe
Source: Sci Rep. 2023 Nov 25;13:20728. doi: 10.1038/s41598-023-48005-8 (PMC10676416; doi:10.1038/s41598-023-48005-8)
Supplement: Supplementary file 9 — Supplementary Table S3. [file 41598_2023_48005_MOESM9_ESM.docx]

Supplementary Table S3. Whole genome FROH estimates for each individual (IID) in each breed (FID).

| IID | FID | Sum | FROH_genome |
| --- | --- | --- | --- |
| DKCH-LNR-000002073 | DNK | 94620915 | 0.038418 |
| DKCH-LNR-000002075 | DNK | 8.51E+08 | 0.345629 |
| DKCH-LNR-000002076 | DNK | 2.49E+08 | 0.100969 |
| DKCH-LNR-000002080 | DNK | 3.67E+08 | 0.148965 |
| DKCH-LNR-000002083 | DNK | 1.91E+08 | 0.077562 |
| DKCH-LNR-000002084 | DNK | 4.09E+08 | 0.165923 |
| DKCH-LNR-000002090 | DNK | 2.41E+08 | 0.097728 |
| DKCH-LNR-000002094 | DNK | 1.53E+08 | 0.062167 |
| DKCH-LNR-000002095 | DNK | 5.55E+08 | 0.225199 |
| DKCH-LNR-000002097 | DNK | 1.32E+08 | 0.053791 |
| DKCH-LNR-000002101 | DNK | 5.79E+08 | 0.234897 |
| DKCH-LNR-000002102 | DNK | 4.02E+08 | 0.163254 |
| DKCH-LNR-000002105 | DNK | 2.74E+08 | 0.111102 |
| DKCH-LNR-000002106 | DNK | 1.18E+08 | 0.047895 |
| DKCH-LNR-000002110 | DNK | 2.8E+08 | 0.113724 |
| DKCH-LNR-000002116 | DNK | 4.28E+08 | 0.173897 |
| DKCH-LNR-000002119 | DNK | 1.48E+08 | 0.060292 |
| DKCH-LNR-000002121 | DNK | 4.36E+08 | 0.176985 |
| DKCH-LNR-000002123 | DNK | 6.82E+08 | 0.277036 |
| DKCH-LNR-000002125 | DNK | 3.48E+08 | 0.141446 |
| DKCH-LNR-000002127 | DNK | 4.25E+08 | 0.172669 |
| DKCH-LNR-000002130 | DNK | 2.47E+08 | 0.100192 |
| DKCH-LNR-000002131 | DNK | 6E+08 | 0.24356 |
| DKCH-LNR-000002134 | DNK | 1.4E+08 | 0.056753 |
| DKCH-LNR-000002139 | DNK | 2.3E+08 | 0.093262 |
| DKCH-LNR-000002140 | DNK | 8.53E+08 | 0.34645 |
| DKCH-LNR-000002141 | DNK | 3.36E+08 | 0.136389 |
| DKCH-LNR-000002143 | DNK | 3.92E+08 | 0.159004 |
| DKCH-LNR-000002148 | DNK | 5.78E+08 | 0.234624 |
| DKCH-LNR-000002149 | DNK | 4.36E+08 | 0.177157 |
| DKCH-LNR-000002150 | DNK | 4.55E+08 | 0.184543 |
| DKCH-LNR-000002152 | DNK | 4.58E+08 | 0.186139 |
| DKCH-LNR-000002154 | DNK | 5.57E+08 | 0.225974 |
| DKCH-LNR-000002158 | DNK | 1.29E+08 | 0.052276 |
| DKCH-LNR-000002161 | DNK | 1.87E+08 | 0.07608 |
| DKCH-LNR-000002162 | DNK | 6.49E+08 | 0.263341 |
| DKCH-LNR-000002163 | DNK | 3.87E+08 | 0.156991 |
| DKCH-LNR-000002166 | DNK | 3.16E+08 | 0.1284 |
| DKCH-LNR-000002167 | DNK | 3.55E+08 | 0.144 |
| DKCH-LNR-000002169 | DNK | 3.28E+08 | 0.133275 |
| DKCH-LNR-000002170 | DNK | 4.41E+08 | 0.17918 |
| DKCH-LNR-000002171 | DNK | 8.16E+08 | 0.331146 |
| DKCH-LNR-000002172 | DNK | 5.76E+08 | 0.233768 |
| DKCH-LNR-000002175 | DNK | 5.01E+08 | 0.203288 |
| DKCH-LNR-000002176 | DNK | 6.48E+08 | 0.262914 |
| DKCH-LNR-000002179 | DNK | 9.88E+08 | 0.401173 |
| DKCH-LNR-000002187 | DNK | 1.79E+08 | 0.072747 |
| DKCH-LNR-000002188 | DNK | 3.28E+08 | 0.133345 |
| DKCH-LNR-000002190 | DNK | 1.06E+09 | 0.428383 |
| DKCH-LNR-000002192 | DNK | 2.25E+08 | 0.091345 |
| FICH-LNR-000004022 | FIN | 1.06E+08 | 0.043075 |
| FICH-LNR-000004023 | FIN | 62516323 | 0.025383 |
| FICH-LNR-000004024 | FIN | 83179692 | 0.033773 |
| FICH-LNR-000004025 | FIN | 99399586 | 0.040358 |
| FICH-LNR-000004026 | FIN | 1.79E+08 | 0.072628 |
| FICH-LNR-000004027 | FIN | 1.37E+08 | 0.055756 |
| FICH-LNR-000004028 | FIN | 1.98E+08 | 0.080398 |
| FICH-LNR-000004029 | FIN | 1.41E+08 | 0.057197 |
| FICH-LNR-000004030 | FIN | 1.45E+08 | 0.058848 |
| FICH-LNR-000004031 | FIN | 1.55E+08 | 0.062808 |
| FICH-LNR-000004032 | FIN | 1.55E+08 | 0.063129 |
| FICH-LNR-000004033 | FIN | 1.5E+08 | 0.060785 |
| FICH-LNR-000004034 | FIN | 1.22E+08 | 0.04947 |
| FICH-LNR-000004035 | FIN | 92887537 | 0.037714 |
| FICH-LNR-000004036 | FIN | 91184260 | 0.037023 |
| FICH-LNR-000004037 | FIN | 4.32E+08 | 0.175397 |
| FICH-LNR-000004039 | FIN | 4.45E+08 | 0.180854 |
| FICH-LNR-000004040 | FIN | 1.22E+08 | 0.04948 |
| FICH-LNR-000004041 | FIN | 1.4E+08 | 0.056988 |
| IECH-ARR-000004094 | ARR | 6.96E+08 | 0.282573 |
| IECH-ARR-000004095 | ARR | 6.78E+08 | 0.275423 |
| IECH-ARR-000004097 | ARR | 5.64E+08 | 0.22911 |
| IECH-ARR-000004099 | ARR | 1.2E+09 | 0.488354 |
| IECH-ARR-000004100 | ARR | 8.22E+08 | 0.333816 |
| IECH-ARR-000004101 | ARR | 9.02E+08 | 0.366428 |
| IECH-ARR-000004102 | ARR | 4.86E+08 | 0.19739 |
| IECH-ARR-000004103 | ARR | 7.72E+08 | 0.313417 |
| IECH-ARR-000004104 | ARR | 6.7E+08 | 0.272227 |
| IECH-BLB-000004105 | BLB | 3.45E+08 | 0.140054 |
| IECH-BLB-000004106 | BLB | 7.51E+08 | 0.304746 |
| IECH-BLB-000004107 | BLB | 79280601 | 0.032189 |
| IECH-BLB-000004108 | BLB | 5.55E+08 | 0.22526 |
| IECH-BLB-000004109 | BLB | 5.87E+08 | 0.238341 |
| IECH-BLB-000004110 | BLB | 5.33E+08 | 0.216351 |
| IECH-BLB-000004111 | BLB | 5.46E+08 | 0.221495 |
| IECH-BLB-000004112 | BLB | 4.39E+08 | 0.178345 |
| IECH-BLB-000004113 | BLB | 7.69E+08 | 0.312242 |
| IECH-BLB-000004114 | BLB | 6.03E+08 | 0.244678 |
| IECH-OIG-000000291 | OIG | 3.46E+08 | 0.140425 |
| IECH-OIG-000000292 | OIG | 2.14E+08 | 0.086738 |
| IECH-OIG-000004072 | OIG | 4.54E+08 | 0.184514 |
| IECH-OIG-000004073 | OIG | 3.53E+08 | 0.143146 |
| IECH-OIG-000004075 | OIG | 5.56E+08 | 0.225616 |
| IECH-OIG-000004079 | OIG | 3.03E+08 | 0.122917 |
| IECH-OIG-000004080 | OIG | 2.98E+08 | 0.120991 |
| IECH-OIG-000004081 | OIG | 5.35E+08 | 0.217073 |
| IECH-OIG-000004083 | OIG | 3.94E+08 | 0.15994 |
| IECH-OIG-000004084 | OIG | 39961716 | 0.016225 |
| IECH-OIG-000004086 | OIG | 3.78E+08 | 0.153506 |
| IECH-OIG-000004087 | OIG | 3.71E+08 | 0.150818 |
| IECH-OIG-000004092 | OIG | 3.58E+08 | 0.145219 |
| IECH-OIG-000004093 | OIG | 5.77E+08 | 0.234453 |
| ISCH-ICL-000004042 | ICL | 1.67E+09 | 0.679513 |
| ISCH-ICL-000004043 | ICL | 1.53E+09 | 0.621713 |
| ISCH-ICL-000004044 | ICL | 1.96E+09 | 0.79481 |
| ISCH-ICL-000004045 | ICL | 1.78E+09 | 0.721401 |
| ISCH-ICL-000004046 | ICL | 1.56E+09 | 0.633193 |
| ISCH-ICL-000004048 | ICL | 1.35E+09 | 0.547069 |
| ISCH-ICL-000004050 | ICL | 1.71E+09 | 0.692488 |
| ISCH-ICL-000004051 | ICL | 1.57E+09 | 0.637032 |
| ISCH-ICL-000004052 | ICL | 1.52E+09 | 0.615634 |
| ISCH-ICL-000004053 | ICL | 1.49E+09 | 0.604028 |
| SECH-LNR-000004655 | SWE | 4.24E+08 | 0.171953 |
| SECH-LNR-000004656 | SWE | 1.77E+08 | 0.071821 |
| SECH-LNR-000004657 | SWE | 1.41E+08 | 0.057358 |
| SECH-LNR-000004659 | SWE | 1.17E+08 | 0.047617 |
| SECH-LNR-000004660 | SWE | 1.71E+08 | 0.069525 |
| SECH-LNR-000004661 | SWE | 2.64E+08 | 0.107124 |
| SECH-LNR-000004662 | SWE | 1.91E+08 | 0.07764 |
| SECH-LNR-000004663 | SWE | 2.24E+08 | 0.090892 |
| SECH-LNR-000004664 | SWE | 1.94E+08 | 0.078912 |
| SECH-LNR-000004665 | SWE | 1.8E+08 | 0.072923 |
| SECH-LNR-000004666 | SWE | 2.16E+08 | 0.087687 |
| SECH-LNR-000004667 | SWE | 1.08E+08 | 0.043654 |
| SECH-LNR-000004668 | SWE | 2.58E+08 | 0.10467 |
| SECH-LNR-000004669 | SWE | 1.87E+08 | 0.075974 |
| SECH-LNR-000004670 | SWE | 1.88E+08 | 0.076165 |
| SECH-LNR-000004671 | SWE | 1.37E+08 | 0.055738 |
| SECH-LNR-000004672 | SWE | 2.18E+08 | 0.088566 |
| SECH-LNR-000004674 | SWE | 2.22E+08 | 0.08999 |
| SECH-LNR-000004675 | SWE | 2.22E+08 | 0.090178 |
| SECH-LNR-000004677 | SWE | 1.8E+08 | 0.073269 |
| SECH-LNR-000004678 | SWE | 2.59E+08 | 0.104999 |
| SECH-LNR-000004679 | SWE | 2.64E+08 | 0.107041 |
| SECH-LNR-000004680 | SWE | 2.71E+08 | 0.110088 |
| SECH-LNR-000004681 | SWE | 1.97E+08 | 0.080138 |
| SECH-LNR-000004682 | SWE | 2.94E+08 | 0.119349 |
| SECH-LNR-000004683 | SWE | 2.54E+08 | 0.103186 |
| SECH-LNR-000004685 | SWE | 2.84E+08 | 0.115423 |
| SECH-LNR-000004686 | SWE | 2.26E+08 | 0.091796 |
| SECH-LNR-000004687 | SWE | 4.46E+08 | 0.181075 |
| SECH-LNR-000004688 | SWE | 2.64E+08 | 0.10699 |
| SECH-LNR-000004689 | SWE | 4.27E+08 | 0.17332 |
| SECH-LNR-000004690 | SWE | 3.53E+08 | 0.143167 |
| SECH-LNR-000004691 | SWE | 2.46E+08 | 0.099785 |
| SECH-LNR-000004692 | SWE | 2.25E+08 | 0.091374 |
| SECH-LNR-000004693 | SWE | 1.86E+08 | 0.075646 |
| SECH-LNR-000004694 | SWE | 2.49E+08 | 0.101028 |
| SECH-LNR-000004695 | SWE | 3.14E+08 | 0.127307 |
| SECH-LNR-000004696 | SWE | 3.66E+08 | 0.148423 |
| SECH-LNR-000004697 | SWE | 1.34E+08 | 0.054545 |
| SECH-LNR-000004698 | SWE | 80028656 | 0.032493 |
| SECH-LNR-000004699 | SWE | 1.89E+08 | 0.07684 |
| SECH-LNR-000004700 | SWE | 2.97E+08 | 0.120609 |
| SECH-LNR-000004701 | SWE | 31710373 | 0.012875 |
| SEL_1035654 | SEL | 56975220 | 0.023133 |
| SEL_1035656 | SEL | 2.87E+08 | 0.116618 |
| SEL_961700 | SEL | 1.64E+08 | 0.06675 |
| SEL_961702 | SEL | 3.51E+08 | 0.142524 |
| SEL_961704 | SEL | 2.97E+08 | 0.120444 |
| SEL_961708 | SEL | 3.93E+08 | 0.159408 |
| SEL_961710 | SEL | 2.33E+08 | 0.0947 |
| SEL_961714 | SEL | 27033539 | 0.010976 |
| SEL_961716 | SEL | 5.49E+08 | 0.222871 |
| SEL_961718 | SEL | 63967182 | 0.025972 |
| SEL_961720 | SEL | 33664578 | 0.013668 |
| SEL_961724 | SEL | 33516341 | 0.013608 |
| SEL_961726 | SEL | 1.01E+08 | 0.040995 |
| SEL_961728 | SEL | 76227012 | 0.03095 |
| SEL_961730 | SEL | 2.49E+08 | 0.100934 |
| SEL_961736 | SEL | 4.9E+08 | 0.199116 |
| SEL_961738 | SEL | 2.08E+08 | 0.084599 |
| SEL_961740 | SEL | 67587882 | 0.027442 |
| SEL_961742 | SEL | 2.44E+08 | 0.098899 |
| SEL_961744 | SEL | 45814685 | 0.018602 |
| SEL_961746 | SEL | 4.01E+08 | 0.16278 |
| SEL_961748 | SEL | 4.3E+08 | 0.174642 |
| SEL_961750 | SEL | 24519574 | 0.009955 |
| SEL_961754 | SEL | 23034136 | 0.009352 |
| SEL_961756 | SEL | 55864351 | 0.022682 |
| SEL_961758 | SEL | 82989101 | 0.033695 |
| SEL_961762 | SEL | 1.03E+08 | 0.041815 |
| SEL_961764 | SEL | 2.44E+08 | 0.098937 |
| SKO_1035657 | SKO | 6.82E+08 | 0.276744 |
| SKO_1035658 | SKO | 7.68E+08 | 0.311769 |
| SKO_1062645 | SKO | 8.91E+08 | 0.361904 |
| SKO_1062646 | SKO | 9.23E+08 | 0.374956 |
| SKO_1062648 | SKO | 7.14E+08 | 0.28996 |
| SKO_1062649 | SKO | 8.3E+08 | 0.337186 |
| TZCH-NRW-000003553 | NRW | 2.66E+08 | 0.107872 |
| TZCH-NRW-000003554 | NRW | 3.04E+08 | 0.123418 |
| TZCH-NRW-000003555 | NRW | 4.84E+08 | 0.196314 |
| TZCH-NRW-000003556 | NRW | 1.85E+08 | 0.075253 |
| TZCH-NRW-000003557 | NRW | 1.4E+08 | 0.05695 |
| TZCH-NRW-000003575 | NRW | 37650429 | 0.015287 |
| TZCH-NRW-000003576 | NRW | 1.64E+08 | 0.066479 |
| TZCH-NRW-000003577 | NRW | 1.15E+08 | 0.04683 |
| TZCH-NRW-000003579 | NRW | 2.44E+08 | 0.098888 |
| TZCH-NRW-000003580 | NRW | 3.76E+08 | 0.152738 |
| TZCH-NRW-000003581 | NRW | 34843059 | 0.014147 |
| TZCH-NRW-000003582 | NRW | 80808408 | 0.03281 |
| TZCH-NRW-000003583 | NRW | 88440934 | 0.035909 |
| TZCH-NRW-000003584 | NRW | 3.86E+08 | 0.156668 |
| TZCH-NRW-000003586 | NRW | 93726086 | 0.038055 |
| TZCH-NRW-000003587 | NRW | 1.31E+08 | 0.053278 |
