# Supplementary material for: Genetic diversity and historical demography of underutilised goat breeds in North-Western Europe
Source: Sci Rep. 2023 Nov 25;13:20728. doi: 10.1038/s41598-023-48005-8 (PMC10676416; doi:10.1038/s41598-023-48005-8)
Supplement: Supplementary file 10 — Supplementary Table S4. [file 41598_2023_48005_MOESM10_ESM.docx]

Supplementary Table S4 A. Output of BITE for the TreeMix results relative to the WHOLE dataset, with the total of variance explained “Var.expl” estimated for each migration event for the total number of migrations tested - 15 in our study - (i.e. computed value of f for each TreeMix model), “mign” is the number of significant migrations (p-val < 0.05) that implies a significant improvement of the fit to the data. The last two colums indicate the tree and graph log-likelihoods.

| m | Var.expl | msign | perc | llik_0 | llik_m |
| --- | --- | --- | --- | --- | --- |
| 0 | 0.93172 | 0 | 0 | -4015.31 | -4015.31 |
| 1 | 0.96117 | 1 | 100 | -3692.23 | -1361.74 |
| 2 | 0.96694 | 2 | 100 | -3663.85 | -928.584 |
| 3 | 0.97342 | 3 | 100 | -3622.33 | -393.753 |
| 4 | 0.97293 | 4 | 100 | -3305.89 | 50.9881 |
| 5 | 0.97755 | 5 | 100 | -3385.76 | 393.707 |
| 6 | 0.97842 | 6 | 100 | -3412.79 | 486.909 |
| 7 | 0.97752 | 7 | 100 | -3478.57 | 530.84 |
| 8 | 0.98319 | 8 | 100 | -3355.6 | 914.888 |
| 9 | 0.98487 | 9 | 100 | -3338.6 | 1187.31 |
| 10 | 0.98686 | 10 | 100 | -3897.78 | 1205.7 |
| 11 | 0.98618 | 11 | 100 | -3559.43 | 1054.65 |
| 12 | 0.98661 | 11 | 91.6 | -3575.49 | 1352.27 |
| 13 | 0.99042 | 13 | 100 | -3690.37 | 1466.98 |
| 14 | 0.99017 | 13 | 92.8 | -3396.25 | 1513.55 |
| 15 | 0.99123 | 15 | 100 | -3732.34 | 1541.8 |

Supplementary Table S4 B. Output of BITE for the TreeMix analysis relative to the WHOLE dataset and 15 migration events. The first two columns report the first population encountered in the donor subgraph and the first population encountered in the receiving subgraph. The edge weight is the estimated fraction of ancestry in the receiving subgraph derived from the donor fraction also estimated via jackknife, standard error and the p-value associated to the weight. The breed code is indicated in Table.

| stree.don | stree.rec | edge.weight | edge.weight.jk | jk.st.err | p-value |
| --- | --- | --- | --- | --- | --- |
| SAA_CH | SAA_FR | 0.441171 | 0.527362 | 0.0375278 | 2.22507e-308 |
| BEZ | GGT | 0.546544 | 0.552982 | 0.0435255 | 2.22507e-308 |
| BEZ | GGT | 0.164191 | 0.167324 | 0.00786365 | 2.22507e-308 |
| BEZ | MLG | 0.265313 | 0.26921 | 0.0101131 | 2.22507e-308 |
| BEZ | NRW | 0.202958 | 0.208767 | 0.00674079 | 2.22507e-308 |
| BEZ | JON | 0.30041 | 0.282285 | 0.0165197 | 2.22507e-308 |
| NLD | BEZ | 0.390564 | 0.392514 | 0.0188543 | 2.22507e-308 |
| BEY | OIG | 0.0732873 | 0.0804397 | 0.0082356 | 2.22507e-308 |
| SKO | FIN | 0.257298 | 0.276493 | 0.0386592 | 4.27436e-13 |
| GGT | DNK | 0.451386 | 0.459046 | 0.0238394 | 2.22507e-308 |
| BEY | NLD | 0.362368 | 0.385385 | 0.0343838 | 2.22507e-308 |
| BEZ | FIN | 0.050312 | 0.037772 | 0.00727632 | 1.04536e-07 |
| DNK | OIG | 0.156306 | 0.157555 | 0.0140675 | 2.22507e-308 |
| OIG | FSS | 0.382085 | 0.36522 | 0.0353521 | 2.22507e-308 |
| MLG | GGT | 0.124797 | 0.0906903 | 0.0142608 | 1.01254e-10 |
