# Supplementary material for: Genetic diversity and historical demography of underutilised goat breeds in North-Western Europe
Source: Sci Rep. 2023 Nov 25;13:20728. doi: 10.1038/s41598-023-48005-8 (PMC10676416; doi:10.1038/s41598-023-48005-8)
Supplement: Supplementary file 11 — Supplementary Table S5. [file 41598_2023_48005_MOESM11_ESM.docx]

Supplementary Table S5 A. Output of BITE for the TreeMix analysis of the target NW-EU dataset, with the total of variance “Var.expl” estimated for each migration event for the total number of migrations tested - 8 in this case - (i.e. computed value of f for each TreeMix model), “mign” is the number of significant migrations (p-val < 0.05) that implies a significant improvement of the fit to the data. The last two colums indicate the tree and graph log-likelihoods.

| m | Var.expl | msign | perc | llik_0 | llik_m |
| --- | --- | --- | --- | --- | --- |
| 0 | 0.97103 | 0 | 0 | -81.7191 | -81.7191 |
| 1 | 0.97241 | 0 | 0 | -6365.46 | -114.407 |
| 2 | 0.99314 | 2 | 100 | -101 | 383.182 |
| 3 | 0.99657 | 3 | 100 | -119.947 | 430.403 |
| 4 | 0.99746 | 4 | 100 | -96.3607 | 442.147 |
| 5 | 0.99675 | 3 | 60 | -74.5299 | 448.677 |
| 6 | 0.99829 | 3 | 50 | -88.1479 | 460.941 |
| 7 | 0.9978 | 6 | 85.7 | -43.7994 | 460.489 |
| 8 | 0.99863 | 6 | 75 | -78.3205 | 463.277 |
| 9 | 0.99849 | 7 | 77.7 | -93.8242 | 467.172 |
| 10 | 0.99853 | 5 | 50 | -59.3898 | 464.569 |

Supplementary Table S5 B. Output of BITE for the TreeMix analysis for 8 migration events. The first two columns report the first population encountered in the donor subgraph and the first population encountered in the receiving subgraph. The edge weight is the estimated fraction of ancestry in the receiving subgraph derived from the donor fraction also estimated via jackknife, standard error and the p-value associated to the weight. The breed code is indicated in Table.

| stree.don | stree.rec | edge.weight | edge.weight.jk | jk.st.err | pvalue |
| --- | --- | --- | --- | --- | --- |
| BEZ | NRW | 0.301544 | 0.293875 | 0.0139987 | 2.22507e-308 |
| SKO | FIN | 0.192726 | 0.193163 | 0.041686 | 1.7953e-06 |
| BEZ | FIN | 0.0667478 | 0.0683625 | 0.0147467 | 1.77794e-06 |
| BEZ | BLB | 0.122983 | 0.0936663 | 0.0495019 | 0.0292339 |
| DNK | ICL | 0.367396 | 0.353384 | 0.0584457 | 7.40785e-10 |
| SKO | ARR | 0.032894 | 0.00339122 | 0.00656062 | 0.302611 |
| BLB | ICL | 0.0622316 | 0.0589113 | 0.0289919 | 0.0210773 |
| SWE | DNK | 0.027575 | 0.0426986 | 0.0303386 | 0.0796539 |
