# Supplementary material for: Genetic diversity and historical demography of underutilised goat breeds in North-Western Europe
Source: Sci Rep. 2023 Nov 25;13:20728. doi: 10.1038/s41598-023-48005-8 (PMC10676416; doi:10.1038/s41598-023-48005-8)
Supplement: Supplementary file 12 — Supplementary Table S6. [file 41598_2023_48005_MOESM12_ESM.docx]

Supplementary Table S6A. F4 statistic results for all breeds combinations. The f4 test investigates the tree topology of four populations ((A,B),(C,D)) and generates an f4 score. A deviation from zero of this score indicates more gene flow (admixture) between a certain pair of the quartet than between the other pairs We retained only the estimates with +20 < Z-score < -20.

| A | B | C | D | f4-estimate | SE | Z-score | p-value |
| --- | --- | --- | --- | --- | --- | --- | --- |
|  |  |  |  |  |  |  |  |
| BEZ | NRW | SWE | FSS | -0.01797 | 4.73E-04 | -37.9978 | 0 |
| BEZ | SWE | NRW | FSS | -0.01461 | 4.71E-04 | -31.0344 | 1.86E-211 |
| BEZ | FSS | NRW | SWE | 0.003356 | 3.43E-04 | 9.792617 | 1.21E-22 |
| BEZ | NRW | SWE | CCG | -0.0215 | 4.78E-04 | -45.0204 | 0 |
| BEZ | SWE | NRW | CCG | -0.01827 | 4.87E-04 | -37.5011 | 8.82E-308 |
| BEZ | CCG | NRW | SWE | 0.003226 | 3.32E-04 | 9.727193 | 2.31E-22 |
| BEZ | BEY | MLG | CCG | -0.00885 | 3.39E-04 | -26.0866 | 5.17E-150 |
| BEZ | MLG | BEY | CCG | -0.01278 | 3.51E-04 | -36.4124 | 2.71E-290 |
| BEZ | CCG | BEY | MLG | -0.00393 | 2.44E-04 | -16.101 | 2.51E-58 |
| SEL | NRW | ARR | JON | 0.004326 | 4.01E-04 | 10.78311 | 4.14E-27 |
| SEL | ARR | NRW | JON | 0.02 | 5.70E-04 | 35.08197 | 1.27E-269 |
| SEL | JON | NRW | ARR | 0.015674 | 6.03E-04 | 25.98486 | 7.34E-149 |
| SEL | NRW | BLB | BEY | 0.003282 | 3.32E-04 | 9.879124 | 5.13E-23 |
| SEL | BLB | NRW | BEY | 0.024584 | 5.25E-04 | 46.81336 | 0 |
| SEL | BEY | NRW | BLB | 0.021303 | 5.44E-04 | 39.13776 | 0 |
| SEL | NRW | BLB | MLG | 0.004746 | 3.32E-04 | 14.29434 | 2.37E-46 |
| SEL | BLB | NRW | MLG | 0.022614 | 4.75E-04 | 47.59511 | 0 |
| SEL | MLG | NRW | BLB | 0.017868 | 5.12E-04 | 34.92693 | 2.90E-267 |
| SEL | NRW | BLB | JON | 0.00369 | 3.73E-04 | 9.901244 | 4.11E-23 |
| SEL | BLB | NRW | JON | 0.020428 | 5.37E-04 | 38.04203 | 0 |
| SEL | JON | NRW | BLB | 0.016738 | 5.52E-04 | 30.3099 | 8.49E-202 |
| SEL | NRW | OIG | BEY | 0.003656 | 3.18E-04 | 11.51125 | 1.16E-30 |
| SEL | OIG | NRW | BEY | 0.025361 | 5.10E-04 | 49.69995 | 0 |
| SEL | BEY | NRW | OIG | 0.021704 | 5.35E-04 | 40.60141 | 0 |
| SEL | NRW | OIG | MLG | 0.00512 | 3.17E-04 | 16.16669 | 8.66E-59 |
| SEL | OIG | NRW | MLG | 0.022325 | 4.69E-04 | 47.57482 | 0 |
| SEL | MLG | NRW | OIG | 0.017205 | 5.04E-04 | 34.16866 | 7.06E-256 |
| SEL | NRW | OIG | JON | 0.004074 | 3.58E-04 | 11.36774 | 6.05E-30 |
| SEL | OIG | NRW | JON | 0.020472 | 5.11E-04 | 40.08042 | 0 |
| SEL | JON | NRW | OIG | 0.016398 | 5.32E-04 | 30.81355 | 1.73E-208 |
| SEL | NRW | NLD | BEY | 0.004041 | 3.53E-04 | 11.44451 | 2.51E-30 |
| SEL | NLD | NRW | BEY | 0.018831 | 4.97E-04 | 37.91973 | 0 |
| SEL | BEY | NRW | NLD | 0.01479 | 5.58E-04 | 26.52021 | 5.67E-155 |
| SEL | NRW | FSS | BEY | 0.001805 | 1.93E-04 | 9.33961 | 9.67E-21 |
| SEL | FSS | NRW | BEY | 0.028796 | 4.15E-04 | 69.33895 | 0 |
| SEL | BEY | NRW | FSS | 0.026991 | 4.24E-04 | 63.60217 | 0 |
| SEL | NRW | FSS | MLG | 0.003269 | 1.91E-04 | 17.09665 | 1.57E-65 |
| SEL | FSS | NRW | MLG | 0.026755 | 3.90E-04 | 68.6563 | 0 |
| SEL | MLG | NRW | FSS | 0.023486 | 4.04E-04 | 58.13045 | 0 |
| SEL | NRW | MLG | CCG | -0.00265 | 2.13E-04 | -12.4242 | 1.93E-35 |
| SEL | MLG | NRW | CCG | 0.022973 | 4.23E-04 | 54.32211 | 0 |
| SEL | CCG | NRW | MLG | 0.025622 | 4.01E-04 | 63.85966 | 0 |
| NRW | SWE | DNK | MLG | -0.0054 | 2.72E-04 | -19.8633 | 8.46E-88 |
| NRW | DNK | SWE | MLG | 0.01107 | 4.11E-04 | 26.96438 | 3.87E-160 |
| NRW | MLG | SWE | DNK | 0.016473 | 3.73E-04 | 44.2106 | 0 |
| NRW | SWE | DNK | GGT | -0.00308 | 3.20E-04 | -9.6389 | 5.48E-22 |
| NRW | DNK | SWE | GGT | 0.012279 | 4.49E-04 | 27.36138 | ######## |
| NRW | GGT | SWE | DNK | 0.015364 | 4.18E-04 | 36.76348 | ######## |
| NRW | SWE | DNK | CCG | -0.00264 | 2.70E-04 | -9.78994 | 1.24E-22 |
| NRW | DNK | SWE | CCG | 0.011786 | 3.83E-04 | 30.73503 | 1.94E-207 |
| NRW | CCG | SWE | DNK | 0.014426 | 3.66E-04 | 39.36246 | 0 |
| NRW | SWE | ARR | BEY | -0.00382 | 3.81E-04 | -10.0323 | 1.10E-23 |
| NRW | ARR | SWE | BEY | 0.019665 | 5.75E-04 | 34.19137 | 3.25E-256 |
| NRW | BEY | SWE | ARR | 0.023485 | 5.40E-04 | 43.47631 | 0 |
| NRW | SWE | ARR | MLG | -0.0052 | 3.78E-04 | -13.7685 | 3.94E-43 |
| NRW | ARR | SWE | MLG | 0.015531 | 5.44E-04 | 28.57037 | 1.57E-179 |
| NRW | MLG | SWE | ARR | 0.02073 | 4.90E-04 | 42.34468 | 0 |
| NRW | SWE | BLB | BEY | -0.00337 | 3.41E-04 | -9.87625 | 5.28E-23 |
| NRW | BLB | SWE | BEY | 0.019907 | 5.49E-04 | 36.26477 | 5.81E-288 |
| NRW | BEY | SWE | BLB | 0.023279 | 5.22E-04 | 44.59675 | 0 |
| NRW | SWE | BLB | MLG | -0.00475 | 3.42E-04 | -13.9006 | 6.28E-44 |
| NRW | BLB | SWE | MLG | 0.016475 | 5.10E-04 | 32.29278 | 8.83E-229 |
| NRW | MLG | SWE | BLB | 0.021226 | 4.73E-04 | 44.88831 | 0 |
| NRW | SWE | OIG | BEY | -0.0036 | 3.28E-04 | -10.9741 | 5.09E-28 |
| NRW | OIG | SWE | BEY | 0.020458 | 5.32E-04 | 38.43304 | 0 |
| NRW | BEY | SWE | OIG | 0.024061 | 4.99E-04 | 48.22522 | 0 |
| NRW | SWE | OIG | MLG | -0.00498 | 3.23E-04 | -15.428 | 1.06E-53 |
| NRW | OIG | SWE | MLG | 0.015963 | 4.97E-04 | 32.11145 | 3.05E-226 |
| NRW | MLG | SWE | OIG | 0.020945 | 4.59E-04 | 45.60223 | 0 |
| NRW | SWE | OIG | JON | -0.00373 | 3.77E-04 | -9.90489 | 3.96E-23 |
| NRW | OIG | SWE | JON | 0.015157 | 5.36E-04 | 28.29359 | 4.14E-176 |
| NRW | JON | SWE | OIG | 0.018891 | 4.97E-04 | 38.04223 | 0 |
| NRW | FIN | FSS | MLG | -0.00253 | 2.12E-04 | -11.9211 | 9.19E-33 |
| NRW | FSS | FIN | MLG | 0.017052 | 3.73E-04 | 45.74109 | 0 |
| NRW | MLG | FIN | FSS | 0.019584 | 3.55E-04 | 55.14638 | 0 |
| SWE | DNK | BEY | CCG | -0.0028 | 2.81E-04 | -9.97628 | 1.94E-23 |
| SWE | BEY | DNK | CCG | 0.009745 | 3.55E-04 | 27.45743 | 5.66E-166 |
| SWE | CCG | DNK | BEY | 0.012547 | 3.39E-04 | 36.99424 | 1.42E-299 |
| SWE | OIG | BEY | MLG | -0.00312 | 2.60E-04 | -11.9712 | 5.03E-33 |
| SWE | BEY | OIG | MLG | 0.013521 | 4.79E-04 | 28.20135 | 5.63E-175 |
| SWE | MLG | OIG | BEY | 0.016638 | 4.44E-04 | 37.45584 | 4.83E-307 |
| FIN | OIG | BEY | MLG | -0.00327 | 2.57E-04 | -12.7498 | 3.12E-37 |
| FIN | BEY | OIG | MLG | 0.013122 | 4.62E-04 | 28.39915 | 2.07E-177 |
| FIN | MLG | OIG | BEY | 0.016396 | 4.26E-04 | 38.49924 | 0 |
| SEL | NRW | ARR | JON | 0.004326 | 4.01E-04 | 10.78311 | 4.14E-27 |
| SEL | ARR | NRW | JON | 0.02 | 5.70E-04 | 35.08197 | 1.27E-269 |
| SEL | JON | NRW | ARR | 0.015674 | 6.03E-04 | 25.98486 | 7.34E-149 |
| SEL | NRW | BLB | BEY | 0.003282 | 3.32E-04 | 9.879124 | 5.13E-23 |
| SEL | BLB | NRW | BEY | 0.024584 | 5.25E-04 | 46.81336 | 0 |
| SEL | BEY | NRW | BLB | 0.021303 | 5.44E-04 | 39.13776 | 0 |
| SEL | NRW | BLB | MLG | 0.004746 | 3.32E-04 | 14.29434 | 2.37E-46 |
| SEL | BLB | NRW | MLG | 0.022614 | 4.75E-04 | 47.59511 | 0 |
| SEL | MLG | NRW | BLB | 0.017868 | 5.12E-04 | 34.92693 | 2.90E-267 |
| SEL | NRW | BLB | JON | 0.00369 | 3.73E-04 | 9.901244 | 4.11E-23 |
| SEL | BLB | NRW | JON | 0.020428 | 5.37E-04 | 38.04203 | 0 |
| SEL | JON | NRW | BLB | 0.016738 | 5.52E-04 | 30.3099 | 8.49E-202 |
| SEL | NRW | OIG | MLG | 0.00512 | 3.17E-04 | 16.16669 | 8.66E-59 |
| SEL | OIG | NRW | MLG | 0.022325 | 4.69E-04 | 47.57482 | 0 |
| SEL | MLG | NRW | OIG | 0.017205 | 5.04E-04 | 34.16866 | 7.06E-256 |
| SEL | NRW | OIG | JON | 0.004074 | 3.58E-04 | 11.36774 | 6.05E-30 |
| SEL | OIG | NRW | JON | 0.020472 | 5.11E-04 | 40.08042 | 0 |
| SEL | JON | NRW | OIG | 0.016398 | 5.32E-04 | 30.81355 | 1.73E-208 |
| SWE | DNK | OIG | BEY | 0.00384 | 3.74E-04 | 10.27227 | 9.40E-25 |
| SWE | OIG | DNK | BEY | 0.011095 | 4.12E-04 | 26.93173 | 9.34E-160 |
| SWE | BEY | DNK | OIG | 0.007255 | 4.63E-04 | 15.65937 | 2.87E-55 |
| SWE | DNK | BEY | CCG | -0.0028 | 2.81E-04 | -9.97628 | 1.94E-23 |
| SWE | BEY | DNK | CCG | 0.009745 | 3.55E-04 | 27.45743 | 5.66E-166 |
| SWE | CCG | DNK | BEY | 0.012547 | 3.39E-04 | 36.99424 | 1.42E-299 |
| SWE | OIG | BEY | MLG | -0.00312 | 2.60E-04 | -11.9712 | 5.03E-33 |
| SWE | BEY | OIG | MLG | 0.013521 | 4.79E-04 | 28.20135 | 5.63E-175 |
| SWE | MLG | OIG | BEY | 0.016638 | 4.44E-04 | 37.45584 | 4.83E-307 |
