# Supplementary material for: Genetic diversity and historical demography of underutilised goat breeds in North-Western Europe
Source: Sci Rep. 2023 Nov 25;13:20728. doi: 10.1038/s41598-023-48005-8 (PMC10676416; doi:10.1038/s41598-023-48005-8)
Supplement: Supplementary file 13 — Supplementary Table S6. [file 41598_2023_48005_MOESM13_ESM.docx]

Supplementary Table S6B. F4 statistic results for the target breeds. The f4 test investigates the tree topology of four populations ((A,B),(C,D)) and generates an f4 score. A deviation from zero of this score indicates more gene flow (admixture) between a certain pair of the quartet than between the other pairs We retained only the estimates with p-value < 0.0001.

| A | B | C | D | f4-estimate | | SE | Z-score | | p-value |
| --- | --- | --- | --- | --- | --- | --- | --- | --- | --- |
| BEZ | ICL | SEL | SWE | -0.00198 | 5.08E-04 | | -3.89021 | 1.00E-04 | |
| BEZ | SEL | ICL | SWE | 0.003125 | 7.38E-04 | | 4.232272 | 2.31E-05 | |
| BEZ | SWE | ICL | SEL | 0.0051 | 7.29E-04 | | 6.997096 | 2.61E-12 | |
| BEZ | ICL | SKO | FIN | -0.00828 | 8.87E-04 | | -9.33154 | 1.04E-20 | |
| BEZ | SKO | ICL | FIN | -0.00522 | 9.67E-04 | | -5.40143 | 6.61E-08 | |
| BEZ | FIN | ICL | SKO | 0.003058 | 8.08E-04 | | 3.785636 | 1.53E-04 | |
| BEZ | ICL | SWE | FIN | -0.0064 | 6.02E-04 | | -10.6399 | 1.94E-26 | |
| BEZ | SWE | ICL | FIN | -0.00441 | 7.67E-04 | | -5.75118 | 8.86E-09 | |
| BEZ | FIN | ICL | SWE | 0.001994 | 7.13E-04 | | 2.794828 | 0.005193 | |
| BEZ | ICL | FIN | ARR | -0.00831 | 9.44E-04 | | -8.80117 | 1.35E-18 | |
| BEZ | FIN | ICL | ARR | -0.01125 | 8.74E-04 | | -12.8633 | 7.24E-38 | |
| BEZ | ARR | ICL | FIN | -0.00293 | 9.05E-04 | | -3.24303 | 0.001183 | |
| BEZ | ICL | FIN | OIG | -0.00853 | 8.45E-04 | | -10.0925 | 5.96E-24 | |
| BEZ | FIN | ICL | OIG | -0.01104 | 8.37E-04 | | -13.1836 | 1.09E-39 | |
| BEZ | OIG | ICL | FIN | -0.00251 | 7.90E-04 | | -3.1728 | 0.00151 | |
| BEZ | ICL | ARR | BLB | -0.00371 | 9.64E-04 | | -3.84999 | 1.18E-04 | |
| BEZ | ARR | ICL | BLB | 0.006912 | 0.001088 | | 6.35366 | 2.10E-10 | |
| BEZ | BLB | ICL | ARR | 0.010623 | 0.001013 | | 10.4863 | 9.99E-26 | |
| BEZ | ICL | BLB | OIG | 0.00349 | 8.90E-04 | | 3.923208 | 8.74E-05 | |
| BEZ | BLB | ICL | OIG | 0.010635 | 9.57E-04 | | 11.11273 | 1.09E-28 | |
| BEZ | OIG | ICL | BLB | 0.007145 | 9.50E-04 | | 7.519595 | 5.49E-14 | |
| BEZ | SEL | FIN | ARR | -0.01204 | 6.84E-04 | | -17.6097 | 2.08E-69 | |
| BEZ | FIN | SEL | ARR | -0.01403 | 6.51E-04 | | -21.5596 | 4.30E-103 | |
| BEZ | ARR | SEL | FIN | -0.00199 | 4.98E-04 | | -4.00492 | 6.20E-05 | |
| BEZ | SEL | FIN | BLB | -0.0148 | 6.29E-04 | | -23.5162 | 2.78E-122 | |
| BEZ | FIN | SEL | BLB | -0.01659 | 6.14E-04 | | -27.0155 | 9.72E-161 | |
| BEZ | BLB | SEL | FIN | -0.00179 | 4.49E-04 | | -3.99441 | 6.49E-05 | |
| BEZ | SEL | FIN | OIG | -0.01204 | 6.18E-04 | | -19.4681 | 2.05E-84 | |
| BEZ | FIN | SEL | OIG | -0.01381 | 5.90E-04 | | -23.3977 | 4.51E-121 | |
| BEZ | OIG | SEL | FIN | -0.00177 | 4.44E-04 | | -3.99698 | 6.42E-05 | |
| BEZ | SEL | ARR | BLB | -0.00276 | 6.58E-04 | | -4.19469 | 2.73E-05 | |
| BEZ | ARR | SEL | BLB | 0.007864 | 7.71E-04 | | 10.20172 | 1.95E-24 | |
| BEZ | BLB | SEL | ARR | 0.010625 | 7.66E-04 | | 13.87804 | 8.61E-44 | |
| BEZ | SEL | BLB | OIG | 0.002761 | 6.28E-04 | | 4.39453 | 1.11E-05 | |
| BEZ | BLB | SEL | OIG | 0.010627 | 6.57E-04 | | 16.17135 | 8.03E-59 | |
| BEZ | OIG | SEL | BLB | 0.007866 | 6.92E-04 | | 11.36092 | 6.55E-30 | |
| BEZ | SKO | ARR | BLB | -0.00337 | 8.35E-04 | | -4.03195 | 5.53E-05 | |
| BEZ | ARR | SKO | BLB | 0.008185 | 9.60E-04 | | 8.525769 | 1.52E-17 | |
| BEZ | BLB | SKO | ARR | 0.011553 | 9.25E-04 | | 12.4855 | 8.96E-36 | |
| BEZ | SKO | BLB | OIG | 0.003375 | 7.75E-04 | | 4.353398 | 1.34E-05 | |
| BEZ | BLB | SKO | OIG | 0.011555 | 8.33E-04 | | 13.87166 | 9.41E-44 | |
| BEZ | OIG | SKO | BLB | 0.008181 | 8.60E-04 | | 9.514823 | 1.82E-21 | |
| BEZ | SWE | FIN | ARR | -0.01131 | 6.69E-04 | | -16.8982 | 4.64E-64 | |
| BEZ | FIN | SWE | ARR | -0.01324 | 6.43E-04 | | -20.5981 | 2.85E-94 | |
| BEZ | ARR | SWE | FIN | -0.00194 | 4.97E-04 | | -3.90266 | 9.51E-05 | |
| BEZ | SWE | FIN | BLB | -0.01389 | 6.27E-04 | | -22.155 | 9.34E-109 | |
| BEZ | FIN | SWE | BLB | -0.0158 | 6.10E-04 | | -25.9188 | 4.09E-148 | |
| BEZ | BLB | SWE | FIN | -0.00191 | 4.43E-04 | | -4.31959 | 1.56E-05 | |
| BEZ | SWE | FIN | OIG | -0.01128 | 6.16E-04 | | -18.3168 | 6.08E-75 | |
| BEZ | FIN | SWE | OIG | -0.01304 | 5.81E-04 | | -22.4506 | 1.26E-111 | |
| BEZ | OIG | SWE | FIN | -0.00176 | 4.53E-04 | | -3.88944 | 1.00E-04 | |
| BEZ | SWE | ARR | BLB | -0.00258 | 6.64E-04 | | -3.89121 | 9.97E-05 | |
| BEZ | ARR | SWE | BLB | 0.007917 | 7.58E-04 | | 10.44845 | 1.49E-25 | |
| BEZ | BLB | SWE | ARR | 0.0105 | 7.62E-04 | | 13.78425 | 3.17E-43 | |
| BEZ | SWE | BLB | OIG | 0.002612 | 6.33E-04 | | 4.124513 | 3.72E-05 | |
| BEZ | BLB | SWE | OIG | 0.010493 | 6.63E-04 | | 15.82852 | 1.98E-56 | |
| BEZ | OIG | SWE | BLB | 0.00788 | 6.91E-04 | | 11.40436 | 3.98E-30 | |
| BEZ | FIN | ARR | BLB | -0.00256 | 6.48E-04 | | -3.94654 | 7.93E-05 | |
| BEZ | ARR | FIN | BLB | 0.009856 | 7.68E-04 | | 12.83552 | 1.04E-37 | |
| BEZ | BLB | FIN | ARR | 0.012413 | 7.74E-04 | | 16.04271 | 6.43E-58 | |
| BEZ | FIN | BLB | OIG | 0.002765 | 6.14E-04 | | 4.506683 | 6.58E-06 | |
| BEZ | BLB | FIN | OIG | 0.012406 | 6.75E-04 | | 18.3774 | 1.99E-75 | |
| BEZ | OIG | FIN | BLB | 0.009641 | 7.07E-04 | | 13.62953 | 2.67E-42 | |
| ICL | SEL | SWE | ARR | -0.00603 | 7.71E-04 | | -7.82788 | 4.96E-15 | |
| ICL | SWE | SEL | ARR | -0.00411 | 7.71E-04 | | -5.33658 | 9.47E-08 | |
| ICL | ARR | SEL | SWE | 0.00192 | 4.99E-04 | | 3.846732 | 1.20E-04 | |
| ICL | SEL | SWE | BLB | -0.00508 | 7.34E-04 | | -6.9116 | 4.79E-12 | |
| ICL | SWE | SEL | BLB | -0.00297 | 7.31E-04 | | -4.06015 | 4.90E-05 | |
| ICL | BLB | SEL | SWE | 0.002108 | 5.06E-04 | | 4.170293 | 3.04E-05 | |
| ICL | SEL | SWE | OIG | -0.00584 | 7.02E-04 | | -8.31132 | 9.46E-17 | |
| ICL | SWE | SEL | OIG | -0.00387 | 7.16E-04 | | -5.40922 | 6.33E-08 | |
| ICL | OIG | SEL | SWE | 0.001964 | 4.76E-04 | | 4.123386 | 3.73E-05 | |
| ICL | SEL | FIN | ARR | -0.00372 | 7.69E-04 | | -4.83668 | 1.32E-06 | |
| ICL | FIN | SEL | ARR | 0.002681 | 8.00E-04 | | 3.352482 | 8.01E-04 | |
| ICL | ARR | SEL | FIN | 0.006402 | 5.91E-04 | | 10.84035 | 2.22E-27 | |
| ICL | SEL | FIN | BLB | -0.00276 | 7.21E-04 | | -3.83169 | 1.27E-04 | |
| ICL | FIN | SEL | BLB | 0.00384 | 7.72E-04 | | 4.971255 | 6.65E-07 | |
| ICL | BLB | SEL | FIN | 0.006603 | 5.96E-04 | | 11.08677 | 1.45E-28 | |
| ICL | SEL | FIN | OIG | -0.00353 | 6.94E-04 | | -5.08123 | 3.75E-07 | |
| ICL | FIN | SEL | OIG | 0.003109 | 7.30E-04 | | 4.261576 | 2.03E-05 | |
| ICL | OIG | SEL | FIN | 0.006635 | 5.67E-04 | | 11.71149 | 1.11E-31 | |
| ICL | SKO | SWE | ARR | -0.006 | 9.16E-04 | | -6.54652 | 5.89E-11 | |
| ICL | SWE | SKO | ARR | -0.00386 | 9.72E-04 | | -3.96809 | 7.24E-05 | |
| ICL | ARR | SKO | SWE | 0.002137 | 8.32E-04 | | 2.568012 | 0.010228 | |
| ICL | SKO | FIN | BLB | -0.00395 | 8.35E-04 | | -4.72843 | 2.26E-06 | |
| ICL | FIN | SKO | BLB | 0.003471 | 9.90E-04 | | 3.505701 | 4.55E-04 | |
| ICL | BLB | SKO | FIN | 0.007421 | 9.00E-04 | | 8.249107 | 1.60E-16 | |
| SKO | SWE | FIN | BLB | 0.002105 | 5.57E-04 | | 3.776172 | 1.59E-04 | |
| SKO | FIN | SWE | BLB | 0.008289 | 5.94E-04 | | 13.96224 | 2.65E-44 | |
| SKO | BLB | SWE | FIN | 0.006185 | 5.16E-04 | | 11.98001 | 4.52E-33 | |
