# Supplementary material for: Genetic diversity and historical demography of underutilised goat breeds in North-Western Europe
Source: Sci Rep. 2023 Nov 25;13:20728. doi: 10.1038/s41598-023-48005-8 (PMC10676416; doi:10.1038/s41598-023-48005-8)
Supplement: Supplementary file 14 — Supplementary Table S7. [file 41598_2023_48005_MOESM14_ESM.docx]

Supplementary Table S7. f4-ratio analysis performed with ADMIXTOOLS. Only the significant estimates are included. α is the alpha coefficient and indicates the ratio between two f4 statistics, SE is the standard error and A, O, X, B and C are the populations compared to infer the admixture proportion following the formula described in Methods.

| A | O | X | B | C | α | SE | Z-score |
| --- | --- | --- | --- | --- | --- | --- | --- |
| NRW | BEZ | ICL | SEL | BLB | 0.26 | 0.03 | 7.12 |
| NRW | BEZ | ICL | SEL | OIG | 0.23 | 0.04 | 7.15 |
| SKO | BEZ | ICL | SEL | ARR | 0.3 | 0.03 | 9.35 |
| SKO | BEZ | ICL | SEL | OIG | 0.3 | 0.03 | 9.27 |
| SKO | BEZ | ICL | SEL | BLB | 0.26 | 0.02 | 9.27 |
| SKO | BEZ | ICL | SEL | FSS | 0.25 | 0.02 | 9.29 |
| SKO | BEZ | ICL | SEL | BEY | 0.23 | 0.02 | 9.21 |
| SWE | BEZ | ICL | SEL | BLB | 0.13 | 0.03 | 4.29 |
| SWE | BEZ | ICL | SEL | OIG | 0.15 | 0.03 | 4.29 |
| MLG | BEZ | BLB | BEY | SWE | 0.48 | 0.04 | 10.71 |
| MLG | BEZ | BLB | BEY | SEL | 0.48 | 0.04 | 10.64 |
| MLG | BEZ | BLB | BEY | NRW | 0.42 | 0.04 | 10.50 |
| DNK | BEZ | BLB | SEL | FSS | 0.65 | 0.08 | 7.61 |
| DNK | BEZ | BLB | SEL | BEY | 0.47 | 0.06 | 7.47 |
| MLG | BEZ | OIG | BEY | SWE | 0.21 | 0.04 | 5.60 |
| MLG | BEZ | OIG | BEY | SEL | 0.21 | 0.04 | 5.09 |
| MLG | BEZ | OIG | BEY | NRW | 0.18 | 0.03 | 4.97 |
| DNK | BEZ | OIG | SEL | FSS | 0.21 | 0.08 | 2.49 |
| DNK | BEZ | OIG | SEL | BEY | 0.15 | 0.06 | 2.44 |
| ALP_FR | BEZ | BLB | SAA_FR | SEL | 0.2 | 0.09 | 7.21 |
| ALP_FR | BEZ | OIG | SAA_FR | SEL | 0.12 | 0.07 | 3.84 |
