# Supplementary material for: Genetic diversity and historical demography of underutilised goat breeds in North-Western Europe
Source: Sci Rep. 2023 Nov 25;13:20728. doi: 10.1038/s41598-023-48005-8 (PMC10676416; doi:10.1038/s41598-023-48005-8)
Supplement: Supplementary file 15 — Supplementary Table S8. [file 41598_2023_48005_MOESM15_ESM.docx]

Supplementary Table S8. Summary of samples included in this study. The Label is the breed code and the number of goats for each breed in indicated in brackets. Most of the genotypes are from AdaptMap project (Stella et al. 2018) and are indicated with *.

| Country | Breed | Label | Lat | Long | Dataset |
| --- | --- | --- | --- | --- | --- |
| Iceland | Icelandic Landrace | ICL (10) | 64.7 | -21.23 | * |
| Norway | Norwegian coastal (Skorpa) | SKO (6) | 69.9 | 21.68 | Berg et al. 2020 |
| Norway | Norwegian coastal (Selje) | SEL (28) | 62.08 | 5.13 | Berg et al. 2020 |
| Sweden | Swedish Landrace | SWE (43) | 63.07 | 14.21 | **This study (SMARTER)** |
| Denmark | Danish Landrace | DNK (50) | 55.33 | 10.37 | * |
| Netherlands | Dutch Landrace | NLD (15) | 52.09 | 5.12 | * |
| Finland | Finnish Landrace | FIN (19) | 62.77 | 22.56 | * |
| Ireland | Traditional Aran | ARR (9) | 53.62 | -10.21 | * |
| Ireland | Bilberry | BLB (10) | 52.25 | -7.12 | * |
| Ireland | Old Irish Goat | OIG (14) | 53.90 | -9.78 | * |
| France | Fosseé | FSS (50) | 48.35 | -1.81 | * |
| France | Alpine | ALP_FR (50) | 47.60 | -0.02 | * |
| France | Saanen | SAA_FR (50) | 46.31 | 1.55 | * |
| Switzerland | Alpine | ALP_CH (50) | 46.82 | 7.68 | * |
| Switzerland | Saanen | SAA_CH (40) | 46.98 | 7.92 | * |
| Switzerland | Toggenburg | TOG (24) | 46.82 | 7.68 | Burren et al. 2016 |
| Spain | Bermeya | BEY (23) | 43.34 | -5.26 | * |
| Spain | Malaguena | MLG (39) | 37.07 | 6.60 | * |
| Italy | Girgentana | GGT (25) | 37.61 | -4.42 | * |
| Italy | Ciociara Grigia | CCG (16) | 41.62 | 13.72 | * |
| Italy | Jonica | JON (10) | 40.69 | 15.56 | * |
| Italy | Alpine | ALP_IT (48) | 44.74 | 9.09 | * |
| Italy | Saanen | SAA_IT (23) | 46.07 | 9.57 | * |
| Tanzania | Norwegian Landrace | NRW (17) | -4.213 | 33.35 | * |
